# Factors Associated With Severe COVID-19 Among Vaccinated Adults Treated in US Veterans Affairs Hospitals

**DOI:** 10.1001/jamanetworkopen.2022.40037

**Published:** 2022-10-20

**Authors:** Austin D. Vo, Jennifer La, Julie T.-Y. Wu, Judith M. Strymish, Matthew Ronan, Mary Brophy, Nhan V. Do, Westyn Branch-Elliman, Nathanael R. Fillmore, Paul A. Monach

**Affiliations:** 1 VA Boston Cooperative Studies Program, Boston, Massachusetts; 2 VA Palo Alto Healthcare System, Palo Alto, California; 3 Stanford University School of Medicine, Stanford, California; 4 Department of Medicine, VA Boston Healthcare System, Boston, Massachusetts; 5 Harvard Medical School, Boston, Massachusetts; 6 Boston University School of Medicine, Boston, Massachusetts; 7 VA Boston Center for Healthcare Organization and Implementation Research, Boston, Massachusetts; 8 Dana Farber Cancer Institute, Boston, Massachusetts

## Abstract

**Importance:**

With a large proportion of the US adult population vaccinated against SARS-CoV-2, it is important to identify who remains at risk of severe infection despite vaccination.

**Objective:**

To characterize risk factors for severe COVID-19 disease in a vaccinated population.

**Design, Setting, and Participants:**

This nationwide, retrospective cohort study included US veterans who received a SARS-CoV-2 vaccination series and later developed laboratory-confirmed SARS-CoV-2 infection and were treated at US Department of Veterans Affairs (VA) hospitals. Data were collected from December 15, 2020, through February 28, 2022.

**Exposures:**

Demographic characteristics, comorbidities, immunocompromised status, and vaccination-related variables.

**Main Outcomes and Measures:**

Development of severe vs nonsevere SARS-CoV-2 infection. Severe disease was defined as hospitalization within 14 days of a positive SARS-CoV-2 diagnostic test and either blood oxygen level of less than 94%, receipt of supplemental oxygen or dexamethasone, mechanical ventilation, or death within 28 days. Association between severe disease and exposures was estimated using logistic regression models.

**Results:**

Among 110 760 patients with infections following vaccination (97 614 [88.1%] men, mean [SD] age at vaccination, 60.8 [15.3] years; 26 953 [24.3%] Black, 11 259 [10.2%] Hispanic, and 71 665 [64.7%] White), 10 612 (9.6%) had severe COVID-19. The strongest association with risk of severe disease after vaccination was age, which increased among patients aged 50 years or older with an adjusted odds ratio (aOR) of 1.42 (CI, 1.40-1.44) per 5-year increase in age, such that patients aged 80 years or older had an aOR of 16.58 (CI, 13.49-20.37) relative to patients aged 45 to 50 years. Immunocompromising conditions, including receipt of different classes of immunosuppressive medications (eg, leukocyte inhibitor: aOR, 2.80; 95% CI, 2.39-3.28) or cytotoxic chemotherapy (aOR, 2.71; CI, 2.27-3.24) prior to breakthrough infection, or leukemias or lymphomas (aOR, 1.87; CI, 1.61-2.17) and chronic conditions associated with end-organ disease, such as heart failure (aOR, 1.74; CI, 1.61-1.88), dementia (aOR, 2.01; CI, 1.83-2.20), and chronic kidney disease (aOR, 1.59; CI, 1.49-1.69), were also associated with increased risk. Receipt of an additional (ie, booster) dose of vaccine was associated with reduced odds of severe disease (aOR, 0.50; CI, 0.44-0.57).

**Conclusions and Relevance:**

In this nationwide, retrospective cohort of predominantly male US Veterans, we identified risk factors associated with severe disease despite vaccination. Findings could be used to inform outreach efforts for booster vaccinations and to inform clinical decision-making about patients most likely to benefit from preexposure prophylaxis and antiviral therapy.

## Key Points

**Question:**

What are risk factors for severe breakthrough SARS-CoV-2 infections among vaccinated individuals?

**Findings:**

In this cohort study of 110 760 vaccinated US veterans, increasing age was most strongly associated with severe disease, with risk increasing steadily among patients older than 50 years. Immunocompromising conditions and comorbidities indicating chronic heart, lung, kidney, or neurologic damage also increased risk, with a magnitude similar to or less than a 10-year age increase.

**Meaning:**

Identification of the risk factors for severe breakthrough COVID-19 could be used to guide policies and decision-making about preventive measures for those who remain at risk of disease progression despite vaccination.

## Teaser

This cohort study characterizes risk factors for severe COVID-19 disease in a vaccinated population.

## Introduction

The 2-dose mRNA vaccine series BNT162b2 (Pfizer/BioNTech) and mRNA-1273 (Moderna) and the adenoviral vaccine Ad26.COV2.S (Johnson & Johnson) are effective for reducing risk of severe COVID-19 disease and death. So-called breakthrough infections in vaccinated persons have been observed since mid-2021 and continued to increase following Omicron predominance. Substantial protection against severe outcomes following vaccination and boosting is maintained for most of the population^1,2,3^; however, some patients remain at risk of severe infections following vaccination, leading to hospitalizations for COVID-19 management and death.

Identifying patients who remain at risk for severe disease despite vaccination can help inform efforts to promote booster vaccination and guide distribution of antiviral drugs for early treatment or preexposure prophylaxis. Thus, the primary aim of this national, retrospective cohort study was to identify risk factors associated with severe disease among vaccinated US veterans who developed infections following vaccination. Secondary aims were to identify and quantify risk factors in subgroups that may have different risk profiles (stratifying by immunocompromised status, age, sex, and periods with Delta or Omicron variant predominance) and to evaluate whether associations with reductions in disease severity waned over time since the primary vaccination series and a single booster dose.

## Methods

This article follows the Strengthening the Reporting of Observational Studies in Epidemiology (STROBE) reporting guideline (eMethods in the Supplement). This study was approved by the VA Boston Research and Development committee as an exempt study prior to data collection and analysis with a waiver of informed consent due to the use of existing data, per the Common Rule.

### Cohort Creation

The cohort consisted of all patients who received a complete initial vaccination series (2 doses of an mRNA vaccine or 1 dose of Ad26.COV2.S) before December 1, 2021, and subsequently tested positive for SARS-CoV-2 infection (polymerase chain reaction or antigen) within the Veterans Affairs Healthcare System (VA) through February 28, 2022. The study period was December 15, 2020, to February 28, 2022, which includes intervals during which the original strain, Delta variant (July 1 to December 15, 2021), and Omicron variant (December 16, 2021, to February 8, 2022) were predominant. Full methods are outlined in eMethods in the Supplement.

### Data Sources and Uses

Data were obtained from the VA COVID-19 shared data resource^4^ and the Corporate Data Warehouse (CDW). eTable 1 in the Supplement presents a complete listing of definitions and data sources.

The primary outcome was severe breakthrough infection, defined by death 2 to 28 days after a positive test or medical or surgical hospitalization within 14 days of the positive test with documented blood oxygen level of less than 94%, receipt of any supplemental oxygen, receipt of dexamethasone,^5,6^ or receipt of mechanical ventilation. The comparator was nonsevere breakthrough infection, defined as no hospitalization or hospitalization that did not meet criteria for severe disease.

Demographic data included age in years at the time of breakthrough infection (modeled in 5-year increments), biologic sex, race (VA-standard categories for voluntary self-reporting as American Indian or Alaska Native, Asian, Black or African American, Native Hawaiian or other Pacific Islander, or White) and ethnicity (Hispanic or Latino, yes or no), US region, and urban or rural residence defined by US Census criteria. Vaccination data included dates of each vaccination dose and the vaccine manufacturer. Occurrence of a previous SARS-CoV-2 infection before vaccination was defined as a positive test any time before day 14 after completing the initial vaccination series.

Comorbidities recorded in the patients’ records at the time of infection were assessed using the Chronic Conditions Warehouse during the 3 years prior to vaccination.^7^ Body mass index (BMI) was calculated as weight in kilograms divided by height in meters squared, with US averages used to estimate height if the variable was not available in the electronic health record.

### Definitions of Immunocompromised Status

Drugs known to increase risk of infectious diseases were classified into 5 categories: cytotoxic chemotherapy, glucocorticoids (prednisone or methylprednisolone), cytokine-blocking drugs (eg, anti–tumor necrosis factor, anti–interleukin 6 receptor, anti–interleukin 17), leukocyte-inhibiting drugs (eg, methotrexate, azathioprine, JAK inhibitors, abatacept), and lymphocyte-depleting drugs (eg, rituximab). Receipt of immunosuppressive drugs was assessed shortly before vaccination and after vaccination but shortly before breakthrough infection. Different time intervals were used for different drug classes and based on their expected duration of impact. eTable 2 in the Supplement includes details of classifications and definitions. The immunocompromised subcohort also included patients with leukemia or lymphoma but not patients with HIV, based on results of the univariate analysis.

### Time Since Vaccination

Interpreting time since vaccination as a risk factor for severe disease may be challenging because of high potential for residual confounding and effect modification due to earlier availability of vaccines for patients with higher risk, differential use and timing of boosting, and development of the Delta and then the Omicron variants. Thus, in addition to assessing risk of severe breakthrough infection as a function of time since initial vaccination and time since boosting, time since vaccination was also assessed among patients who did not receive boosters.

### Statistical Analysis

**Figure 1.**
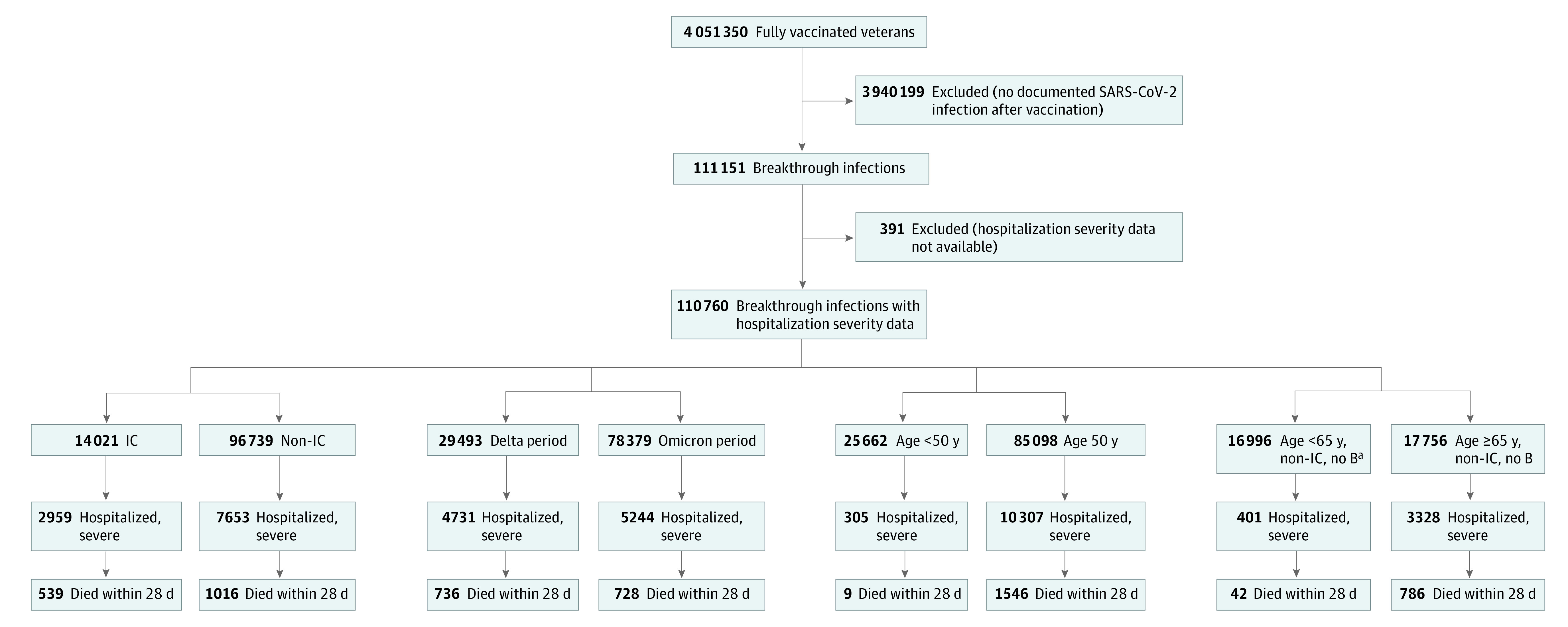
Cohorts and Subcohorts Included in the Study, With Results for Total Breakthrough Infections, Severe Breakthrough Infections, and Deaths 2 to 28 Days After Diagnosis of Infection The Methods section and eMethods in the Supplement include the definition of immunocompromised (IC) status, time periods for Delta and Omicron periods, and time periods for US Food and Drug Administration approval of vaccination for the general adult population. ^a^In addition to no booster (B), started initial vaccination on or after recommendation for all US adults (March 29, 2021).

The primary analysis evaluated exposures associated with the development of severe vs nonsevere SARS-CoV-2 infection despite vaccination. Secondary stratified analyses were conducted on subcohorts (Figure 1) to further explore the associations of an immunocompromised state, age, and time since vaccination with severe vs nonsevere breakthrough infection.

Multivariable logistic regression was used to assess the association of the binary outcome (severe vs nonsevere breakthrough SARS-CoV-2 infection) with the set of independent variables incorporating demographic characteristics, vaccination status, immunocompromised states, and comorbidities, as described previously. Variables determined a priori to be of interest and those with adjusted odds ratios (aOR) of 1.50 or greater or 0.67 or less and statistical significance are presented in the main results; cutoffs were selected based on our goal of identifying associations of potential and likely clinical significance.^8,9^ Results that include all variables are provided in supplementary tables. Findings were considered statistically significant at a 2-tailed *P* < .05 after adjustment for multiple comparisons using the Benjamini-Hochberg method. All analyses were conducted with R version 4.0.2 (R Project for Statistical Computing).

## Results

### Description of Full Cohort

From December 15, 2020, to February 28, 2022, 111 151 patients had a SARS-CoV-2 infection following vaccination, with severity assessments available for 110 760 (97 614 [88.1%] male, mean [SD] age at vaccination, 60.8 [15.4] years, 26 953 [24.3%] Black, 11 259 [10.2%] Hispanic, and 71 665 [64.7%] White, evenly distributed throughout US regions). Data for all variables that were analyzed are in eTable 3 in the Supplement. Table 1 includes data for variables meeting predefined cutoffs and others of particular clinical interest. eTable 4 in the Supplement shows additional categorical separation of severities.

**Table 1.** Demographic, Clinical, and Vaccination-Related Data for Patients With Severe and Nonsevere COVID-19 Despite Vaccination

Characteristic	Patients, No. (%)			aOR (95% CI)^a^
	Overall (N = 110 760)	Nonsevere (n = 100 148)	Severe (n = 10 612)	
Sex				
Male	97 614 (88.1)	87 389 (87.3)	10 225 (96.4)	1 [Reference]
Female	13 146 (11.9)	22 759 (22.7)	387 (3.6)	0.67 (0.60-0.75)
Age ranges, y				
<40	13 114 (11.8)	13 004 (13.0)	110 (1.0)	0.57 (0.44-0.75)
40-44	6241 (5.6)	6153 (6.1)	88 (0.8)	0.88 (0.66-1.18)
45-49	6307 (5.7)	6200 (6.2)	107 (1.0)	1 [Reference]
50-54	9721 (8.8)	9444 (9.4)	277 (2.6)	1.60 (1.27-2.01)
55-59	10 934 (9.9)	10 458 (10.4)	476 (4.5)	2.24 (1.80-2.78)
60-64	12 971 (11.7)	12 082 (12.1)	889 (8.4)	3.24 (2.64-3.99)
65-69	12 353 (11.2)	11 013 (11.0)	1340 (12.6)	4.82 (3.93-5.92)
70-74	18 327 (16.5)	15 703 (15.7)	2624 (24.7)	6.63 (5.42-8.11)
75-79	11 562 (10.4)	9546 (9.5)	2016 (19.0)	8.72 (7.10-10.7)
≥80	9230 (8.3)	6545 (6.5)	2685 (25.3)	16.58 (13.49-20.37)
Dominant variant				
Pre-Delta period	2888 (2.6)	2251 (2.2)	637 (6.0)	1 [Reference]
Delta period	29 493 (26.6)	24 762 (24.7)	4731 (44.6)	0.95 (0.83-1.08)
Omicron period	78 379 (70.8)	73 135 (73.0)	5244 (49.4)	0.49 (0.42-0.56)
Vaccine type				
Ad26.COV2.S	11 066 (10.0)	10 028 (10.0)	1038 (9.8)	1.30 (1.20-1.41)
mRNA-1273	45 530 (41.1)	41 289 (41.2)	4241 (40.0)	0.79 (0.75-0.83)
BNT162b2	54 164 (48.9)	48 831 (48.8)	5333 (50.3)	1 [Reference]
History of infection before vaccinated	4373 (3.9)	3959 (4.0)	414 (3.9)	0.69 (0.61-0.78)
Time since fully vaccinated at breakthrough, mo				
<4	12 497 (11.3)	10 983 (11.0)	1514 (14.3)	1 [Reference]
4 to <5	7216 (6.5)	6355 (6.3)	861 (8.1)	1.06 (0.95-1.18)
5 to <6	8148 (7.4)	7039 (7.0)	1109 (10.5)	1.01 (0.91-1.12)
6 to <7	8902 (8.0)	7763 (7.8)	1139 (10.7)	1.08 (0.97-1.20)
7 to <8	11 739 (10.6)	10 665 (10.6)	1074 (10.1)	1.13 (1.02-1.26)
8 to <9	19 118 (17.3)	17 873 (17.8)	1245 (11.7)	1.15 (1.03-1.28)
9 to <10	19 997 (18.1)	18 570 (18.5)	1427 (13.4)	1.16 (1.03-1.30)
10 to <11	15 731 (14.2)	14 252 (14.2)	1479 (13.9)	1.34 (1.19-1.51)
11 to <12	6519 (5.9)	5853 (5.8)	666 (6.3)	1.47 (1.28-1.69)
≥12	893 (0.8)	795 (0.8)	98 (0.9)	1.57 (1.22-2.04)
Times since booster at breakthrough, mo				
Not boosted	81 039 (73.2)	72 483 (72.4)	8556 (80.6)	1 [Reference]
<1	5755 (5.2)	5401 (5.4)	354 (3.3)	0.56 (0.49-0.63)
1 to <2	7732 (7.0)	7341 (7.3)	391 (3.7)	0.43 (0.38-0.48)
2 to <3	8550 (7.7)	7996 (8.0)	554 (5.2)	0.46 (0.41-0.51)
3 to <4	4633 (4.2)	4197 (4.2)	436 (4.1)	0.54 (0.48-0.61)
4 to <5	1749 (1.6)	1543 (1.5)	206 (1.9)	0.59 (0.50-0.70)
5 to <6	370 (0.3)	320 (0.3)	50 (0.5)	0.68 (0.48-0.95)
≥6	932 (0.8)	867 (0.9)	65 (0.6)	0.78 (0.59-1.03)
IS medications before breakthrough				
Chemotherapy	1006 (0.9)	696 (0.7)	310 (2.9)	2.71 (2.27-3.24)
Cytokine-blocking	1601 (1.4)	1401 (1.4)	200 (1.9)	1.66 (1.32-2.09)
Glucocorticoids	7544 (6.8)	5723 (5.7)	1821 (17.2)	2.34 (2.18-2.50)
Leukocyte-inhibitory	1924 (1.7)	1438 (1.4)	486 (4.6)	2.80 (2.39-3.28)
Lymphocyte-depleting	585 (0.5)	406 (0.4)	179 (1.7)	2.07 (1.57-2.72)
IS medications before initial vaccination				
Chemotherapy	430 (0.4)	316 (0.3)	114 (1.1)	0.86 (0.65-1.15)
Cytokine-blocking	1211 (1.1)	1096 (1.1)	115 (1.1)	0.60 (0.45-0.80)
Glucocorticoids	2754 (2.5)	2124 (2.1)	630 (5.9)	1.38 (1.23-1.54)
Leukocyte-inhibitory	1368 (1.2)	1099 (1.1)	269 (2.5)	0.80 (0.65-0.98)
Lymphocyte-depleting	223 (0.2)	153 (0.2)	70 (0.7)	1.07 (0.69-1.65)
BMI class				
Underweight, <18.5	677 (0.6)	496 (0.5)	181 (1.7)	1.53 (1.24-1.87)
Normal, 18 to <25	14 394 (13.0)	12 299 (12.3)	2095 (19.7)	1 [Reference]
Overweight, 25 to <30	32 707 (29.5)	29 792 (29.7)	2915 (27.5)	0.71 (0.67-0.76)
Obesity I, 30 to <35	31 156 (28.1)	28 557 (28.5)	2599 (24.5)	0.78 (0.73-0.84)
Obesity II, 35 to <40	16 862 (15.2)	15 335 (15.3)	1527 (14.4)	0.94 (0.87-1.02)
Severe obesity, ≥40	10 641 (9.6)	9510 (9.5)	1131 (10.7)	1.23 (1.12-1.35)
Unknown	4323 (3.9)	4159 (4.2)	164 (1.5)	0.88 (0.74-1.06)
Comorbidities				
Alzheimers disease and related disorders or senile dementia	3050 (2.8)	1915 (1.9)	1135 (10.7)	2.01 (1.83-2.20)
Chronic kidney disease	11 832 (10.7)	9071 (9.1)	2761 (26.0)	1.59 (1.49-1.69)
COPD and bronchiectasis	8337 (7.5)	6103 (6.1)	2234 (21.1)	1.65 (1.54-1.76)
Diabetes	26 083 (23.5)	21 919 (21.9)	4164 (39.2)	1.25 (1.19-1.32)
Heart failure	5444 (4.9)	3681 (3.7)	1763 (16.6)	1.74 (1.61-1.88)
HIV or AIDS	874 (0.8)	791 (0.8)	83 (0.8)	1.30 (1.01-1.68)
Leukemias and lymphomas	1336 (1.2)	993 (1.0)	343 (3.2)	1.87 (1.61-2.17)
Lung cancer	824 (0.7)	573 (0.6)	251 (2.4)	1.61 (1.36-1.92)
Mobility impairments	1030 (0.9)	728 (0.7)	302 (2.8)	1.92 (1.63-2.26)
Multiple sclerosis and transverse myelitis	454 (0.4)	362 (0.4)	92 (0.9)	2.86 (2.17-3.78)
Pressure and chronic ulcers	1347 (1.2)	872 (0.9)	475 (4.5)	1.58 (1.37-1.81)
Schizophrenia and other psychotic disorders	2755 (2.5)	2320 (2.3)	435 (4.1)	1.71 (1.51-1.93)

Abbreviations: aOR, adjusted odds ratio; BMI, body mass index class (calculated as weight in kilograms divided by height in meters squared); COPD, chronic obstructive pulmonary disease; IS, immune-suppressive.

^a^ Results of multivariable logistic regression are also shown. Results for all variables used in the multivariable analysis are shown in eTable 3 in the Supplement.

Overall, 29 493 breakthrough infections (26.6%) occurred during the Delta period (July 1 to December 15, 2021) and 78 379 (70.8%) during the Omicron period (December 16, 2021, to February 28, 2022), 4373 patients (3.9%) had evidence of prior SARS-CoV-2 infection before vaccination, and 29 721 (26.8%) were boosted prior to breakthrough infection. The 110 760 breakthrough infections were associated with 10 612 severe cases (9.6%) and 1555 deaths (1.4%). In the Delta period, 4731 cases (16.0%) were severe and 736 (2.5%) were associated with death, compared with 5244 (6.7%) and 728 (0.9%) during the Omicron period.

### Full Cohort Analysis

**Figure 2.**
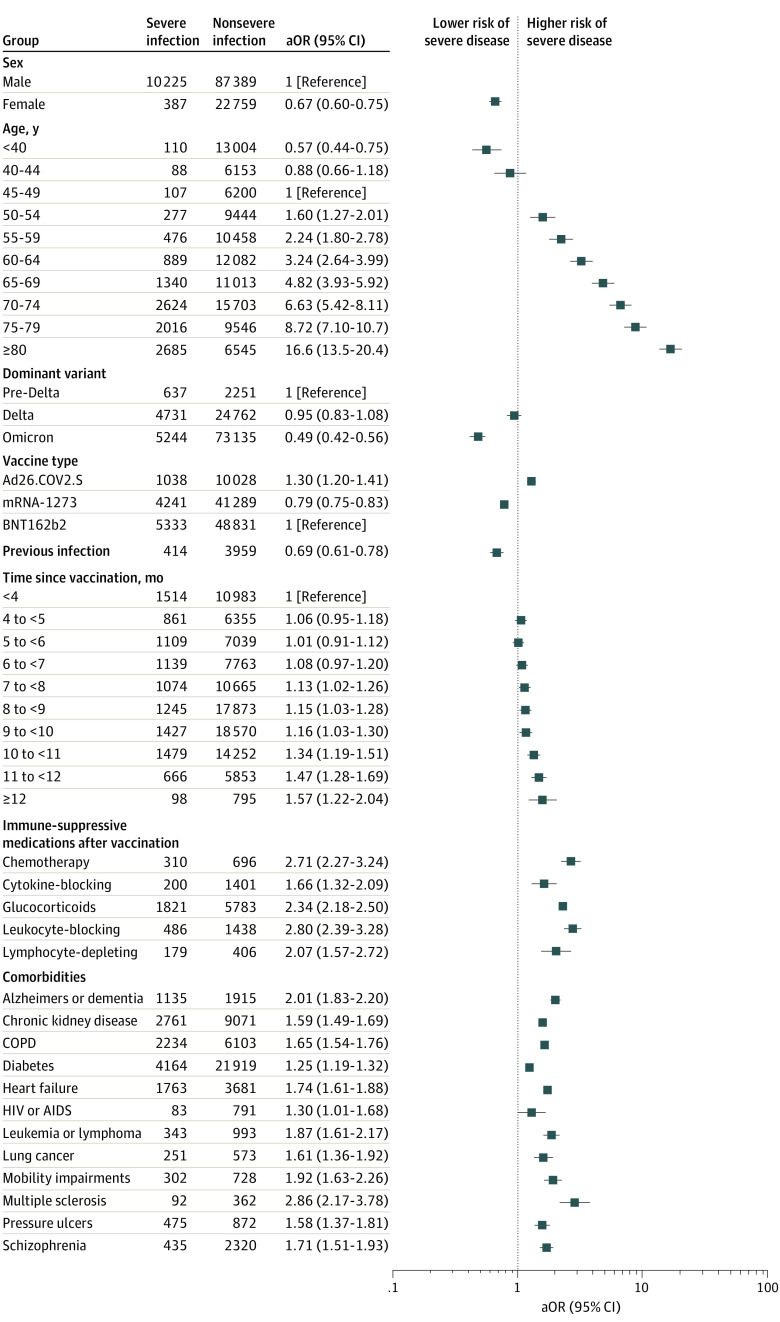
Risk of Severe vs Nonsevere Breakthrough Infection All variables that met significance criteria (*P* < .05 after adjustment for multiple comparisons, and adjusted odds ratio [aOR] ≥1.50 or ≤0.67) and selected additional variables (eg, diabetes and HIV infection) chosen a priori for comparison.

Multivariable-adjusted estimates of associations with severe disease are presented in Table 1 and Figure 2. eTable 3 in the Supplement includes all variables, regardless of magnitude of effect size, and results of univariable and multivariable logistic regressions. The variable with the strongest association with severe COVID-19 outcomes was age; risk rose steadily at least above age 50 years (aOR per 5-year increase, 1.42; 95% CI, 1.40-1.44), such that the aOR was 16.58 (CI, 13.49-20.37) for patients aged 80 years or older compared with patients aged 45 to 50 years. Associations with severe disease were somewhat higher among Native American than White patients (aOR, 1.33; 95% CI, 1.04-1.71). Female sex was associated with lower odds of severe outcomes than male sex (aOR, 0.67; 95% CI, 0.60-0.75). Compared with receipt of the BNTb162b2 vaccine, receipt of the mRNA-1273 vaccine was associated with lower odds of severe disease (aOR, 0.79; 95% CI, 0.75-0.83). Receipt of 1 dose of Ad26.COV2.S was associated with higher odds (aOR, 1.30; 95% CI, 1.20-1.41).

Comorbidities in many different organ systems (cardiac, pulmonary, renal, and neurologic/psychiatric) were associated with a similar magnitude of increased odds (aORs between 1.59 and 2.86 for the 8 highest-risk conditions). Comorbidities that reflected permanent organ damage (eg, heart failure [aOR, 1.74; CI, 1.61-1.88], chronic obstructive pulmonary disease [COPD; aOR, 1.65; 95% CI, 1.54-1.76], chronic kidney disease [aOR, 1.59; CI, 1.49-1.69], dementia [aOR, 2.01; CI, 1.83-2.20]) were associated with greater magnitudes of increased risk of disease than predisposing conditions, such as diabetes and hypertension.

### Patients Younger Than 50 Years

In the subcohort limited to patients younger than 50 years, overall risk of severe disease was low (305 of 25 662 patients [1.2%]), and death was rare (9 patients [0.04%]). The low number of severe cases in this group limits interpretability; however, a small set of severe chronic health problems met criteria for statistical significance (eTable 5 and eAppendix in the Supplement).

### Immunocompromised Cohort

Overall, 14 021 patients with breakthrough infections met criteria for being immunocompromised at the time of vaccination or breakthrough. HIV infection was not included due to a nonsignificant and modest-magnitude association with severe breakthrough COVID-19. Demographic and clinical data for the immunocompromised cohort, stratified by severity, are shown in Table 2 and eTable 6 in the Supplement. Immunocompromised patients who developed breakthrough infections had higher absolute unadjusted risk of severe disease (2959 [21.1%]) or death (539 [3.8%]) than patients without immunocompromising conditions or medications (7653 [7.9%] and 1016 [1.1%], respectively, of 96 739). Rates of severe disease and death among immunocompromised patients were 1276 of 4422 (28.9%) and 250 (5.7%), respectively, in the Delta period and 1481 of 9098 (16.3%) and 245 (2.7%), respectively, in the Omicron period. Additional risk factors for severe disease among immunocompromised patients (eg, age and specific comorbidities) were very similar to those for the whole population (Table 2; eTable 6 in the Supplement).

**Table 2.** Demographic, Clinical, and Vaccination-Related Data, and Results of Multivariable Logistic Regression for Immunocompromised Patients With Severe and Non-Severe COVID-19 Despite Vaccination

Characteristic	Patients, No. (%)			aOR (95% CI)^a^
	Overall (n = 14 021)	Nonsevere infection (n = 11 062)	Severe infection (n = 2959)	
Sex				
Male	12 505 (89.2)	9653 (87.3)	2852 (96.4)	1 [Reference]
Female	1516 (10.8)	1409 (12.7)	107 (3.6)	0.59 (0.47-0.74)
Age range, y				
<40	848 (6.0)	829 (7.5)	19 (0.6)	0.69 (0.37-1.29)
40-44	524 (3.7)	506 (4.6)	18 (0.6)	0.98 (0.52-1.86)
45-49	636 (4.5)	613 (5.5)	23 (0.8)	1 [Reference]
50-54	1004 (7.2)	925 (8.4)	79 (2.7)	2.06 (1.27-3.36)
55-59	1228 (8.8)	1093 (9.9)	135 (4.6)	2.52 (1.58-4.02)
60-64	1733 (12.4)	1467 (13.3)	266 (9.0)	3.51 (2.24-5.51)
65-69	1874 (13.4)	1452 (13.1)	422 (14.3)	5.01 (3.21-7.83)
70-74	2955 (21.1)	2122 (19.2)	833 (28.2)	6.79 (4.37-10.6)
75-79	1841 (13.1)	1274 (11.5)	567 (19.2)	7.71 (4.93-12.1)
≥80	1378 (9.8)	781 (7.1)	597 (20.2)	12.66 (8.04-19.93)
Dominant variant				
Pre-Delta period	501 (3.6)	299 (2.7)	202 (6.8)	1 [Reference]
Delta period	4422 (31.5)	3146 (28.4)	1276 (43.1)	0.74 (0.57-0.96)
Omicron period	9098 (64.9)	7617 (68.9)	1481 (50.1)	0.44 (0.33-0.58)
Vaccine type				
Ad26.COV2.S	1172 (8.4)	940 (8.5)	232 (7.8)	1.11 (0.93-1.33)
mRNA-1273	6098 (43.5)	4860 (43.9)	1238 (41.8)	0.78 (0.71-0.86)
BNT162b2	6751 (48.1)	5262 (47.6)	1489 (50.3)	1 [Reference]
History of infection before vaccinated	636 (4.5)	515 (4.7)	121 (4.1)	0.61 (0.48-0.78)
Time since fully vaccinated at breakthrough, mo				
<4	1672 (11.9)	1225 (11.1)	447 (15.1)	1 [Reference]
4 to <5	975 (7.0)	743 (6.7)	232 (7.8)	1.09 (0.86-1.37)
5 to <6	1159 (8.3)	831 (7.5)	328 (11.1)	1.14 (0.91-1.41)
6 to <7	1140 (8.1)	845 (7.6)	295 (10.0)	1.04 (0.83-1.30)
7 to <8	1336 (9.5)	1083 (9.8)	253 (8.6)	0.93 (0.74-1.17)
8 to <9	2069 (14.8)	1759 (15.9)	310 (10.5)	0.99 (0.79-1.24)
9 to <10	2488 (17.7)	2070 (18.7)	418 (14.1)	1.13 (0.90-1.42)
10 to <11	2160 (15.4)	1709 (15.4)	451 (15.2)	1.33 (1.04-1.70)
11 to <12	915 (6.5)	710 (6.4)	205 (6.9)	1.39 (1.05-1.85)
≥12	107 (0.8)	87 (0.8)	20 (0.7)	0.94 (0.53-1.68)
Time since boosted at breakthrough, mo				
Not boosted	9408 (67.1)	7234 (65.4)	2174 (73.5)	1 [Reference]
1 or less	663 (4.7)	541 (4.9)	122 (4.1)	0.78 (0.62-0.99)
1 to <2	878 (6.3)	734 (6.6)	144 (4.9)	0.63 (0.51-0.78)
2 to <3	1107 (7.9)	903 (8.2)	204 (6.9)	0.69 (0.57-0.83)
3 to <4	1040 (7.4)	883 (8.0)	157 (5.3)	0.51 (0.41-0.63)
4 to <5	670 (4.8)	563 (5.1)	107 (3.6)	0.54 (0.42-0.70)
5 to <6	141 (1.0)	109 (1.0)	32 (1.1)	0.78 (0.49-1.23)
≥6	114 (0.8)	95 (0.9)	19 (0.6)	0.81 (0.47-1.41)
IS medications before breakthrough				
Chemotherapy	1006 (7.2)	696 (6.3)	310 (10.5)	2.21 (1.83-2.67)
Cytokine-blocking	1601 (11.4)	1401 (12.7)	200 (6.8)	1.41 (1.12-1.78)
Glucocorticoids	7544 (53.8)	5723 (51.7)	1821 (61.5)	1.91 (1.70-2.14)
Leukocyte-inhibitory	1924 (13.7)	1438 (13.0)	486 (16.4)	2.42 (2.05-2.85)
Lymphocyte-depleting	585 (4.2)	406 (3.7)	179 (6.0)	1.94 (1.47-2.54)
IS medications before initial vaccination				
Chemotherapy	430 (3.1)	316 (2.9)	114 (3.9)	0.87 (0.66-1.15)
Cytokine-blocking	1211 (8.6)	1096 (9.9)	115 (3.9)	0.59 (0.45-0.79)
Glucocorticoids	2754 (19.6)	2124 (19.2)	630 (21.3)	1.22 (1.08-1.38)
Leukocyte-inhibitory	1368 (9.8)	1099 (9.9)	269 (9.1)	0.78 (0.64-0.95)
Lymphocyte-depleting	223 (1.6)	153 (1.4)	70 (2.4)	1.12 (0.73-1.70)
BMI class				
Underweight	119 (0.8)	60 (0.5)	59 (2.0)	1.67 (1.11-2.53)
Normal	1994 (14.2)	1372 (12.4)	622 (21.0)	1 [Reference]
Overweight	4085 (29.1)	3271 (29.6)	814 (27.5)	0.65 (0.57-0.75)
Obesity I	3883 (27.7)	3155 (28.5)	728 (24.6)	0.69 (0.60-0.80)
Obesity II	2204 (15.7)	1798 (16.3)	406 (13.7)	0.76 (0.64-0.90)
Severe obesity	1568 (11.2)	1252 (11.3)	316 (10.7)	0.93 (0.77-1.12)
Unknown	168 (1.2)	154 (1.4)	14 (0.5)	0.55 (0.30-1.01)
Comorbidities				
Alzheimers disease and related disorders or senile dementia	450 (3.2)	228 (2.1)	222 (7.5)	1.51 (1.21-1.88)
Chronic kidney disease	2285 (16.3)	1441 (13.0)	844 (28.5)	1.57 (1.38-1.78)
COPD and bronchiectasis	2489 (17.8)	1539 (13.9)	950 (32.1)	1.71 (1.52-1.92)
Diabetes	3931 (28.0)	2800 (25.3)	1131 (38.2)	1.22 (1.10-1.36)
Heart failure	1205 (8.6)	675 (6.1)	530 (17.9)	1.51 (1.29-1.77)
HIV and/or AIDS	103 (0.7)	83 (0.8)	20 (0.7)	0.98 (0.56-1.70)
Leukemias and lymphomas	1336 (9.5)	993 (9.0)	343 (11.6)	1.58 (1.33-1.88)
Lung cancer	289 (2.1)	173 (1.6)	116 (3.9)	1.38 (1.05-1.80)
Mobility impairments	166 (1.2)	94 (0.8)	72 (2.4)	1.73 (1.20-2.51)
Multiple sclerosis and transverse myelitis	207 (1.5)	166 (1.5)	41 (1.4)	2.02 (1.31-3.11)
Pressure and chronic ulcers	235 (1.7)	117 (1.1)	118 (4.0)	1.68 (1.24-2.29)
Schizophrenia and other psychotic disorders	327 (2.3)	236 (2.1)	91 (3.1)	1.41 (1.06-1.88)

Abbreviations: aOR, adjusted odds ratio; BMI, body mass index (calculated as weight in kilograms divided by height in meters squared); COPD, chronic obstructive pulmonary disease; IS, immune-suppressive.

^a^ Results for all variables used in the analysis are shown in eTable 6 in the Supplement.

No specific class of immunosuppressive drugs was clearly associated with a higher magnitude of increased odds, but leukocyte inhibitor before breakthrough infection conferred more risk than the drug class with lowest risk (leukocyte inhibitor: aOR, 2.80; 95% CI, 2.39-3.28; cytokine blocking: aOR, 1.66; 95% CI, 1.32-2.09). In multivariable analyses, receipt of immunosuppressive drugs after vaccination (before breakthrough) was associated with severe COVID-19, whereas magnitude of risk associated with receipt before vaccination was much lower (Tables 1 and 2; eTables 3 and 6 in the Supplement); among 1950 patients receiving immunosuppressive drugs only prior to initial vaccination, 263 (13.5%) had severe disease, in contrast to 1767 of 7896 patients (22.4%) immunosuppressed only at the time of the breakthrough infection, and 730 of 3264 patients (22.4%) immunosuppressed both before and after vaccination (eTable 7 in the Supplement). Overall, 1053 of 14 021 immunocompromised patients (7.5%) were receiving at least 2 different classes of immunosuppressive drugs between vaccination and breakthrough. When compared with those receiving only 1 immunosuppressive drug class, receipt of multiple immunosuppressive drugs was associated with higher odds of severe disease (2 drugs: aOR, 1.92; 95% CI, 1.64-2.26; 3 drugs: aOR, 2.53; 95% CI, 1.69-3.80) (eTable 8 in the Supplement).

### Time Since Vaccination or Booster

**Figure 3.**
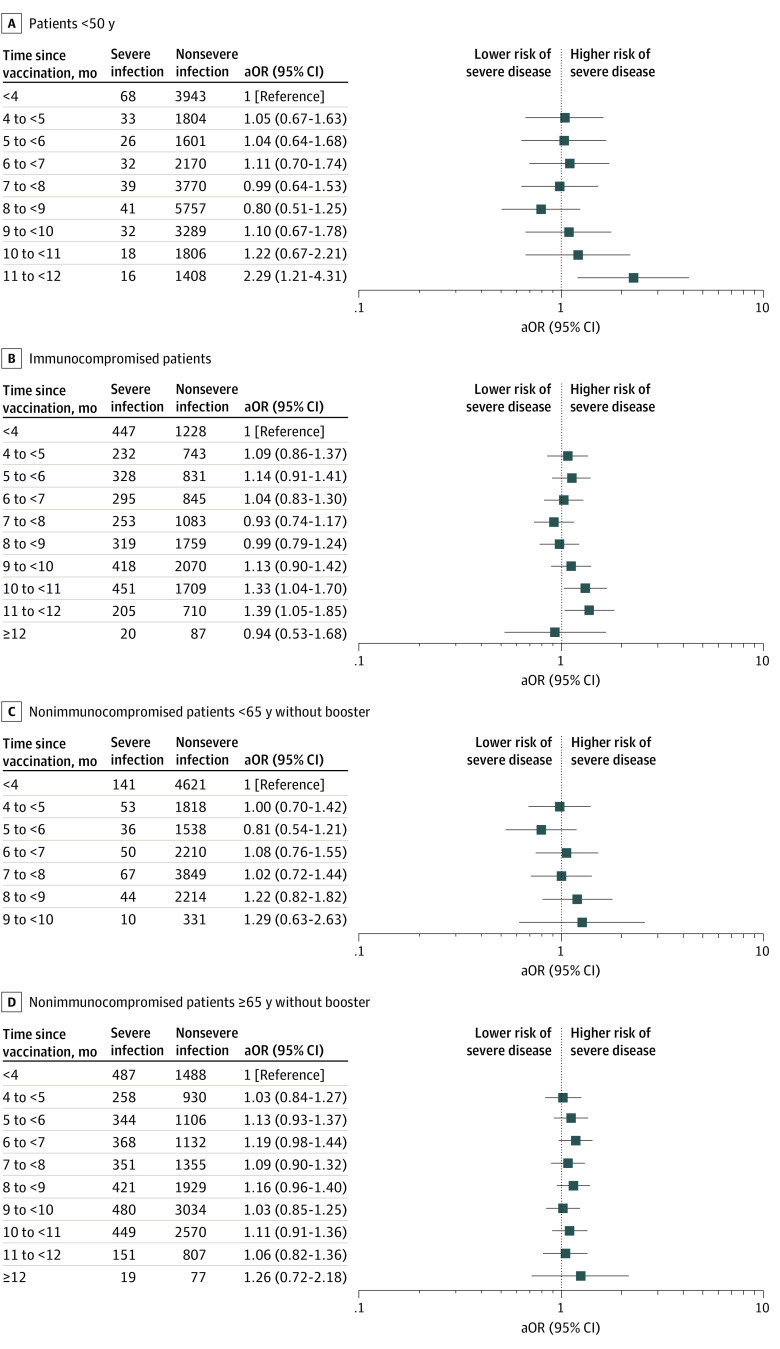
Risk of Severe vs Nonsevere Breakthrough Infection as a Function of Time Since Vaccination in Subcohorts Defined by Age, Vaccine Eligibility, and Immunocompromise The focus of the plots is on time since initial vaccination, but results are derived from multivariable logistic regression. aOR indicates adjusted odds ratio.

Boosting was associated with a decreased risk of severe breakthrough in the main analysis (aOR, 0.50; 95% CI, 0.44-0.57) and in all subsets, as was prior SARS-CoV-2 infection (aOR, 0.69; 95% CI, 0.61-0.78). In the unstratified full-cohort analysis, time since vaccination and time since booster appeared to be associated with small but steady increases in risk of severe disease, suggesting waning vaccine effectiveness (Table 1; eTable 3 in the Supplement), but these findings varied among subsets (Figure 3; eTables 5, 6, 9 and 10 in the Supplement). However, when interpretability was improved by stratification by boosting status and indication for early vaccination, decreasing effectiveness was not observed, neither in patients younger or older than 65 years nor in immunocompromised patients (Figure 3; eTables 6, 11, and 12 in the Supplement), suggesting residual confounding in the full-cohort analysis.

### Analyses of Additional Subsets

Female patients were younger (eFigure in the Supplement) and had lower incidence of severe breakthrough infection, even after adjusting for age (Table 1; eTable 3 in the Supplement). In an analysis limited to female patients, risk factors were similar to those in male patients (eTables 13-15 in the Supplement). Similarly, risk factors for severe disease were similar during the Delta and Omicron periods (eTables 16-18 in the Supplement) and did not appear to differ based on whether patients initially received the BNT162b2 or mRNA-1273 vaccine (eTables 19 and 20 in the Supplement) or resided in urban vs rural locations (eTables 21 and 22 and eAppendix in the Supplement).

## Discussion

This nationwide retrospective cohort study of US veteran patients with documented SARS-CoV-2 infection after vaccination identified clinical and demographic variables associated with risk of severe disease, defined as either death within 28 days or hospitalization with evidence of respiratory failure or hypoxemia. We took a high-altitude approach to a broad range of possible risk factors in a large population, rather than attempting to dissect the details of individual comorbidities, immunocompromised states, or factors specific to vaccine products and variants. Risk was reduced among booster vaccine recipients and those with a history of infection before vaccination and was lower during the Omicron period. Increasing age had the most substantial association with risk, which increased steadily at least among patients older than 50 years, but a large number of comorbidities remained associated with risk of severe disease in the adjusted model. In light of high rates of vaccination and prior infection, research on these details in breakthrough infection and reinfection, focused on severity and sequelae, has the most potential to inform clinical practice through risk-stratification of individual patients.^10^ Recent data suggest that low-risk vaccinated patients receive little or no benefit from antiviral therapies, and thus improving identification of patients at high risk for progression is important for informing clinical practice and directing distribution of antivirals to those most likely to benefit.^11^

Immunization during periods of immunocompromise has been theorized to negatively affect vaccine response rates and predispose to risk of infection and severe outcomes. In this large cohort, an immunocompromised state conferred increased risk of severe outcomes; the association between the immunosuppressive drugs and risk was stronger if present after vaccination (ie, producing an immunocompromised state at the time of exposure and subsequent infection) than if present only prior to vaccination. These data suggest that many patients receiving immunosuppressive medications at the time of vaccine develop durable protection, and the causal risk factor is immune status at the time of exposure.

The comorbidities most strongly associated with odds of severe disease include those identified early in the pandemic as the key risk factors for severe disease before vaccines were available.^12,13,14^ Comorbidities indicating preexisting organ disease (eg, heart failure, chronic kidney disease, COPD, dementia) or a globally tenuous or frail state (eg, pressure ulcers, mobility impairments, low BMI) had stronger associations with risk than factors that contribute to future organ dysfunction (eg, hypertension). However, age was so much stronger as a risk factor for severe outcomes that the magnitudes of risk associated with any source of immunocompromise, or the most important comorbidities, were similar to the difference in risk between vaccinated persons aged 60 vs 50 years. We cannot explain the apparent higher risk among the small number (890) of Native American individuals in the cohort, but the similar risks among White, Black and African American, and Hispanic and Latino groups likely reflects the VA providing similar access in most regions.^15^

We also identified factors that were protective against severe outcomes; consistent with prior studies, boosters or a history of infection prior to initial vaccination significantly reduced (to a similar degree) but did not eliminate risk of severe breakthrough in all subcohorts. Although increasing time since initial vaccination appeared to be associated with severe outcomes in the unstratified analyses, different effect estimates in the subcohorts suggested residual confounding. Among nonimmunocompromised patients who did not receive boosters (at low risk or moderate to high risk depending on age) no reduction in risk of severe disease during the limited follow-up period was observed, although boosting did provide additional protection against severe disease. This study does not provide insight into optimal timing for repeated boosting or if repeated boosting is necessary for all individuals, as only 5 months of data on risk were available after boosters were recommended for nonimmunocompromised patients with other risk factors and even shorter follow-up was available for the general population.

Few studies have addressed the severity of breakthrough infection. Inclusion of objective metrics to assess severity^5,6^ is a strength compared with studies that do not account for in-hospital screening practices that affect the hospitalization plus a positive test definition used by COVID-NET.^16^ Limitation to patients with documented vaccination and documented breakthrough infection avoids bigger problems with missing data and unmeasurable confounders that can affect population-based studies trying to compare patients who are vaccinated with infection (our cohort) with either patients unvaccinated with infection or patients vaccinated without infection, including test-negative designs.^17^ The CDC published a report of 189 severe cases among 2246 breakthrough infections and found that risk factors for hospitalization and death among vaccinated patients included age 65 years or older, immunocompromised status, and heart, liver, kidney, neurologic disease and diabetes; all patients who died had multiple comorbidities.^18^ Most studies of patients in immunocompromised states have focused on laboratory-based studies rather than clinical outcomes.^19^ A recent study of breakthrough infection in immunocompromised patients focused on comparing the conditions causing immune dysfunction and did not attempt to determine risk factors for severity.^20^ Multiple studies have shown decline in protection after initial vaccination in the general population, with some incorporating severity^3,21,22^ and others showing improvement after boosting.^1,2^

### Limitations

This study has important limitations. As with all VA studies, the population was predominantly male and older (mean age 62 years, and >70 years among patients with severe disease) and had a high burden of chronic medical problems. The study is not generalizable to female patients nor to younger patients with substantial comorbidities, although in our subcohort analyses, we did not find significant differences in risk factors in these groups. Our use of a nationwide database curated from electronic health records produces additional limitations: uncertainty about the accuracy of COVID-19 as the reason for hospitalization, imperfect algorithms for comorbidities, and missing data, most likely regarding prior infection, boosters, hospitalization at facilities not contracted for reimbursement by the VA, and use of monoclonal antibodies. To address these limitations, we focused on questions that would not depend on data that might be missing. Missing data that could affect our results include boosting, prior infection, and admission to some non-VA hospitals in the outpatient group. Misclassification for boosting or prior infection would attenuate their benefit, which continued to be apparent in our analyses. Misclassification on the basis of inpatient utilization could magnify the effect of any variable more common among patients classified as having severe infection. However, the predominance of the Omicron variant and the relatively high rate of boosting—both widely accepted as being associated with lower risk of severe disease—in the outpatient group argue that this younger, healthier group of patients does not include large numbers of severely ill patients who sought non-VA care more than older patients with more comorbidities. The use of a cohort rather than a matched case-control design, which has been commonly used in studies to estimate vaccine effectiveness, with 90% of patients having nonsevere disease, is less likely to pick up uncommon risk factors and may be more susceptible to residual confounding considering differences such as younger age and fewer comorbidities. Our prespecified analysis of patients with immunocompromising conditions addresses a group that has been widely discussed but understudied with regard to clinical protection via vaccination.^19,20,23^ A limitation in that analysis is that these drugs are highly diverse, which necessitated grouping drugs with very different mechanisms of action, eg, into leukocyte-inhibiting or cytokine-blocking drugs, and more granular analysis is needed to further risk stratify these patients. In addition, our ability to assess the association of immunosuppressive medications with risk of severe disease was limited by our inability to identify systemic use of certain medications (eg, tacrolimus) and inability to determine glucocorticoid doses. With the caveat of these limitations, the accuracy of VA data on medication dispensing and our ability to evaluate the association between timing of medication receipt and timing of vaccination is a major strength of this study.

## Conclusions

This study provides insight into the demographic, clinical, and vaccination-related risk factors for severe compared with nonsevere breakthrough SARS-CoV-2 infection in a predominantly male cohort. These results could be used to bolster guidelines for administration of preexposure prophylaxis and to identify patients most likely to benefit from antiviral therapy. Development of models to estimate the probability of a patient progressing to severe disease for individual risk assessment and to guide treatment and prophylaxis planning and outreach will require more sophisticated approaches, such as machine-learning models to continue to inform best clinical practices for COVID-19 management.

## Notes

Supplement. **eMethods.** Methods With Additional Details **eTable 1.** Data Dictionary **eTable 2.** List of Immunosuppressive Medications, With Classification **eTable 3.** Full Cohort, Data and Analysis, Univariable and Multivariable **eTable 4.** Full Cohort Data, 4 Levels of Severity **eTable 5.** Subcohorts, Data for Patients Younger and Older Than 50 Years, Analysis for Patients Younger Than 50 Years **eAppendix.** Additional Results **eTable 6.** Subcohort Immunocompromised, Data and Analysis **eTable 7.** Immunosuppressive Medications Before and/or After Vaccination **eTable 8.** Subcohort of Immunocompromised Patients, Analysis by Number of Medication Classes **eTable 9.** Subcohort of Nonimmunocompromised Patients, Data and Analysis **eTable 10.** Subcohort of Patients Aged 50 or Older, Data and Analysis **eTable 11.** Subcohort of Nonimmunocompromised Patients Younger Than 65 Years With No Booster, Data and Analysis **eTable 12.** Subcohort of Nonimmunocompromised Patients Aged 65 Years or Older With No Booster, Data and Analysis **eFigure.** Age Distributions of Male and Female Patients **eTable 13.** Subcohort of Female Patients, Data and Analysis **eTable 14.** Subcohort of Male Patients, Data and Analysis **eTable 15.** Subcohorts of Female and Male, Comparison of Analyses **eTable 16.** Subcohort During Delta Period, Data and Analysis **eTable 17.** Subcohort During Omicron Period, Data and Analysis **eTable 18.** Subcohorts During Delta and Omicron, Comparison of Analyses **eTable 19.** Subcohort of Patients Who Received the Pfizer Vaccine, Data and Analysis **eTable 20.** Subcohort of Patients Who Received the Moderna Vaccine, Data and Analysis **eTable 21.** Subcohort of Patients With Rural Residence, Data and Analysis **eTable 22.** Subcohort of Patients With Urban Residence, Data and Analysis
