# Errors in Methods Section and Supplement

**DOI:** 10.1001/jamanetworkopen.2023.1692

**Published:** 2023-02-13

In the Original Investigation titled “Factors Associated With Severe COVID-19 Among Vaccinated Adults Treated in US Veterans Affairs Hospitals,”^1^ published October 20, 2022, there were errors in the Methods section and online-only Supplement. The period of data collection for comorbidities started December 15, 2020, and concluded at the time of vaccination. This article has been corrected.^1^
