# Supplementary material for: Factors Associated With Severe COVID-19 Among Vaccinated Adults Treated in US Veterans Affairs Hospitals
Source: JAMA Netw Open. 2022 Oct 20;5(10):e2240037. doi: 10.1001/jamanetworkopen.2022.40037 (PMC9585432; doi:10.1001/jamanetworkopen.2022.40037)
Supplement: Supplement. — eMethods. Methods With Additional Details eTable 1. Data Dictionary eTable 2. List of Immunosuppressive Medications, With Classification eTable 3. Full Cohort, Data and Analysis, Univariable and Multivariable eTable 4. Full Cohort Data, 4 Levels of Severity eTable 5. Subcohorts, Data for Patients Younger and Older Than 50 Years, Analysis for Patients Younger Than 50 Years eAppendix. Additional Results eTable 6. Subcohort Immunocompromised, Data and Analysis eTable 7. Immunosuppressive Medications Before and/or After Vaccination eTable 8. Subcohort of Immunocompromised Patients, Analysis by Number of Medication Classes eTable 9. Subcohort of Nonimmunocompromised Patients, Data and Analysis eTable 10. Subcohort of Patients Aged 50 or Older, Data and Analysis eTable 11. Subcohort of Nonimmunocompromised Patients Younger Than 65 Years With No Booster, Data and Analysis eTable 12. Subcohort of Nonimmunocompromised Patients Aged 65 Years or Older With No Booster, Data and Analysis eFigure. Age Distributions of Male and Female Patients eTable 13. Subcohort of Female Patients, Data and Analysis eTable 14. Subcohort of Male Patients, Data and Analysis eTable 15. Subcohorts of Female and Male, Comparison of Analyses eTable 16. Subcohort During Delta Period, Data and Analysis eTable 17. Subcohort During Omicron Period, Data and Analysis eTable 18. Subcohorts During Delta and Omicron, Comparison of Analyses eTable 19. Subcohort of Patients Who Received the Pfizer Vaccine, Data and Analysis eTable 20. Subcohort of Patients Who Received the Moderna Vaccine, Data and Analysis eTable 21. Subcohort of Patients With Rural Residence, Data and Analysis eTable 22. Subcohort of Patients With Urban Residence, Data and Analysis [file jamanetwopen-e2240037-s001.pdf]

Supplemental Online Content

Vo AD, La J, Wu JTY, et al. Factors associated with severe COVID-19 among vaccinated adults treated in US Veterans Affairs hospitals. *JAMA Netw Open.* 2022;5(10):e2240037. doi:10.1001/jamanetworkopen.2022.40037

eMethods. Methods With Additional Details

eTable 1. Data Dictionary

eTable 2. List of Immunosuppressive Medications, With Classification

eTable 3. Full Cohort, Data and Analysis, Univariable and Multivariable

eTable 4. Full Cohort Data, 4 Levels of Severity

eTable 5. Subcohorts, Data for Patients Younger and Older Than 50 Years, Analysis for Patients Younger Than 50 Years

eAppendix. Additional Results

eTable 6. Subcohort Immunocompromised, Data and Analysis

eTable 7. Immunosuppressive Medications Before and/or After Vaccination

eTable 8. Subcohort of Immunocompromised Patients, Analysis by Number of Medication Classes

eTable 9. Subcohort of Nonimmunocompromised Patients, Data and Analysis

eTable 10. Subcohort of Patients Aged 50 or Older, Data and Analysis

eTable 11. Subcohort of Nonimmunocompromised Patients Younger Than 65 Years With No Booster, Data and Analysis

eTable 12. Subcohort of Nonimmunocompromised Patients Aged 65 Years or Older With No Booster, Data and Analysis

eFigure. Age Distributions of Male and Female Patients

eTable 13. Subcohort of Female Patients, Data and Analysis

eTable 14. Subcohort of Male Patients, Data and Analysis

eTable 15. Subcohorts of Female and Male, Comparison of Analyses

eTable 16. Subcohort During Delta Period, Data and Analysis

eTable 17. Subcohort During Omicron Period, Data and Analysis

eTable 18. Subcohorts During Delta and Omicron, Comparison of Analyses

eTable 19. Subcohort of Patients Who Received the Pfizer Vaccine, Data and Analysis

eTable 20. Subcohort of Patients Who Received the Moderna Vaccine, Data and Analysis

eTable 21. Subcohort of Patients With Rural Residence, Data and Analysis

eTable 22. Subcohort of Patients With Urban Residence, Data and Analysis

© 2022 Vo AD et al. *JAMA Network Open.*

This supplemental material has been provided by the authors to give readers additional information about their work.

© 2022 Vo AD et al. *JAMA Network Open.*

eMethods. Methods With Additional Details

Cohort creation:

The longitudinal cohort consisted of all patients who received an initial 2-dose series of mRNA COVID-19 vaccine (BNT162b2 or mRNA-1273) or a single dose of the Janssen adenovirus-based vaccine (Ad26.COV2.S) before 12/1/2021 and subsequently tested positive for SARS-CoV-2 infection (PCR or antigen) within the Veterans Affairs Healthcare System (VA) through 2/28/2022. The study period includes intervals during which the original strain, delta variant (7/1/2021-12/15/2021), and omicron variant 12/16/2021-2/28/2021) were predominant.

"Fully vaccinated" with the primary vaccination series was defined as 14 days after the second dose of an mRNA vaccine or 14 days after a single dose of the Janssen vaccine. "Boosted" was defined as 7 days after an additional vaccine dose of any type. SARS-CoV-2 infection was defined by a positive PCR or antigen test at a VA facility. Only the first breakthrough infection was included, and the first positive test after vaccination was used to define the start time of that breakthrough infection.

Data sources and uses:

Data were obtained from the VA COVID-19 shared data resource[4] and the Corporate Data Warehouse (CDW) that encompasses the clinical electronic health records (EHR) of all VA patients and facilities. See **eTable 1** for a complete listing of definitions and data sources.

The primary outcome was severe breakthrough infection, defined by either: death 2-28 days after a positive test; medical/surgical hospitalization within 14 days of the positive test and documented SpO2 < 94%, receipt of any supplemental oxygen, or receipt of dexamethasone; or mechanical ventilation. The comparator was non-severe breakthrough infection: no hospitalization, or hospitalization that did not meet criteria for severe disease.

For descriptive purposes, a total of 4 categories of severity were defined (non-severe disease and not hospitalized, non-severe but hospitalized, severe and survived, or died within 28 days of the first positive test), but these were not analyzed separately (see **eTable 4**). Admissions to psychiatric units, a VA community living center, or a VA domiciliary were not included in the hospitalization definition, nor were admissions to acute care facilities lasting less than 1 day; all such patients were classified as having not been hospitalized, i.e. as having non-severe breakthrough infection. A random sample of 25 patients who died underwent manual chart review to determine whether death was attributable to COVID-19 (see **eResults**). Patients who were hospitalized and survived but for whom data about severity could not be determined were excluded.

Demographic data included age in years at the time of breakthrough infection, biologic sex, self-reported race and ethnicity, US region, and urban/rural residence as defined by US Census criteria. Age was classified as < 40, ≥40 - <45, ≥45 - <50 (referent group), ≥50 - < 55, ≥55 - <60, ≥60 - <65, ≥65 - <70, ≥70 - <75, ≥75 - <80, and ≥80. Vaccination data included dates of each vaccination dose and the vaccine manufacturer. Occurrence of a previous SARS-CoV-2 infection before vaccination was defined as a positive test any time before day 14 after completing the initial vaccination series. Time from receipt of vaccination was assessed as a categorical variable defined as months since vaccination or receipt of booster.

© 2022 Vo AD et al. *JAMA Network Open.*

Occurrence of a previous SARS-CoV-2 infection before vaccination was defined as a positive test any time before day 14 after completing the initial vaccination series. Use of monoclonal antibody therapy to prevent severe Covid-19 after breakthrough infection was recorded as a dichotomous variable.

Comorbidities recorded in the patients' records at the time of infection were assessed using the Chronic Conditions Warehouse (CCW) during the 3 years prior to vaccination. Some comorbidities in the CCW were excluded from the multivariable modeling on the basis of being deemed (by PM and WBE) as redundant, or very unlikely to be related to Covid-19 outcomes, or present at very low frequencies.

Body mass index (BMI) in kg/m^2 was calculated from weight and height and was classified into underweight (BMI < 18.5), normal weight (BMI ≥18 - < 25, referent group), overweight (≥25 - < 30), and obesity grade I (≥30 - < 35), II (≥35 - < 40), and severe (≥ 40); in the approximately 30% of patients for whom height was not available, the US averages for men (1.78 m) and women (1.63 m) were used to impute BMI.

Definitions of immunocompromised patients:

Medication administration data within the VA were reviewed, and medications known to increase risk of infectious diseases were classified into five categories: cytotoxic chemotherapy within 6 months, glucocorticoids (oral prednisone or oral or IV methylprednisolone) at any dose within 1 month, cytokine-blocking drugs (e.g., anti-TNF, -IL-6R, -IL-17), within 3 months, leukocyte-inhibiting drugs (e.g. methotrexate, azathioprine, JAK inhibitors, abatacept) within 3 months, and lymphocyte-depleting drugs (e.g. rituximab) within 18 months. Some potentially immunosuppressive medications were not included in the definition due to challenges distinguishing systemic from topical formulations (e.g., cyclosporine, tacrolimus, and multiple topical and inhaled glucocorticoids). Use of immunosuppressive drugs was assessed separately both shortly before vaccination and after vaccination but shortly before breakthrough infection. Different time intervals were used for different drug classes and based on their expected duration of impact and maximum length of a single prescription for outpatient drugs. A full list of drugs, their classifications, timing used to define recent use, and the numbers of patients receiving these drugs before vaccination and after vaccination (i.e., before breakthrough) are presented in **eTable 2**. The immunocompromised cohort also included patients with leukemia or lymphoma but not patients with HIV, based on the results of the analysis of the full cohort.

Time since Vaccination

Interpreting time-since-vaccination has risk for residual confounding, due to earlier availability to higher-risk patients, differential use and timing of boosting, and development of the delta and then the less-dangerous omicron variant. Thus, in addition to including time-since-initial-vaccination and time-since-boosting in the same multivariable models, we also analyzed non-immunocompromised patients who had not received boosters and stratified by age less than or greater than 65. The under-65 group was further limited to patients initially vaccinated after March 29, 2021, when vaccination was recommended for all US adults independent of health status.

Statistical Analysis:

The primary analysis evaluated exposures associated with development of severe compared to non-severe SARS-CoV-2 infection despite vaccination. Secondary analyses were conducted on subcohorts (**Figure 1**), to further explore the impacts of an immunocompromised state, age, and time since vaccination. Analyses stratified by sex, viral variant predominance, vaccine manufacturer, and urban or rural residence were also performed

Multivariable logistic regression was used to assess the association of the binary outcome (severe vs non-severe breakthrough SARS-CoV-2 infection) with sets of independent variables, which were modeled either as binary or in categories (e.g. for geographic region) with one chosen as the referent. For some independent variables, missing data was treated by including an "unknown" category, so as to not need to exclude the patient from the analysis. For comorbidities, missing data was regarded as absence of the condition. Variables determined *a priori* to be of interest and those with adjusted odds ratios (aOR) ≥ 1.5 or ≤ 0.67 and statistical significance are presented in the main results. Results for all variables included in each multivariable are included in eTables, and it is apparent in those tables when a set of variables was excluded from an analysis. Findings were considered statistically significant at $P<0.05$ after adjustment for multiple comparisons using the Benjamini-Hochberg method, using the ranked P-values of that multivariable regression. The resulting cut-offs for actual P-values are reported below each of the eTables.

Ethical review of study

This study was approved by the VA Boston Research and Development committee as an exempt study prior to data collection and analysis with a waiver of informed consent.

© 2022 Vo AD et al. *JAMA Network Open.*

STROBE Statement—Checklist of items that should be included in reports of ***cohort studies***

	Item No	Recommendation	Page No (submitted manuscript)
**Title and abstract**	1	(*a*) Indicate the study's design with a commonly used term in the title or the abstract	
		(*b*) Provide in the abstract an informative and balanced summary of what was done and what was found	3-4
**Introduction**			
Background/rationale	2	Explain the scientific background and rationale for the investigation being reported	5
Objectives	3	State specific objectives, including any prespecified hypotheses	5
**Methods**			
Study design	4	Present key elements of study design early in the paper	5-8
Setting	5	Describe the setting, locations, and relevant dates, including periods of recruitment, exposure, follow-up, and data collection	5-6
Participants	6	(*a*) Give the eligibility criteria, and the sources and methods of selection of participants. Describe methods of follow-up	5-6
		(*b*) For matched studies, give matching criteria and number of exposed and unexposed (N/A)	
Variables	7	Clearly define all outcomes, exposures, predictors, potential confounders, and effect modifiers. Give diagnostic criteria, if applicable	6-7
Data sources/ measurement	8*	For each variable of interest, give sources of data and details of methods of assessment (measurement). Describe comparability of assessment methods if there is more than one group	5-6, eMethods
Bias	9	Describe any efforts to address potential sources of bias (in Limitations)	13-14
Study size	10	Explain how the study size was arrived at (all available data were used)	5-6
Quantitative variables	11	Explain how quantitative variables were handled in the analyses. If applicable, describe which groupings were chosen and why	5-6
Statistical methods	12	(*a*) Describe all statistical methods, including those used to control for confounding	7-8
		(*b*) Describe any methods used to examine subgroups and interactions (same)	7-8
		(*c*) Explain how missing data were addressed (only done for BMI)	6
		(*d*) If applicable, explain how loss to follow-up was addressed	N/A

© 2022 Vo AD et al. *JAMA Network Open.*

		(*e*) Describe any sensitivity analyses (subgroups in results, same methods)	N/A
**Results**			
Participants	13*	(a) Report numbers of individuals at each stage of study—eg numbers potentially eligible, examined for eligibility, confirmed eligible, included in the study, completing follow-up, and analysed (report number eligible = included)	8
		(b) Give reasons for non-participation at each stage (reasons for ineligibility are complex and not of scientific interest)	N/A
		(c) Consider use of a flow diagram (used to describe subsets)	Fig1
Descriptive data	14*	(a) Give characteristics of study participants (eg demographic, clinical, social) and information on exposures and potential confounders	8-9, eTable3
		(b) Indicate number of participants with missing data for each variable of interest	eTable4
		(c) Summarise follow-up time (eg, average and total amount)	5-6
Outcome data	15*	Report numbers of outcome events or summary measures over time	eTable3+4

Main results	16	(*a*) Give unadjusted estimates and, if applicable, confounder-adjusted estimates and their precision (eg, 95% confidence interval). Make clear which confounders were adjusted for and why they were included	Table1<br>eTable3
		(*b*) Report category boundaries when continuous variables were categorized	eTables
		(*c*) If relevant, consider translating estimates of relative risk into absolute risk for a meaningful time period	8-10<br>eTables
Other analyses	17	Report other analyses done—eg analyses of subgroups and interactions, and sensitivity analyses	9-10<br>eTables
**Discussion**			
Key results	18	Summarise key results with reference to study objectives	11-12
Limitations	19	Discuss limitations of the study, taking into account sources of potential bias or imprecision. Discuss both direction and magnitude of any potential bias	14-15
Interpretation	20	Give a cautious overall interpretation of results considering objectives, limitations, multiplicity of analyses, results from similar studies, and other relevant evidence	11-15

© 2022 Vo AD et al. *JAMA Network Open.*

Generalisability	21	Discuss the generalisability (external validity) of the study results	13-15
**Other information**			
Funding	22	Give the source of funding and the role of the funders for the present study and, if applicable, for the original study on which the present article is based	16

*Give information separately for exposed and unexposed groups.

Note: An Explanation and Elaboration article discusses each checklist item and gives methodological background and published examples of transparent reporting. The STROBE checklist is best used in conjunction with this article (freely available on the Web sites of PLoS Medicine at http://www.plosmedicine.org/, Annals of Internal Medicine at http://www.annals.org/, and Epidemiology at http://www.epidem.com/). Information on the STROBE Initiative is available at http://www.strobe-statement.org.

© 2022 Vo AD et al. *JAMA Network Open.*

eTable 1. Data Dictionary

Variable	Definition
Breakthrough infection	Positive SARS-CoV-2 test at least 14 days after the second dose of mRNA vaccine or first dose of adenoviral vaccine. Only the first breakthrough infection was analyzed
Non-severe	Severity 1 or 2
Severe	Severity 3, 4, or 5
Sex	As recorded in structured data in the VA EHR
Race	As recorded in structured data in the VA EHR
Ethnicity	As recorded in structured data in the VA EHR
Age	In years at time of breakthrough infection
Age ranges	
Less than 40	Age < 40
40-45	Age ≥ 40 AND Age < 45
45-50	Age ≥ 45 AND Age < 50
50-55	Age ≥ 50 AND Age < 55
55-60	Age ≥ 55 AND Age < 60
60-65	Age ≥ 60 AND Age < 65
65-70	Age ≥ 65 AND Age < 70
70-75	Age ≥ 70 AND Age < 75
75-80	Age ≥ 75 AND Age < 80
80 or greater	Age ≥ 80
BMI	Calculated using most recent weight in kg within 5 years, and most recent height ever recorded. If height missing, then used US averages of 1.78m for males and 1.63m for females
BMI Class	
Underweight	BMI < 18.5
Normal	BMI ≥ 18.5 AND BMI < 25
Overweight	BMI ≥ 25 AND BMI < 30
Obesity I	BMI ≥ 30 AND BMI < 35
Obesity II	BMI ≥ 35 AND BMI < 40
Severe Obesity	BMI ≥ 40
Unknown	Weight or height unavailable
Region	Geographical region was defined based on the VA facility from which each patient received their full vaccination (second dose of mRNA vaccine or first dose of adenoviral vaccine)
Continental	Arkansas, Colorado, Louisiana, Mississippi, Montana, Oklahoma, Texas, Utah, Wyoming

© 2022 Vo AD et al. *JAMA Network Open.*

Midwest	Illinois, Indiana, Michigan, Minnesota, Missouri, Nebraska, North Dakota, Ohio, South Dakota, Wisconsin
North Atlantic	Connecticut, Delaware, District Of Columbia, Maine, Maryland, Massachusetts, New Hampshire, New Jersey, New York, North Carolina, Pennsylvania, Rhode Island, Vermont, Virginia, West Virginia
Pacific	Alaska, Arizona, California, Hawaii, Idaho, Nevada, New Mexico, Oregon, Washington
Southeast	Alabama, Florida, Georgia, Kentucky, Puerto Rico, South Carolina, Tennessee
Unknown	Data on the VA facility not available
Urban or rural residence	The most recent residence listed in VA records was used. For patients with multiple residences, the one closest to the vaccination site was used. Locations were defined as urban per US Census criteria, and all others were considered rural. VA also defines census regions as highly rural if they contain fewer than 7 residents per square mile. However, since only 0.4% of patients in this study resided in highly rural areas, they were combined with the rural group for analysis.
Vaccine Type	Defined based on the type of the initial vaccination, as recorded in the VA COVID-19 Shared Data Resource. (Patients with first and second doses of different types were excluded.)
Janssen	Patient considered vaccinated 14 days after 1st dose
Moderna	Patient considered vaccinated 14 days after 2nd dose
Pfizer	Patient considered vaccinated 14 days after 2nd dose
Months since full vax at breakthrough	Days since full vax converted to months and discretized into multiple buckets of 1 month durations
4 or less	Months ≤ 4
4-5	4 < Months AND Months ≤ 5
5-6	5 < Months AND Months ≤ 6
6-7	6 < Months AND Months ≤ 7
7-8	7 < Months AND Months ≤ 8
8-9	8 < Months AND Months ≤ 9
9-10	9 < Months AND Months ≤ 10
10-11	10 < Months AND Months ≤ 11
11-12	11 < Months AND Months ≤ 12
12 or more	12 < Months
Boosted before breakthrough	Patients are considered fully boosted 7 days after receiving the booster shot. Thus, the patient was considered boosted before breakthrough if the breakthrough infection occurred at least 7 days after receiving the booster shot
Not boosted	
1 or less	Months ≤ 1

© 2022 Vo AD et al. *JAMA Network Open.*

1-2	1 < Months AND Months ≤ 2
2-3	2 < Months AND Months ≤ 3
3-4	3 < Months AND Months ≤ 4
4-5	4 < Months AND Months ≤ 5
5-6	5 < Months AND Months ≤ 6
6 or more	6 < Months
History of infection before vaccinated	Previous positive SARS-CoV-2 test (PCR or antigen) no later than 14 days after the second mRNA vaccine or first adenoviral vaccine. No time limit was applied, meaning any VA-diagnosed case from early 2020 should be captured.
Delta Period	Between 7/1/2021 and 12/15/2021, inclusive
Omicron Period	Between 12/16/2021 and 2/28/2022, inclusive
Persistent positive test	Patients whose first breakthrough infection was persistent enough to last longer than the standard 6-week infection duration. Starting from the date of the first breakthrough infection, we required at least two additional positive tests in the subsequent 12 weeks, with at least one of them falling in the first 6 weeks and at least one falling in the second 6 weeks, and no negative tests in these 12 weeks
Immune-suppressive medications before breakthrough	
Before breakthrough Chemotherapy	Order/dispensation of one of the drugs listed as chemotherapy in eTable 2 within 6 months before date of breakthrough infection
Before breakthrough Cytokine-blocking	Order/dispensation of one of the drugs listed as cytokine-blocking in eTable 2 within 3 months before date of breakthrough infection
Before breakthrough Glucocorticoids	Order/dispensation of one of the drugs listed as cytokine-blocking in eTable 2 within 1 month before date of breakthrough infection
Before breakthrough Leukocyte-inhibitory	Order/dispensation of one of the drugs listed as cytokine-blocking in eTable 2 within 3 months before date of breakthrough infection
Before breakthrough Lymphocyte-depleting	Order/dispensation of one of the drugs listed as cytokine-blocking in eTable 2 within 18 months before date of breakthrough infection
Immune-suppressive medications before initial vaccination	
Before vaccine Chemotherapy	Order/dispensation of one of the drugs listed as chemotherapy in eTable 2 within 6 months before receipt of the first vaccine

© 2022 Vo AD et al. *JAMA Network Open.*

Before vaccine Cytokine-blocking	Order/dispensation of one of the drugs listed as cytokine-blocking in eTable 2 within 3 months before receipt of the first vaccine
Before vaccine Glucocorticoids	Order/dispensation of one of the drugs listed as cytokine-blocking in eTable 2 within 1 month before receipt of the first vaccine
Before vaccine Leukocyte-inhibitory	Order/dispensation of one of the drugs listed as cytokine-blocking in eTable 2 within 3 months before receipt of the first vaccine
Before vaccine Lymphocyte-depleting	Order/dispensation of one of the drugs listed as cytokine-blocking in eTable 2 within 18 months before receipt of the first vaccine
Monoclonal Antibody	Order/dispensation of one of the drugs listed as MAB in eTable 2 given at any time
Comorbidities	These comorbidities were derived based the diagnosis and procedure codes described in the CMS Chronic Conditions Warehouse algorithms (https://www2.ccwdata.org/web/guest/condition-categories) anytime in the 3 years prior to the date of full vaccination
Acquired hypothyroidism	
Acute myocardial infarction	
Alzheimers disease and related disorders or senile dementia	
Anemia	
Anxiety disorders	
Asthma	
Atrial fibrillation	
Bipolar disorder	
Chronic kidney disease	
Chronic obstructive pulmonary disease and bronchiectasis	
Colorectal cancer	
Depression	
Diabetes	
Epilepsy	
Heart failure	
Human immunodeficiency virus and/or acquired immunodeficiency syndrome HIV/AIDS	
Hyperlipidemia	
Hypertension	

© 2022 Vo AD et al. *JAMA Network Open.*

Ischemic heart disease	
Leukemias and lymphomas	
Liver disease cirrhosis and other liver conditions except viral hepatitis	
Lung cancer	
Mobility impairments	
Multiple sclerosis and transverse myelitis	
Peripheral vascular disease	
Pressure and chronic ulcers	
Prostate cancer	
Schizophrenia and other psychotic disorders	
Tobacco use	
Viral hepatitis	
COVID19_ICD used	Use of ICD-10 code U07.1 or J12.82 during an inpatient visit
Inpatient Location	Location of inpatient visits derived from VistA's Patient Treatment File (PTF)
VA only	VA acute care sites, excluding psychiatric, domiciliary, rehabilitation, or community living center (CLC) sites
VA and non-VA	Inpatient visits to both VA and non-VA care sites
Non-VA only	Patients receiving care from a non-VA hospital under VA auspices
Unknown	Data about location of care site was not found

© 2022 Vo AD et al. *JAMA Network Open.*

eTable 2. List of Immunosuppressive Medications, With Classification

Drug class	Months[a]	Generic drug name	Patients receiving before vaccination	Patients receiving after vaccination and before breakthrough
Chemotherapy	6	arsenic	6	12
Chemotherapy	6	asparaginase	0	0
Chemotherapy	6	asparaginase erwinia chrysanthemi	0	0
Chemotherapy	6	azacitidine	16	31
Chemotherapy	6	bendamustine	21	35
Chemotherapy	6	bleomycin	7	4
Chemotherapy	6	busulfan	0	0
Chemotherapy	6	cabazitaxel	2	10
Chemotherapy	6	calaspargase	0	0
Chemotherapy	6	capecitabine	31	69
Chemotherapy	6	carboplatin	49	171
Chemotherapy	6	carmustine	0	1
Chemotherapy	6	chlorambucil	3	3
Chemotherapy	6	cisplatin	14	69
Chemotherapy	6	cladribine	1	1
Chemotherapy	6	clofarabine	0	0
Chemotherapy	6	cyclophosphamide	37	99
Chemotherapy	6	cytarabine	2	8
Chemotherapy	6	dacarbazine	8	7
Chemotherapy	6	dactinomycin	0	0
Chemotherapy	6	daunorubicin	0	1
Chemotherapy	6	decitabine	10	27
Chemotherapy	6	docetaxel	21	66
Chemotherapy	6	doxorubicin	35	105
Chemotherapy	6	epirubicin	2	8
Chemotherapy	6	eribulin	0	0
Chemotherapy	6	etoposide	10	60
Chemotherapy	6	fludarabine	2	1
Chemotherapy	6	gemcitabine	38	126
Chemotherapy	6	hydroxycamptothecin	0	0
Chemotherapy	6	hydroxyurea	90	146
Chemotherapy	6	idarubicin	0	0
Chemotherapy	6	ifosfamide	0	2
Chemotherapy	6	irinotecan	19	47

© 2022 Vo AD et al. *JAMA Network Open.*

Chemotherapy	6	ixabepilone	0	0
Chemotherapy	6	lomustine	0	1
Chemotherapy	6	mechlorethamine	0	1
Chemotherapy	6	melphalan	0	2
Chemotherapy	6	mercaptopurine	28	41
Chemotherapy	6	mitomycin c	0	0
Chemotherapy	6	mitoxantrone	0	1
Chemotherapy	6	nelarabine	0	0
Chemotherapy	6	oxaliplatin	33	91
Chemotherapy	6	paclitaxel	46	141
Chemotherapy	6	pegaspargase	0	1
Chemotherapy	6	pemetrexed	18	50
Chemotherapy	6	pralatrexate	0	0
Chemotherapy	6	procarbazine	0	1
Chemotherapy	6	temozolomide	7	7
Chemotherapy	6	thioguanine	0	1
Chemotherapy	6	thiotepa	0	1
Chemotherapy	6	topotecan	0	1
Chemotherapy	6	trabectedin	0	1
Chemotherapy	6	trifluridine	4	16
Chemotherapy	6	valrubicin	0	1
Chemotherapy	6	vinblastine	8	7
Chemotherapy	6	vincristine	13	52
Chemotherapy	6	vindesine	0	0
Chemotherapy	6	vinorelbine	0	2
Cytokine-blocking	3	adalimumab	532	630
Cytokine-blocking	3	anakinra	6	7
Cytokine-blocking	3	benralizumab	32	59
Cytokine-blocking	3	brodalumab	0	0
Cytokine-blocking	3	canakinumab	0	0
Cytokine-blocking	3	certolizumab	36	46
Cytokine-blocking	3	dupilumab	95	148
Cytokine-blocking	3	etanercept	188	203
Cytokine-blocking	3	golimumab	11	13
Cytokine-blocking	3	infliximab	102	151
Cytokine-blocking	3	ixekizumab	46	88
Cytokine-blocking	3	mepolizumab	56	64
Cytokine-blocking	3	rilonacept	0	0

© 2022 Vo AD et al. *JAMA Network Open.*

Cytokine-blocking	3	sarilumab	0	1
Cytokine-blocking	3	secukinumab	59	60
Cytokine-blocking	3	tocilizumab	21	88
Cytokine-blocking	3	ustekinumab	60	80
Glucocorticoids	1	methylprednisolone	1026	3760
Glucocorticoids	1	prednisone	2009	4675
Leukocyte-inhibitory	3	abatacept	41	57
Leukocyte-inhibitory	3	azathioprine	177	210
Leukocyte-inhibitory	3	baricitinib	1	73
Leukocyte-inhibitory	3	belimumab	5	5
Leukocyte-inhibitory	3	dimethyl fumarate	0	0
Leukocyte-inhibitory	3	fingolimod	19	20
Leukocyte-inhibitory	3	leflunomide	129	168
Leukocyte-inhibitory	3	methotrexate	629	789
Leukocyte-inhibitory	3	mycophenolate	229	389
Leukocyte-inhibitory	3	mycophenolic	54	119
Leukocyte-inhibitory	3	natalizumab	3	7
Leukocyte-inhibitory	3	rapamycin	4	9
Leukocyte-inhibitory	3	sirolimus	42	67
Leukocyte-inhibitory	3	teriflunomide	14	12
Leukocyte-inhibitory	3	tofacitinib	71	84
Leukocyte-inhibitory	3	upadacitinib	1	5
Leukocyte-inhibitory	3	vedolizumab	41	54
Lymphocyte-depleting	18	alemtuzumab	1	6
Lymphocyte-depleting	18	obinutuzumab	13	27
Lymphocyte-depleting	18	ocrelizumab	48	111
Lymphocyte-depleting	18	rituximab	164	452

[a] Prescription within this number of months before vaccination or before breakthrough infection was used to define use.

© 2022 Vo AD et al. *JAMA Network Open.*

eTable 3. Full Cohort, Data and Analysis, Univariable and Multivariable

	Overall	Non-severe	Severe	Univariable OR (CI)	Multivariable OR (CI)	P (multi)
N	110760	100148	10612			
Sex (%)						
Male	97614 (88.1)	87389 (87.3)	10225 (96.4)	1.0 (referent)	1.0 (referent)	
Female	13146 (11.9)	22759 (22.7)	387 (3.6)	0.26 (0.23 - 0.29)	0.67 (0.60 - 0.75)	<1.0E-08 [a]
Age ranges (%)						
Less than 40	13114 (11.8)	13004 (13.0)	110 (1.0)	0.49 (0.37 - 0.64)	0.57 (0.44 - 0.75)	6.0E-05 [a]
40-45	6241 (5.6)	6153 (6.1)	88 (0.8)	0.83 (0.62 - 1.10)	0.88 (0.66 - 1.18)	0.40
45-50	6307 (5.7)	6200 (6.2)	107 (1.0)	1.0 (referent)	1.0 (referent)	
50-55	9721 (8.8)	9444 (9.4)	277 (2.6)	1.70 (1.36 - 2.13)	1.60 (1.27 - 2.01)	5.0E-5 [a]
55-60	10934 (9.9)	10458 (10.4)	476 (4.5)	2.64 (2.13 - 3.26)	2.24 (1.80 - 2.78)	<1.0E-08 [a]
60-65	12971 (11.7)	12082 (12.1)	889 (8.4)	4.26 (3.48 - 5.22)	3.24 (2.64 - 3.99)	<1.0E-08 [a]
65-70	12353 (11.2)	11013 (11.0)	1340 (12.6)	7.05 (5.78 - 8.61)	4.82 (3.93 - 5.92)	<1.0E-08 [a]
70-75	18327 (16.5)	15703 (15.7)	2624 (24.7)	9.68 (7.96 - 11.8)	6.63 (5.42 - 8.11)	<1.0E-08 [a]
75-80	11562 (10.4)	9546 (9.5)	2016 (19.0)	12.2 (10.1 - 14.9)	8.72 (7.10 - 10.7)	<1.0E-08 [a]
80 or greater	9230 (8.3)	6545 (6.5)	2685 (25.3)	23.8 (19.5 - 28.9)	16.6 (13.5 - 20.4)	<1.0E-08 [a]
Race (%)						
American Indian or Alaska Native	890 (0.8)	802 (0.8)	88 (0.8)	0.92 (0.74 - 1.15)	1.33 (1.04 - 1.71)	0.02 [a]
Asian	1581 (1.4)	1525 (1.5)	56 (0.5)	0.31 (0.24 - 0.40)	0.82 (0.61 - 1.09)	0.17
Black or African American	26953 (24.3)	24790 (24.8)	2163 (20.4)	0.73 (0.69 - 0.77)	0.97 (0.91 - 1.03)	0.28
Native Hawaiian or other Pacific Islander	1142 (1.0)	1066 (1.1)	76 (0.7)	0.60 (0.47 - 0.75)	0.88 (0.68 - 1.13)	0.31
White	71665 (64.7)	64018 (63.9)	7647 (72.1)	1.0 (referent)	1.0 (referent)	
Unknown	8529 (7.7)	7947 (7.9)	582 (5.5)	0.61 (0.56 - 0.67)	0.91 (0.81 - 1.01)	0.06
Ethnicity Hispanic/Latino (%)						
Hispanic or Latino	11259 (10.2)	10505 (10.5)	754 (7.1)	0.64 (0.59 - 0.69)	1.01 (0.93 - 1.11)	0.77
Not Hispanic or Latino	93400 (84.3)	83941 (83.8)	9459 (89.1)	1.0 (referent)	1.0 (referent)	
Unknown	6101 (5.5)	5702 (5.7)	399 (3.8)	0.62 (0.56 - 0.69)	1.03 (0.90 - 1.17)	0.70
US Region (%)						
Continental	18140 (16.4)	16243 (16.2)	1897 (17.9)	1.05 (0.98 - 1.11)	1.16 (1.08 - 1.24)	4.0E-05 [a]
Midwest	21889 (19.8)	19561 (19.5)	2328 (21.9)	1.07 (1.00 - 1.13)	0.99 (0.92 - 1.06)	0.69
North Atlantic	23790 (21.5)	21584 (21.6)	2206 (20.8)	0.92 (0.86 - 0.97)	0.93 (0.87 – 1.00)	0.05
Pacific	20232 (18.3)	18523 (18.5)	1709 (16.1)	0.83 (0.77 - 0.88)	0.96 (0.89 - 1.03)	0.27
Southeast	24404 (22.0)	21954 (21.9)	2450 (23.1)	1.0 (referent)	1.0 (referent)	
Unknown	2305 (2.1)	2283 (2.3)	22 (0.2)	0.09 (0.06 - 0.13)	0.33 (0.21 - 0.50)	3.2E-07 [a]
Period of dominant variant (%)						

© 2022 Vo AD et al. *JAMA Network Open.*

Pre-delta period	2888 (2.6)	2251 (2.2)	637 (6.0)	1.0 (referent)	1.0 (referent)	
Delta period	29493 (26.6)	24762 (24.7)	4731 (44.6)	2.45 (2.35 - 2.55)	0.95 (0.83 - 1.08)	0.42
Omicron period	78379 (70.8)	73135 (73.0)	5244 (49.4)	0.36 (0.35 - 0.38)	0.49 (0.42 - 0.56)	<1.0E-08 [a]
Vaccine type (%)						
Janssen	11066 (10.0)	10028 (10.0)	1038 (9.8)	0.95 (0.88 - 1.02)	1.30 (1.20 - 1.41)	<1.0E-08 [a]
Moderna	45530 (41.1)	41289 (41.2)	4241 (40.0)	0.94 (0.90 - 0.98)	0.79 (0.75 - 0.83)	<1.0E-08 [a]
Pfizer	54164 (48.9)	48831 (48.8)	5333 (50.3)	1.0 (referent)	1.0 (referent)	
History of infection before vaccinated (%)	4373 (3.9)	3959 (4.0)	414 (3.9)	0.99 (0.89 - 1.09)	0.69 (0.61 - 0.78)	<1.0E-08 [a]
Months since full vax at breakthrough (%)						
4 or less	12497 (11.3)	10983 (11.0)	1514 (14.3)	1.0 (referent)	1.0 (referent)	
4-5	7216 (6.5)	6355 (6.3)	861 (8.1)	0.98 (0.90 - 1.07)	1.06 (0.95 - 1.18)	0.33
5-6	8148 (7.4)	7039 (7.0)	1109 (10.5)	1.14 (1.05 - 1.24)	1.01 (0.91 - 1.12)	0.87
6-7	8902 (8.0)	7763 (7.8)	1139 (10.7)	1.06 (0.98 - 1.16)	1.08 (0.97 - 1.20)	0.15
7-8	11739 (10.6)	10665 (10.6)	1074 (10.1)	0.73 (0.67 - 0.79)	1.13 (1.02 - 1.26)	0.02 [a]
8-9	19118 (17.3)	17873 (17.8)	1245 (11.7)	0.51 (0.47 - 0.55)	1.15 (1.03 - 1.28)	0.01 [a]
9-10	19997 (18.1)	18570 (18.5)	1427 (13.4)	0.56 (0.52 - 0.60)	1.16 (1.03 - 1.30)	0.01 [a]
10-11	15731 (14.2)	14252 (14.2)	1479 (13.9)	0.75 (0.70 - 0.81)	1.34 (1.19 - 1.51)	1.4E-06 [a]
11-12	6519 (5.9)	5853 (5.8)	666 (6.3)	0.83 (0.75 - 0.91)	1.47 (1.28 - 1.69)	7.1E-08 [a]
12 or more	893 (0.8)	795 (0.8)	98 (0.9)	0.89 (0.72 - 1.11)	1.57 (1.22 - 2.04)	5.7E-04 [a]
Months since boosted at breakthrough (%)						
Not boosted	81039 (73.2)	72483 (72.4)	8556 (80.6)	1.0 (referent)	1.0 (referent)	
1 or less	5755 (5.2)	5401 (5.4)	354 (3.3)	0.56 (0.50 - 0.62)	0.56 (0.49 - 0.63)	<1.0E-08 [a]
1-2	7732 (7.0)	7341 (7.3)	391 (3.7)	0.45 (0.41 - 0.50)	0.43 (0.38 - 0.48)	<1.0E-08 [a]
2-3	8550 (7.7)	7996 (8.0)	554 (5.2)	0.59 (0.54 - 0.64)	0.46 (0.41 - 0.51)	<1.0E-08 [a]
3-4	4633 (4.2)	4197 (4.2)	436 (4.1)	0.88 (0.80 - 0.97)	0.54 (0.48 - 0.61)	<1.0E-08 [a]
4-5	1749 (1.6)	1543 (1.5)	206 (1.9)	1.13 (0.98 - 1.31)	0.59 (0.50 - 0.70)	<1.0E-08 [a]
5-6	370 (0.3)	320 (0.3)	50 (0.5)	1.32 (0.98 - 1.78)	0.68 (0.48 - 0.95)	0.02 [a]
6 or more	932 (0.8)	867 (0.9)	65 (0.6)	0.64 (0.49 - 0.82)	0.78 (0.59 - 1.03)	0.08
Immune-suppressive medications before breakthrough (%)						
Before breakthrough Chemotherapy	1006 (0.9)	696 (0.7)	310 (2.9)	4.30 (3.76 - 4.92)	2.71 (2.27 - 3.24)	<1.0E-08 [a]
Before breakthrough Cytokine-blocking	1601 (1.4)	1401 (1.4)	200 (1.9)	1.35 (1.17 - 1.57)	1.66 (1.32 - 2.09)	1.6E-05 [a]
Before breakthrough Glucocorticoids	7544 (6.8)	5723 (5.7)	1821 (17.2)	3.42 (3.23 - 3.62)	2.34 (2.18 - 2.50)	<1.0E-08 [a]

© 2022 Vo AD et al. *JAMA Network Open.*

Before breakthrough Leukocyte-inhibitory	1924 (1.7)	1438 (1.4)	486 (4.6)	3.29 (2.97 - 3.66)	2.80 (2.39 - 3.28)	<1.0E-08 [a]
Before breakthrough Lymphocyte-depleting	585 (0.5)	406 (0.4)	179 (1.7)	4.21 (3.53 - 5.03)	2.07 (1.57 - 2.72)	2.1E-07 [a]
Immune-suppressive medications before initial vaccination (%)						
Before vaccine Chemotherapy	430 (0.4)	316 (0.3)	114 (1.1)	3.43 (2.77 - 4.25)	0.86 (0.65 - 1.15)	0.32
Before vaccine Cytokine-blocking	1211 (1.1)	1096 (1.1)	115 (1.1)	0.99 (0.82 - 1.20)	0.60 (0.45 - 0.80)	6.1E-04 [a]
Before vaccine Glucocorticoids	2754 (2.5)	2124 (2.1)	630 (5.9)	2.91 (2.66 - 3.19)	1.38 (1.23 - 1.54)	3.0E-08 [a]
Before vaccine Leukocyte-inhibitory	1368 (1.2)	1099 (1.1)	269 (2.5)	2.34 (2.05 - 2.68)	0.80 (0.65 - 0.98)	0.03 [a]
Before vaccine Lymphocyte-depleting	223 (0.2)	153 (0.2)	70 (0.7)	4.34 (3.27 - 5.76)	1.07 (0.69 - 1.65)	0.77
Persistent positive test (%)	462 (0.4)	359 (0.4)	103 (1.0)	2.72 (2.19 - 3.39)	1.26 (0.98 - 1.62)	0.07
BMI Class (%)						
Underweight	677 (0.6)	496 (0.5)	181 (1.7)	2.14 (1.80 - 2.56)	1.53 (1.24 - 1.87)	5.0E-05 [a]
Normal	14394 (13.0)	12299 (12.3)	2095 (19.7)		1.0 (referent)	
Overweight	32707 (29.5)	29792 (29.7)	2915 (27.5)	0.57 (0.54 - 0.61)	0.71 (0.67 - 0.76)	<1.0E-08 [a]
Obesity I	31156 (28.1)	28557 (28.5)	2599 (24.5)	0.53 (0.50 - 0.57)	0.78 (0.73 - 0.84)	<1.0E-08 [a]
Obesity II	16862 (15.2)	15335 (15.3)	1527 (14.4)	0.58 (0.55 - 0.63)	0.94 (0.87 - 1.02)	0.13
Severe Obesity	10641 (9.6)	9510 (9.5)	1131 (10.7)	0.70 (0.65 - 0.75)	1.23 (1.12 - 1.35)	8.6E-06 [a]
Unknown	4323 (3.9)	4159 (4.2)	164 (1.5)	0.23 (0.20 - 0.27)	0.88 (0.74 - 1.06)	0.17
Comorbidities (%)						
Acquired hypothyroidism	4965 (4.5)	4173 (4.2)	792 (7.5)	1.85 (1.71 - 2.01)	1.03 (0.94 - 1.13)	0.49
Acute myocardial infarction	787 (0.7)	573 (0.6)	214 (2.0)	3.58 (3.05 - 4.19)	1.10 (0.90 - 1.33)	0.36
Alzheimers disease and related disorders or senile dementia	3050 (2.8)	1915 (1.9)	1135 (10.7)	6.14 (5.69 - 6.63)	2.01 (1.83 - 2.20)	<1.0E-08 [a]
Anemia	8134 (7.3)	6268 (6.3)	1866 (17.6)	3.20 (3.02 - 3.38)	1.19 (1.11 - 1.28)	1.6E-06 [a]
Anxiety disorders	26281 (23.7)	24343 (24.3)	1938 (18.3)	0.70 (0.66 - 0.73)	0.90 (0.84 - 0.96)	8.8E-04 [a]
Asthma	3518 (3.2)	3206 (3.2)	312 (2.9)	0.92 (0.81 - 1.03)	0.90 (0.79 - 1.04)	0.15
Atrial fibrillation	5799 (5.2)	4299 (4.3)	1500 (14.1)	3.67 (3.45 - 3.91)	1.18 (1.09 - 1.27)	2.5E-05 [a]
Bipolar disorder	6297 (5.7)	5654 (5.6)	643 (6.1)	1.08 (0.99 - 1.17)	1.24 (1.12 - 1.37)	5.4E-05 [a]
Chronic kidney disease	11832 (10.7)	9071 (9.1)	2761 (26.0)	3.53 (3.36 - 3.71)	1.59 (1.49 - 1.69)	<1.0E-08 [a]
Chronic obstructive pulmonary disease and bronchiectasis	8337 (7.5)	6103 (6.1)	2234 (21.1)	4.11 (3.90 - 4.33)	1.65 (1.54 - 1.76)	<1.0E-08 [a]
Colorectal cancer	608 (0.5)	487 (0.5)	121 (1.1)	2.36 (1.93 - 2.88)	1.04 (0.83 - 1.30)	0.74
Depression	22542 (20.4)	20517 (20.5)	2025 (19.1)	0.92 (0.87 - 0.96)	1.08 (1.01 - 1.16)	0.02 [a]
Diabetes	26083 (23.5)	21919 (21.9)	4164 (39.2)	2.30 (2.21 - 2.40)	1.25 (1.19 - 1.32)	<1.0E-08 [a]
Epilepsy	1081 (1.0)	870 (0.9)	211 (2.0)	2.31 (1.99 - 2.69)	1.49 (1.25 - 1.78)	1.3E-05 [a]

© 2022 Vo AD et al. *JAMA Network Open.*

Heart failure	5444 (4.9)	3681 (3.7)	1763 (16.6)	5.22 (4.91 - 5.55)	1.74 (1.61 - 1.88)	<1.0E-08 [a]
HIV/AIDS	874 (0.8)	791 (0.8)	83 (0.8)	0.99 (0.79 - 1.24)	1.30 (1.01 - 1.68)	0.04
Hyperlipidemia	31511 (28.4)	27624 (27.6)	3887 (36.6)	1.52 (1.46 - 1.58)	0.79 (0.75 - 0.84)	<1.0E-08 [a]
Hypertension	38909 (35.1)	33190 (33.1)	5719 (53.9)	2.36 (2.26 - 2.46)	1.09 (1.03 - 1.15)	0.004 [a]
Ischemic heart disease	11007 (9.9)	8720 (8.7)	2287 (21.6)	2.88 (2.74 - 3.03)	0.98 (0.92 - 1.05)	0.56
Leukemias and lymphomas	1336 (1.2)	993 (1.0)	343 (3.2)	3.34 (2.95 - 3.78)	1.87 (1.61 - 2.17)	<1.0E-08 [a]
Liver disease: cirrhosis and other liver conditions except viral hepatitis	4193 (3.8)	3568 (3.6)	625 (5.9)	1.69 (1.55 - 1.85)	1.36 (1.23 - 1.51)	<1.0E-08 [a]
Lung cancer	824 (0.7)	573 (0.6)	251 (2.4)	4.21 (3.62 - 4.89)	1.61 (1.36 - 1.92)	5.0E-08 [a]
Mobility impairments	1030 (0.9)	728 (0.7)	302 (2.8)	4.00 (3.49 - 4.58)	1.92 (1.63 - 2.26)	<1.0E-08 [a]
Multiple sclerosis and transverse myelitis	454 (0.4)	362 (0.4)	92 (0.9)	2.41 (1.92 - 3.03)	2.86 (2.17 - 3.78)	<1.0E-08 [a]
Peripheral vascular disease	3097 (2.8)	2241 (2.2)	856 (8.1)	3.83 (3.53 - 4.16)	1.26 (1.14 - 1.39)	5.4E-06 [a]
Pressure and chronic ulcers	1347 (1.2)	872 (0.9)	475 (4.5)	5.33 (4.76 - 5.98)	1.58 (1.37 - 1.81)	<1.0E-08 [a]
Prostate cancer	3815 (3.4)	3182 (3.2)	633 (6.0)	1.93 (1.77 - 2.11)	0.90 (0.81 - 0.99)	0.03 [a]
Schizophrenia and other psychotic disorders	2755 (2.5)	2320 (2.3)	435 (4.1)	1.80 (1.62 – 2.00)	1.71 (1.51 - 1.93)	<1.0E-08 [a]
Tobacco use	9322 (8.4)	8114 (8.1)	1208 (11.4)	1.46 (1.37 - 1.55)	1.31 (1.21 - 1.42)	<1.0E-08 [a]
Viral hepatitis	1416 (1.3)	1180 (1.2)	236 (2.2)	1.91 (1.66 - 2.20)	0.98 (0.83 - 1.16)	0.82
Death from breakthrough infection	1555 (1.4)	0 (0.0)	1555 (14.7)			

[a] Meets criteria for statistical significance after adjustment for multiple comparisons, P<0.035 for this analysis. OR = odds ratio. CI = 95% confidence interval.

© 2022 Vo AD et al. *JAMA Network Open.*

eTable 4. Full Cohort Data, 4 Levels of Severity

	Overall	Non-severe Outpatient	Non-severe Hospitalized	Severe, Survived	Severe, Died[a]
N	110760	96070	4078	9057	1555
**Demographic information**					
Sex = male (%)	97614 (88.1)	83524 (86.9)	3865 (94.8)	8691 (96.0)	1534 (98.6)
Age ranges (%)					
Less than 40	13114 (11.8)	12831 (13.4)	173 (4.2)	106 (1.2)	4 (0.3)
40-45	6241 (5.6)	6061 (6.3)	92 (2.3)	85 (0.9)	3 (0.2)
45-50	6307 (5.7)	6103 (6.4)	97 (2.4)	105 (1.2)	2 (0.1)
50-55	9721 (8.8)	9243 (9.6)	201 (4.9)	263 (2.9)	14 (0.9)
55-60	10934 (9.9)	10149 (10.6)	309 (7.6)	444 (4.9)	32 (2.1)
60-65	12971 (11.7)	11579 (12.1)	503 (12.3)	812 (9.0)	77 (5.0)
65-70	12353 (11.2)	10437 (10.9)	576 (14.1)	1190 (13.1)	150 (9.6)
70-75	18327 (16.5)	14901 (15.5)	802 (19.7)	2268 (25.0)	356 (22.9)
75-80	11562 (10.4)	8966 (9.3)	580 (14.2)	1687 (18.6)	329 (21.2)
80 or greater	9230 (8.3)	5800 (6.0)	745 (18.3)	2097 (23.2)	588 (37.8)
Race (%)					
American Indian or Alaska Native	890 (0.8)	771 (0.8)	31 (0.8)	74 (0.8)	14 (0.9)
Asian	1581 (1.4)	1494 (1.6)	31 (0.8)	49 (0.5)	7 (0.5)
Black or African American	26953 (24.3)	23458 (24.4)	1332 (32.7)	1881 (20.8)	282 (18.1)
Native Hawaiian or other Pacific Islander	1142 (1.0)	1029 (1.1)	37 (0.9)	60 (0.7)	16 (1.0)
White	71665 (64.7)	61626 (64.1)	2392 (58.7)	6485 (71.6)	1162 (74.7)
Unknown	8529 (7.7)	7692 (8.0)	255 (6.3)	508 (5.6)	74 (4.8)
Ethnicity Hispanic/Latino (%)					
Hispanic or Latino	11259 (10.2)	10154 (10.6)	351 (8.6)	659 (7.3)	95 (6.1)
Not Hispanic or Latino	93400 (84.3)	80382 (83.7)	3559 (87.3)	8040 (88.8)	1419 (91.3)
Unknown	6101 (5.5)	5534 (5.8)	168 (4.1)	358 (4.0)	41 (2.6)
US Region (%)					
Continental	18140 (16.4)	15626 (16.3)	617 (15.1)	1633 (18.0)	264 (17.0)
Midwest	21889 (19.8)	18789 (19.6)	772 (18.9)	1971 (21.8)	357 (23.0)
North Atlantic	23790 (21.5)	20663 (21.5)	921 (22.6)	1879 (20.7)	327 (21.0)
Pacific	20232 (18.3)	17853 (18.6)	670 (16.4)	1459 (16.1)	250 (16.1)
Southeast	24404 (22.0)	20881 (21.7)	1073 (26.3)	2097 (23.2)	353 (22.7)
Unknown	2305 (2.1)	2258 (2.4)	25 (0.6)	18 (0.2)	4 (0.3)
**Period of dominant variant**					
Pre-delta period (%)	1774 (1.6)	1858 (1.9)	393 (9.6)	546 (6.0)	91 (5.9)

© 2022 Vo AD et al. *JAMA Network Open.*

Delta period (%)	29493 (26.6)	23517 (24.5)	1245 (30.5)	3995 (44.1)	736 (47.3)
Omicron period (%)	78379 (70.8)	70695 (73.6)	2440 (59.8)	4516 (49.9)	728 (46.8)
**Vaccination-related**					
Vaccine type (%)					
Janssen	11066 (10.0)	9652 (10.0)	376 (9.2)	901 (9.9)	137 (8.8)
Moderna	45530 (41.1)	39831 (41.5)	1458 (35.8)	3595 (39.7)	646 (41.5)
Pfizer	54164 (48.9)	46587 (48.5)	2244 (55.0)	4561 (50.4)	772 (49.6)
Months since full vax at breakthrough (%)					
4 or less	12497 (11.3)	10216 (10.6)	767 (18.8)	1311 (14.5)	203 (13.1)
4-5	7216 (6.5)	6078 (6.3)	277 (6.8)	748 (8.3)	113 (7.3)
5-6	8148 (7.4)	6721 (7.0)	318 (7.8)	929 (10.3)	180 (11.6)
6-7	8902 (8.0)	7434 (7.7)	329 (8.1)	951 (10.5)	188 (12.1)
7-8	11739 (10.6)	10301 (10.7)	364 (8.9)	934 (10.3)	140 (9.0)
8-9	19118 (17.3)	17411 (18.1)	462 (11.3)	1069 (11.8)	176 (11.3)
9-10	19997 (18.1)	17948 (18.7)	622 (15.3)	1213 (13.4)	214 (13.8)
10-11	15731 (14.2)	13677 (14.2)	575 (14.1)	1241 (13.7)	238 (15.3)
11-12	6519 (5.9)	5548 (5.8)	305 (7.5)	577 (6.4)	89 (5.7)
12 or more	893 (0.8)	736 (0.8)	59 (1.4)	84 (0.9)	14 (0.9)
Months since boosted at breakthrough (%)					
Not boosted	81039 (73.2)	69486 (72.3)	2997 (73.5)	7295 (80.5)	1261 (81.1)
1 or less	5755 (5.2)	5186 (5.4)	215 (5.3)	310 (3.4)	44 (2.8)
1-2	7732 (7.0)	7098 (7.4)	243 (6.0)	338 (3.7)	53 (3.4)
2-3	8550 (7.7)	7706 (8.0)	290 (7.1)	470 (5.2)	84 (5.4)
3-4	4633 (4.2)	4007 (4.2)	190 (4.7)	370 (4.1)	66 (4.2)
4-5	1749 (1.6)	1454 (1.5)	89 (2.2)	172 (1.9)	34 (2.2)
5-6	370 (0.3)	299 (0.3)	21 (0.5)	45 (0.5)	5 (0.3)
6 or more	932 (0.8)	834 (0.9)	33 (0.8)	57 (0.6)	8 (0.5)
History of infection before vaccination (%)	4373 (3.9)	3678 (3.8)	281 (6.9)	379 (4.2)	35 (2.3)
**Immune-status-related**					
Persistent positive test (%)	462 (0.4)	331 (0.3)	28 (0.7)	103 (1.1)	0 (0.0)
Immune-suppressive medications after vaccination (i.e. before breakthrough) (%)					
Chemotherapy	1006 (0.9)	607 (0.6)	89 (2.2)	235 (2.6)	75 (4.8)
Cytokine-blocking	1601 (1.4)	1334 (1.4)	67 (1.6)	164 (1.8)	36 (2.3)
Glucocorticoids	7544 (6.8)	5375 (5.6)	348 (8.5)	1531 (16.9)	290 (18.6)
Leukocyte-inhibitory	1924 (1.7)	1334 (1.4)	104 (2.6)	366 (4.0)	120 (7.7)
Lymphocyte-depleting	585 0.5)	374 (0.4)	32 (0.8)	132 (1.5)	47 (3.0)

© 2022 Vo AD et al. *JAMA Network Open.*

Immune-suppressive medications before initial vaccination (%)					
Chemotherapy	430 (0.4)	291 (0.3)	25 (0.6)	79 (0.9)	35 (2.3)
Cytokine-blocking	1211 (1.1)	1047 (1.1)	49 (1.2)	97 (1.1)	18 (1.2)
Glucocorticoids	2754 (2.5)	1981 (2.1)	143 (3.5)	515 (5.7)	115 (7.4)
Leukocyte-inhibitory	1368 (1.2)	1042 (1.1)	57 (1.4)	206 (2.3)	63 (4.1)
Lymphocyte-depleting	223 (0.2)	141 (0.1)	12 (0.3)	51 (0.6)	19 (1.2)
BMI Class (%)					
Underweight	677 (0.6)	435 (0.5)	61 (1.5)	142 (1.6)	39 (2.5)
Normal	14394 (13.0)	11420 (11.9)	879 (21.6)	1699 (18.8)	396 (25.5)
Overweight	32707 (29.5)	28448 (29.6)	1344 (33.0)	2477 (27.3)	438 (28.2)
Obesity I	31156 (28.1)	27612 (28.7)	945 (23.2)	2237 (24.7)	362 (23.3)
Obesity II	16862 (15.2)	14864 (15.5)	471 (11.5)	1351 (14.9)	176 (11.3)
Severe Obesity	10641 (9.6)	9221 (9.6)	289 (7.1)	1008 (11.1)	123 (7.9)
Unknown	4323 (3.9)	4070 (4.2)	89 (2.2)	143 (1.6)	21 (1.4)
**Comorbidities (%)**					
Acquired hypothyroid	4965 (4.5)	3945 (4.1)	228 (5.6)	655 (7.2)	137 (8.8)
Acute myocardial infarction	787 (0.7)	493 (0.5)	80 (2.0)	191 (2.1)	23 (1.5)
Alzheimers disease and related disorders or senile dementia	3050 (2.8)	1526 (1.6)	389 (9.5)	915 (10.1)	220 (14.1)
Anemia	8134 (7.3)	5507 (5.7)	761 (18.7)	1546 (17.1)	320 (20.6)
Anxiety disorders	26281 (23.7)	23412 (24.4)	931 (22.8)	1717 (19.0)	221 (14.2)
Asthma	3518 (3.2)	3083 (3.2)	123 (3.0)	284 (3.1)	28 (1.8)
Atrial fibrillation	5799 (5.2)	3843 (4.0)	456 (11.2)	1258 (13.9)	242 (15.6)
Bipolar disorder	6297 (5.7)	5271 (5.5)	383 (9.4)	577 (6.4)	66 (4.2)
Chronic kidney disease	11832 (10.7)	8060 (8.4)	1011 (24.8)	2293 (25.3)	468 (30.1)
Chronic obstructive pulmonary disease and bronchiectasis	8337 (7.5)	5669 (5.9)	434 (10.6)	1947 (21.5)	287 (18.5)
Colorectal cancer	608 (0.5)	442 (0.5)	45 (1.1)	96 (1.1)	25 (1.6)
Depression	22542 (20.4)	19538 (20.3)	979 (24.0)	1786 (19.7)	239 (15.4)
Diabetes	26083 (23.5)	20471 (21.3)	1448 (35.5)	3534 (39.0)	630 (40.5)
Epilepsy	1081 (1.0)	768 (0.8)	102 (2.5)	184 (2.0)	27 (1.7)
Heart failure	5444 (4.9)	3156 (3.3)	525 (12.9)	1470 (16.2)	293 (18.8)
Human immunodeficiency virus and/or acquired immunodeficiency syndrome HIV/AIDS	874 (0.8)	752 (0.8)	39 (1.0)	72 (0.8)	11 (0.7)
Hyperlipidemia	31511 (28.4)	26140 (27.2)	1484 (36.4)	3360 (37.1)	527 (33.9)
Hypertension	38909 (35.1)	31113 (32.4)	2077 (50.9)	4860 (53.7)	859 (55.2)
Ischemic heart disease	11007 (9.9)	7949 (8.3)	771 (18.9)	1921 (21.2)	366 (23.5)

© 2022 Vo AD et al. *JAMA Network Open.*

Leukemias and lymphomas	1336 (1.2)	922 (1.0)	71 (1.7)	247 (2.7)	96 (6.2)
Liver disease cirrhosis and other liver conditions except viral hepatitis	4193 (3.8)	3267 (3.4)	301 (7.4)	521 (5.8)	104 (6.7)
Lung cancer	824 (0.7)	525 (0.5)	48 (1.2)	200 (2.2)	51 (3.3)
Mobility impairments	1030 (0.9)	580 (0.6)	148 (3.6)	271 (3.0)	31 (2.0)
Multiple sclerosis and transverse myelitis	454 (0.4)	332 (0.3)	30 (0.7)	85 (0.9)	7 (0.5)
Peripheral vascular disease	3097 (2.8)	1946 (2.0)	295 (7.2)	699 (7.7)	157 (10.1)
Pressure and chronic ulcers	1347 (1.2)	656 (0.7)	216 (5.3)	390 (4.3)	85 (5.5)
Prostate cancer	3815 (3.4)	2943 (3.1)	239 (5.9)	529 (5.8)	104 (6.7)
Schizophrenia and other psychotic disorders	2755 (2.5)	2095 (2.2)	225 (5.5)	382 (4.2)	53 (3.4)
Tobacco use	9322 (8.4)	7560 (7.9)	554 (13.6)	1092 (12.1)	116 (7.5)
Viral hepatitis	1416 (1.3)	1052 (1.1)	128 (3.1)	206 (2.3)	30 (1.9)

[a] We explored whether use of ICD-10 codes could be used to improve specificity among hospitalized patients, particularly since use was lower among patients who died or required mechanical ventilation. However, review of 25 cases of patients who died indicated that COVID-19 was the cause of death in the great majority even if the ICD-10 codes were not used: 11/13 with use of the code vs. 10/12 without, and with SARS-CoV-2 likely contributing to complex illnesses in several of the other cases. Therefore, ICD-10 codes for COVID-19 were not used in case definitions.

© 2022 Vo AD et al. *JAMA Network Open.*

eTable 5. Subcohorts, Data for Patients Younger and Older Than 50 Years, Analysis for Patients Younger Than 50 Years

	Greater than age 50	Less than age 50	Non-severe, less than 50	Severe, less than 50	Multivariable OR (CI)	P
N	85098	25662	25357	305		
Sex (%)						
Male	78268 (92.0)	19346 (75.4)	19082 (75.3)	264 (86.6)		
Female	6830 (8.0)	6316 (24.6)	6275 (24.7)	41 (13.4)	0.47 (0.33 - 0.67)	3.1E-05 [a]
Age ranges (%)						
Less than 40		13114 (51.1)	13004 (51.3)	110 (36.1)		
40-45		6241 (24.3)	6153 (24.3)	88 (28.9)		
45-50		6307 (24.6)	6200 (24.5)	107 (35.1)		
50-55	9721 (11.4)					
55-60	10934 (12.8)					
60-65	12971 (15.2)					
65-70	12353 (14.5)					
70-75	18327 (21.5)					
75-80	11562 (13.6)					
80 or greater	9230 (10.8)					
Age per 5-year increase					1.22 (1.10 - 1.36)	1.2E-04 [a]
Race (%)						
American Indian or Alaska Native	626 (0.7)	264 (1.0)	256 (1.0)	8 (2.6)	2.96 (1.39 - 6.28)	0.005 [a]
Asian	759 (0.9)	822 (3.2)	816 (3.2)	6 (2.0)	0.85 (0.37 - 1.97)	0.70
Black or African American	21252 (25.0)	5701 (22.2)	5638 (22.2)	63 (20.7)	0.84 (0.61 - 1.15)	0.27
Native Hawaiian or other Pacific Islander	798 (0.9)	344 (1.3)	342 (1.3)	2 (0.7)	0.57 (0.14 - 2.34)	0.44
White	5625 (6.6)	2904 (11.3)	2874 (11.3)	30 (9.8)	1.0 (referent)	
Unknown	56038 (65.9)	15627 (60.9)	15431 (60.9)	196 (64.3)	1.00 (0.64 - 1.55)	0.99
Ethnicity Hispanic/Latino (%)						
Hispanic or Latino	6850 (8.0)	4409 (17.2)	4365 (17.2)	44 (14.4)	0.87 (0.61 - 1.24)	0.45
Not Hispanic or Latino	74494 (87.5)	18906 (73.7)	18669 (73.6)	237 (77.7)	1.0 (referent)	
Unknown	3754 (4.4)	2347 (9.1)	2323 (9.2)	24 (7.9)	1.36 (0.82 - 2.25)	0.23
US Region (%)						
Continental	13575 (16.0)	4565 (17.8)	4492 (17.7)	73 (23.9)	1.10 (0.78 - 1.55)	0.59
Midwest	17725 (20.8)	4164 (16.2)	4113 (16.2)	51 (16.7)	0.83 (0.56 - 1.22)	0.34
North Atlantic	18426 (21.7)	5364 (20.9)	5309 (20.9)	55 (18.0)	0.75 (0.52 - 1.09)	0.13
Pacific	14739 (17.3)	5493 (21.4)	5441 (21.5)	52 (17.0)	0.79 (0.53 - 1.15)	0.22

© 2022 Vo AD et al. *JAMA Network Open.*

Southeast	19480 (22.9)	4924 (19.2)	4855 (19.1)	69 (22.6)	1.0 (referent)	
Unknown	1153 (1.4)	1152 (4.5)	1147 (4.5)	5 (1.6)	0.38 (0.15 - 0.97)	0.04
Period of dominant variant (%)						
Pre-delta period					1.0 (referent)	
Delta period	24712 (29.0)	4781 (18.6)	4666 (18.4)	115 (37.7)	0.93 (0.38 - 2.26)	0.88
Omicron period	57746 (67.9)	20633 (80.4)	20449 (80.6)	184 (60.3)	0.41 (0.17 - 1.01)	0.05
Vaccine type (%)						
Janssen	7503 (8.8)	3563 (13.9)	3496 (13.8)	67 (22.0)	1.45 (1.06 - 1.98)	0.02
Moderna	36379 (42.7)	9151 (35.7)	9059 (35.7)	92 (30.2)	0.89 (0.68 - 1.17)	0.42
Pfizer	41216 (48.4)	12948 (50.5)	12802 (50.5)	146 (47.9)	1.0 (referent)	
History of infection before vaccinated (%)	3172 (3.7)	1201 (4.7)	1187 (4.7)	14 (4.6)	0.82 (0.45 - 1.47)	0.50
Months since full vax at breakthrough (%)						
4 or less	8486 (10.0)	4011 (15.6)	3943 (15.5)	68 (22.3)	1.0 (referent)	
4-5	5379 (6.3)	1837 (7.2)	1804 (7.1)	33 (10.8)	1.05 (0.67 - 1.63)	0.84
5-6	6521 (7.7)	1627 (6.3)	1601 (6.3)	26 (8.5)	1.04 (0.64 - 1.68)	0.88
6-7	6700 (7.9)	2202 (8.6)	2170 (8.6)	32 (10.5)	1.11 (0.70 - 1.74)	0.66
7-8	7930 (9.3)	3809 (14.8)	3770 (14.9)	39 (12.8)	0.99 (0.64 - 1.53)	0.95
8-9	13320 (15.7)	5798 (22.6)	5757 (22.7)	41 (13.4)	0.80 (0.51 - 1.25)	0.32
9-10	16676 (19.6)	3321 (12.9)	3289 (13.0)	32 (10.5)	1.10 (0.67 - 1.78)	0.71
10-11	13907 (16.3)	1824 (7.1)	1806 (7.1)	18 (5.9)	1.22 (0.67 - 2.21)	0.52
11-12	5455 (6.4)	1064 (4.1)	1048 (4.1)	16 (5.2)	2.29 (1.21 - 4.31)	0.01
12 or more	724 (0.9)	169 (0.7)	169 (0.7)	0 (0.0)	ND	
Months since boosted at breakthrough (%)						
Not boosted	59003 (69.3)	22036 (85.9)	21758 (85.8)	278 (91.1)	1.0 (referent)	
1 or less	4655 (5.5)	1100 (4.3)	1093 (4.3)	7 (2.3)	0.67 (0.31 - 1.46)	0.31
1-2	6656 (7.8)	1076 (4.2)	1071 (4.2)	5 (1.6)	0.48 (0.19 - 1.19)	0.11
2-3	7825 (9.2)	725 (2.8)	719 (2.8)	6 (2.0)	0.61 (0.26 - 1.44)	0.26
3-4	4343 (5.1)	290 (1.1)	287 (1.1)	3 (1.0)	0.57 (0.17 - 1.91)	0.36
4-5	1624 (1.9)	125 (0.5)	123 (0.5)	2 (0.7)	0.51 (0.11 - 2.43)	0.40
5-6	330 (0.4)	40 (0.2)	39 (0.2)	1 (0.3)	1.03 (0.11 - 9.45)	0.98
6 or more	662 (0.8)	270 (1.1)	267 (1.1)	3 (1.0)	1.19 (0.37 - 3.88)	0.77
Immune-suppressive medications before breakthrough (%)						
Chemotherapy (%)	942 (1.1)	64 (0.2)	62 (0.2)	2 (0.7)	1.36 (0.22 - 8.38)	0.74
Cytokine-blocking (%)	1251 (1.5)	350 (1.4)	346 (1.4)	4 (1.3)	1.72 (0.52 - 5.71)	0.37
Glucocorticoids (%)	6467 (7.6)	1077 (4.2)	1039 (4.1)	38 (12.5)	2.35 (1.59 - 3.47)	1.6E-05 [a]
Leukocyte-inhibitory (%)	1702 (2.0)	222 (0.9)	215 (0.8)	7 (2.3)	1.02 (0.35 - 2.96)	0.97
Lymphocyte-depleting (%)	497 (0.6)	88 (0.3)	80 (0.3)	8 (2.6)	1.62 (0.51 - 5.18)	0.42
Immune-suppressive medications before initial vaccination (%)						

© 2022 Vo AD et al. *JAMA Network Open.*

Chemotherapy (%)	391 (0.5)	39 (0.2)	38 (0.1)	1 (0.3)	0.71 (0.05 - 9.34)	0.79
Cytokine-blocking (%)	922 (1.1)	289 (1.1)	288 (1.1)	1 (0.3)	0.13 (0.01 - 1.30)	0.08
Glucocorticoids (%)	2377 (2.8)	377 (1.5)	360 (1.4)	17 (5.6)	1.84 (1.01 - 3.33)	0.05
Leukocyte-inhibitory (%)	1209 (1.4)	159 (0.6)	154 (0.6)	5 (1.6)	1.48 (0.45 - 4.93)	0.52
Lymphocyte-depleting (%)	193 (0.2)	30 (0.1)	28 (0.1)	2 (0.7)	0.59 (0.09 - 3.80)	0.58
Persistent positive test (%)	404 (0.5)	58 (0.2)	56 (0.2)	2 (0.7)	1.09 (0.25 - 4.88)	0.91
BMI Class (%)						
Underweight	591 (0.7)	86 (0.3)	83 (0.3)	3 (1.0)	2.83 (0.70 - 11.4)	0.14
Normal	11472 (13.5)	2922 (11.4)	2890 (11.4)	32 (10.5)	1.0 (referent)	
Overweight	25795 (30.3)	6912 (26.9)	6851 (27.0)	61 (20.0)	0.76 (0.49 - 1.19)	0.23
Obesity I	24261 (28.5)	6895 (26.9)	6798 (26.8)	97 (31.8)	1.16 (0.76 - 1.77)	0.50
Obesity II	12843 (15.1)	4019 (15.7)	3978 (15.7)	41 (13.4)	0.73 (0.44 - 1.19)	0.20
Severe Obesity	7870 (9.2)	2771 (10.8)	2715 (10.7)	56 (18.4)	1.24 (0.77 – 2.00)	0.37
Unknown	2266 (2.7)	2057 (8.0)	2042 (8.1)	15 (4.9)	0.89 (0.45 - 1.74)	0.73
Comorbidities (%)						
Acquired hypothyroidism	4393 (5.2)	572 (2.2)	561 (2.2)	11 (3.6)	1.03 (0.51 - 2.05)	0.94
Acute myocardial infarction	749 (0.9)	38 (0.1)	31 (0.1)	7 (2.3)	2.08 (0.54 – 8.00)	0.29
Alzheimers disease and related disorders or senile dementia	3005 (3.5)	45 (0.2)	39 (0.2)	6 (2.0)	5.33 (1.83 - 15.6)	0.002 [a]
Anemia	7295 (8.6)	839 (3.3)	810 (3.2)	29 (9.5)	1.64 (1.02 - 2.63)	0.04
Anxiety disorders	16675 (19.6)	9606 (37.4)	9475 (37.4)	131 (43.0)	1.06 (0.81 - 1.40)	0.66
Asthma	2524 (3.0)	994 (3.9)	973 (3.8)	21 (6.9)	1.48 (0.92 - 2.39)	0.10
Atrial fibrillation	5701 (6.7)	98 (0.4)	96 (0.4)	2 (0.7)	0.58 (0.13 - 2.66)	0.49
Bipolar disorder	4288 (5.0)	2009 (7.8)	1969 (7.8)	40 (13.1)	1.34 (0.92 - 1.96)	0.13
Chronic kidney disease	11069 (13.0)	763 (3.0)	715 (2.8)	48 (15.7)	2.38 (1.55 - 3.66)	7.8E-05 [a]
Chronic obstructive pulmonary disease and bronchiectasis	8150 (9.6)	187 (0.7)	177 (0.7)	10 (3.3)	1.81 (0.88 - 3.74)	0.11
Colorectal cancer	589 (0.7)	19 (0.1)	19 (0.1)	0 (0.0)	ND	
Depression	15229 (17.9)	7313 (28.5)	7206 (28.4)	107 (35.1)	1.05 (0.78 - 1.40)	0.76
Diabetes	24430 (28.7)	1653 (6.4)	1595 (6.3)	58 (19.0)	1.57 (1.07 - 2.29)	0.02
Epilepsy	891 (1.0)	190 (0.7)	183 (0.7)	7 (2.3)	1.29 (0.53 - 3.12)	0.57
Heart failure	5322 (6.3)	122 (0.5)	110 (0.4)	12 (3.9)	2.02 (0.92 - 4.47)	0.08
Human immunodeficiency virus and/or acquired immunodeficiency syndrome HIV/AIDS	688 (0.8)	186 (0.7)	184 (0.7)	2 (0.7)	0.64 (0.15 - 2.67)	0.54
Hyperlipidemia	27594 (32.4)	3917 (15.3)	3852 (15.2)	65 (21.3)	0.66 (0.48 - 0.92)	0.01
Hypertension	35573 (41.8)	3336 (13.0)	3251 (12.8)	85 (27.9)	1.23 (0.89 - 1.69)	0.20
Ischemic heart disease	10764 (12.6)	243 (0.9)	228 (0.9)	15 (4.9)	1.13 (0.51 - 2.53)	0.76
Leukemias and lymphomas	1268 (1.5)	68 (0.3)	61 (0.2)	7 (2.3)	6.34 (2.38 - 16.9)	2.2E-04 [a]

© 2022 Vo AD et al. *JAMA Network Open.*

Liver disease cirrhosis and other liver conditions except viral hepatitis	3386 (4.0)	807 (3.1)	776 (3.1)	31 (10.2)	1.85 (1.21 - 2.85)	0.005 [a]
Lung cancer	820 (1.0)	4 (0.0)	4 (0.0)	0 (0.0)	ND	
Mobility impairments	939 (1.1)	91 (0.4)	80 (0.3)	11 (3.6)	3.98 (1.83 - 8.65)	5.0E-04 [a]
Multiple sclerosis and transverse myelitis	326 (0.4)	128 (0.5)	118 (0.5)	10 (3.3)	4.71 (1.82 - 12.2)	0.001 [a]
Peripheral vascular disease	3050 (3.6)	47 (0.2)	41 (0.2)	6 (2.0)	2.26 (0.62 - 8.24)	0.22
Pressure and chronic ulcers	1306 (1.5)	41 (0.2)	35 (0.1)	6 (2.0)	1.05 (0.30 - 3.69)	0.94
Prostate cancer	3803 (4.5)	12 (0.0)	12 (0.0)	0 (0.0)	ND	
Schizophrenia and other psychotic disorders	2148 (2.5)	607 (2.4)	590 (2.3)	17 (5.6)	1.61 (0.92 - 2.81)	0.09
Tobacco use	7134 (8.4)	2188 (8.5)	2140 (8.4)	48 (15.7)	1.21 (0.84 - 1.73)	0.30
Viral hepatitis	1310 (1.5)	106 (0.4)	101 (0.4)	5 (1.6)	1.98 (0.70 - 5.60)	0.20
Death from breakthrough infection	1546 (1.8)	9 (0.0)	0 (0.0)	9 (0.3)		

[a] Meets criteria for statistical significance after adjustment for multiple comparisons, P<0.006 for this analysis. OR = odds ratio. CI = 95% confidence interval.

© 2022 Vo AD et al. *JAMA Network Open.*

eAppendix. Additional Results

Use of ICD-10 codes in the definition of hospitalization for Covid-19?

We explored whether use of ICD-10 codes (U07.1 or J12.82 during hospitalization) could be used to improve specificity among hospitalized patients, particularly since use was lower in the group of patients who died or required mechanical ventilation (79.3% versus 91.4% in other hospitalized patients). However, review of 25 randomly selected cases of patients who died indicated that Covid-19 was the cause of death in the great majority who died within 28 days even if the ICD-10 codes were not used: 11/13 with use of the code vs. 10/12 without, and with SARS-CoV-2 likely contributing to complex illnesses in several of the other cases. Therefore, ICD-10 codes for COVID-19 were not used to refine case definitions.

Body Mass Index (BMI)

After adjustment for comorbidities, the only BMI classes strongly associated with risk were underweight (BMI < 18, OR 1.53, CI 1.24 - 1.87) and severe obesity (BMI > 40, OR 1.23, CI 1.12 - 1.35) relative to normal weight. In addition to low BMI, two other surrogate markers of poor health were significantly associated with risk: mobility impairments (OR 1.92, CI 1.63 – 2.26) and pressure ulcers (OR 1.58, CI 1.37 – 1.81).

Monoclonal Antibody Therapy:

At least 8,522 patients received monoclonal antibody (MAB) therapy prior to hospitalization to prevent disease progression (list of drug names is in **eTable 2**). Among 14,021 immunocompromised patients, 2,073 (14.8%) received MAB therapy, compared to 6,467 (6.7%) of non-immunocompromised patients. Immunocompromised patients treated with MAB had a 12.3% rate of severe disease, compared with 21.1% among all immunocompromised patients. Among non-immunocompromised patients not adjusted for potential confounding factors that may impact probability of progression to severe disease, patients treated with MAB had an 8.5% rate of severe disease, compared to 7.9% among all non-IC patients. Use of MAB was not included in statistical modeling, for two reasons. First, it is given only to outpatients after a positive test, and only to those considered to be at high risk of severe disease. Therefore, outside of a prospective trial, there are reasons to expect it to be associated with both non-severe and severe disease. Second, it is anticipated that data on use of MAB are likely to be missing, due to receipt outside the VA.

Patients <50 Years of Age:

The characteristics of patients < 50 and > 50 are shown in **eTable 5**, as are the results of multivariable logistic regression for the group < 50. Overall risk of severe disease in patients < 50 was low (305 / 25,662 = 1.2%), and death was rare (9 / 25,662 = 0.04%). The low number of severe cases in this group limits interpretability; however, a small set of risk factors indicating severe chronic health problems met criteria for statistical significance after correction for multiple comparisons: CKD, hematologic malignancy, multiple sclerosis, chronic liver disease, Alzheimer's/dementia, mobility impairments, and receipt of glucocorticoids shortly before breakthrough infection. The self-reported racial category of American Indian or Alaska Native also met criteria for statistical significance. Although this finding cannot be ignored, the fact that

© 2022 Vo AD et al. *JAMA Network Open.*

it is based on results for only 264 patients is reason to anticipate that it may not be valid. Examination of results for other subgroups in the **eTables** makes it clear that excess risk in this racial/ethnic group was driven by a small (but higher than other ethnicities) number of severe cases in non-immune-suppressed, relatively young patients.

Urban and rural location

The most recent residence listed in VA records was used. For patients with multiple residences, the one closest to the vaccination site was used. Locations were defined as urban per US Census criteria, and all others were considered rural. VA also defines census regions as highly rural if they contain fewer than 7 residents per square mile. However, since only 0.4% (425 / 110,760) of patients in this study resided in highly rural areas, they were combined with the rural group for analysis. 3.8% of patients had unknown classification of residence and were excluded from this subset analysis. Severity data were missing for 110 / 21,007 (0.52%) of rural and 267 / 71,432 (0.37%) of urban patients, indicating that there was not skewed usage of non-VA hospitals, at least not those contracted with the VA for reimbursement. Among all patients with a hospitalization documented, 1.3% of the 12,856 patients for whom severity could be determined were hospitalized only at a non-VA hospital, compared to 21.5% of the 391 patients for whom severity could not be determined and who were therefore excluded from the study.

eTable 6. Subcohort Immunocompromised, Data and Analysis

	Overall	Non-severe	Severe	Multivariable OR (CI)	P
N	14021	11062	2959		
Sex (%)					
Male	12505 (89.2)	9653 (87.3)	2852 (96.4)	1.0 (referent)	
Female	1516 (10.8)	1409 (12.7)	107 (3.6)	0.59 (0.47 - 0.74)	4.0E-06 [a]
Age ranges (%)					
Less than 40	848 (6.0)	829 (7.5)	19 (0.6)	0.69 (0.37 - 1.29)	0.25
40-45	524 (3.7)	506 (4.6)	18 (0.6)	0.98 (0.52 - 1.86)	0.95
45-50	636 (4.5)	613 (5.5)	23 (0.8)	1.0 (referent)	
50-55	1004 (7.2)	925 (8.4)	79 (2.7)	2.06 (1.27 - 3.36)	0.004 [a]
55-60	1228 (8.8)	1093 (9.9)	135 (4.6)	2.52 (1.58 - 4.02)	9.9E-05 [a]
60-65	1733 (12.4)	1467 (13.3)	266 (9.0)	3.51 (2.24 - 5.51)	4.3E-08 [a]
65-70	1874 (13.4)	1452 (13.1)	422 (14.3)	5.01 (3.21 - 7.83)	<1.0E-08 [a]
70-75	2955 (21.1)	2122 (19.2)	833 (28.2)	6.79 (4.37 - 10.6)	<1.0E-08 [a]
75-80	1841 (13.1)	1274 (11.5)	567 (19.2)	7.71 (4.93 - 12.1)	<1.0E-08 [a]
80 or greater	1378 (9.8)	781 (7.1)	597 (20.2)	12.7 (8.04 - 19.9)	<1.0E-08 [a]
Race (%)					
American Indian or Alaska Native	95 (0.7)	81 (0.7)	14 (0.5)	0.98 (0.52 - 1.82)	0.94
Asian	117 (0.8)	102 (0.9)	15 (0.5)	1.08 (0.59 - 1.99)	0.80
Black or African American	3339 (23.8)	2691 (24.3)	648 (21.9)	1.07 (0.95 - 1.21)	0.25
Native Hawaiian or other Pacific Islander	130 (0.9)	110 (1.0)	20 (0.7)	0.89 (0.53 - 1.50)	0.66
White	9554 (68.1)	7438 (67.2)	2116 (71.5)	1.0 (referent)	
Unknown	786 (5.6)	640 (5.8)	146 (4.9)	0.99 (0.79 - 1.24)	0.93
Ethnicity Hispanic/Latino (%)					
Hispanic or Latino	1094 (7.8)	912 (8.2)	182 (6.2)	1.06 (0.87 - 1.29)	0.55
Not Hispanic or Latino	12426 (88.6)	9731 (88.0)	2695 (91.1)	1.0 (referent)	
Unknown	501 (3.6)	419 (3.8)	82 (2.8)	0.94 (0.70 - 1.25)	0.66
US Region (%)					
Continental	2314 (16.5)	1835 (16.6)	479 (16.2)	1.03 (0.89 - 1.19)	0.68
Midwest	2973 (21.2)	2297 (20.8)	676 (22.8)	0.95 (0.83 - 1.09)	0.45
North Atlantic	3047 (21.7)	2419 (21.9)	628 (21.2)	0.88 (0.76 - 1.00)	0.06
Pacific	2096 (14.9)	1665 (15.1)	431 (14.6)	0.95 (0.82 - 1.11)	0.55
Southeast	3449 (24.6)	2708 (24.5)	741 (25.0)	1.0 (referent)	
Unknown	142 (1.0)	138 (1.2)	4 (0.1)	0.30 (0.10 - 0.84)	0.02 [a]
Period of dominant variant (%)					
Pre-delta period	501 (3.6)	299 (2.7)	202 (6.8)	1.0 (referent)	

© 2022 Vo AD et al. *JAMA Network Open.*

Delta period	4422 (31.5)	3146 (28.4)	1276 (43.1)	0.74 (0.57 - 0.96)	0.02 [a]
Omicron period	9098 (64.9)	7617 (68.9)	1481 (50.1)	0.44 (0.33 - 0.58)	1.2E-08 [a]
Vaccine type (%)					
Janssen	1172 (8.4)	940 (8.5)	232 (7.8)	1.11 (0.93 - 1.33)	0.24
Moderna	6098 (43.5)	4860 (43.9)	1238 (41.8)	0.78 (0.71 - 0.86)	5.7E-07 [a]
Pfizer	6751 (48.1)	5262 (47.6)	1489 (50.3)	1.0 (referent)	
History of infection before vaccinated (%)	636 (4.5)	515 (4.7)	121 (4.1)	0.61 (0.48 - 0.78)	8.6E-05 [a]
Months since full vax at breakthrough (%)					
4 or less	1672 (11.9)	1225 (11.1)	447 (15.1)	1.0 (referent)	
4-5	975 (7.0)	743 (6.7)	232 (7.8)	1.09 (0.86 - 1.37)	0.49
5-6	1159 (8.3)	831 (7.5)	328 (11.1)	1.14 (0.91 - 1.41)	0.25
6-7	1140 (8.1)	845 (7.6)	295 (10.0)	1.04 (0.83 - 1.30)	0.74
7-8	1336 (9.5)	1083 (9.8)	253 (8.6)	0.93 (0.74 - 1.17)	0.52
8-9	2069 (14.8)	1759 (15.9)	310 (10.5)	0.99 (0.79 - 1.24)	0.90
9-10	2488 (17.7)	2070 (18.7)	418 (14.1)	1.13 (0.90 - 1.42)	0.30
10-11	2160 (15.4)	1709 (15.4)	451 (15.2)	1.33 (1.04 - 1.70)	0.02 [a]
11-12	915 (6.5)	710 (6.4)	205 (6.9)	1.39 (1.05 - 1.85)	0.02 [a]
12 or more	107 (0.8)	87 (0.8)	20 (0.7)	0.94 (0.53 - 1.68)	0.84
Months since boosted at breakthrough (%)					
Not boosted	9408 (67.1)	7234 (65.4)	2174 (73.5)	1.0 (referent)	
1 or less	663 (4.7)	541 (4.9)	122 (4.1)	0.78 (0.62 - 0.99)	0.04
1-2	878 (6.3)	734 (6.6)	144 (4.9)	0.63 (0.51 - 0.78)	1.8E-05 [a]
2-3	1107 (7.9)	903 (8.2)	204 (6.9)	0.69 (0.57 - 0.83)	1.0E-04 [a]
3-4	1040 (7.4)	883 (8.0)	157 (5.3)	0.51 (0.41 - 0.63)	<1.0E-08 [a]
4-5	670 (4.8)	563 (5.1)	107 (3.6)	0.54 (0.42 - 0.70)	2.1E-06 [a]
5-6	141 (1.0)	109 (1.0)	32 (1.1)	0.78 (0.49 - 1.23)	0.29
6 or more	114 (0.8)	95 (0.9)	19 (0.6)	0.81 (0.47 - 1.41)	0.46
Immune-suppressive medications before breakthrough (%)					
Before breakthrough Chemotherapy	1006 (7.2)	696 (6.3)	310 (10.5)	2.21 (1.83 - 2.67)	<1.0E-08 [a]
Before breakthrough Cytokine-blocking	1601 (11.4)	1401 (12.7)	200 (6.8)	1.41 (1.12 - 1.78)	0.004 [a]
Before breakthrough Glucocorticoids	7544 (53.8)	5723 (51.7)	1821 (61.5)	1.91 (1.70 - 2.14)	<1.0E-08 [a]
Before breakthrough Leukocyte-inhibitory	1924 (13.7)	1438 (13.0)	486 (16.4)	2.42 (2.05 - 2.85)	<1.0E-08 [a]
Before breakthrough Lymphocyte-depleting	585 (4.2)	406 (3.7)	179 (6.0)	1.94 (1.47 - 2.54)	<1.0E-08 [a]
Immune-suppressive medications before initial vaccination (%)					
Before vaccine Chemotherapy	430 (3.1)	316 (2.9)	114 (3.9)	0.87 (0.66 - 1.15)	0.32
Before vaccine Cytokine-blocking	1211 (8.6)	1096 (9.9)	115 (3.9)	0.59 (0.45 - 0.79)	2.9E-04 [a]
Before vaccine Glucocorticoids	2754 (19.6)	2124 (19.2)	630 (21.3)	1.22 (1.08 - 1.38)	0.002 [a]
Before vaccine Leukocyte-inhibitory	1368 (9.8)	1099 (9.9)	269 (9.1)	0.78 (0.64 - 0.95)	0.01 [a]

© 2022 Vo AD et al. *JAMA Network Open.*

Before vaccine Lymphocyte-depleting	223 (1.6)	153 (1.4)	70 (2.4)	1.12 (0.73 - 1.70)	0.61
Persistent positive test (%)	111 (0.8)	74 (0.7)	37 (1.3)	1.11 (0.71 - 1.72)	0.65
BMI Class (%)					
Underweight	119 (0.8)	60 (0.5)	59 (2.0)	1.67 (1.11 - 2.53)	0.01 [a]
Normal	1994 (14.2)	1372 (12.4)	622 (21.0)	1.0 (referent)	
Overweight	4085 (29.1)	3271 (29.6)	814 (27.5)	0.65 (0.57 - 0.75)	<1.0E-08 [a]
Obesity I	3883 (27.7)	3155 (28.5)	728 (24.6)	0.69 (0.60 - 0.80)	6.7E-07 [a]
Obesity II	2204 (15.7)	1798 (16.3)	406 (13.7)	0.76 (0.64 - 0.90)	0.001 [a]
Severe Obesity	1568 (11.2)	1252 (11.3)	316 (10.7)	0.93 (0.77 - 1.12)	0.44
Unknown	168 (1.2)	154 (1.4)	14 (0.5)	0.55 (0.30 - 1.01)	0.05
Comorbidities (%)					
Acquired hypothyroidism	834 (5.9)	600 (5.4)	234 (7.9)	1.07 (0.89 - 1.28)	0.50
Acute myocardial infarction	150 (1.1)	95 (0.9)	55 (1.9)	0.91 (0.61 - 1.35)	0.64
Alzheimers disease and related disorders or senile dementia	450 (3.2)	228 (2.1)	222 (7.5)	1.51 (1.21 - 1.88)	2.0E-04 [a]
Anemia	1850 (13.2)	1213 (11.0)	637 (21.5)	1.19 (1.04 - 1.36)	0.01 [a]
Anxiety disorders	3382 (24.1)	2827 (25.6)	555 (18.8)	0.84 (0.74 - 0.96)	0.01 [a]
Asthma	913 (6.5)	756 (6.8)	157 (5.3)	1.07 (0.87 - 1.31)	0.52
Atrial fibrillation	1172 (8.4)	719 (6.5)	453 (15.3)	1.18 (1.01 - 1.37)	0.04
Bipolar disorder	871 (6.2)	683 (6.2)	188 (6.4)	1.31 (1.06 - 1.61)	0.01 [a]
Chronic kidney disease	2285 (16.3)	1441 (13.0)	844 (28.5)	1.57 (1.38 - 1.78)	<1.0E-08 [a]
Chronic obstructive pulmonary disease and bronchiectasis	2489 (17.8)	1539 (13.9)	950 (32.1)	1.71 (1.52 - 1.92)	<1.0E-08 [a]
Colorectal cancer	164 (1.2)	117 (1.1)	47 (1.6)	1.09 (0.74 - 1.59)	0.67
Depression	3113 (22.2)	2542 (23.0)	571 (19.3)	0.99 (0.86 - 1.14)	0.89
Diabetes	3931 (28.0)	2800 (25.3)	1131 (38.2)	1.22 (1.10 - 1.36)	3.0E-04 [a]
Epilepsy	175 (1.2)	117 (1.1)	58 (2.0)	1.44 (0.99 - 2.08)	0.05
Heart failure	1205 (8.6)	675 (6.1)	530 (17.9)	1.51 (1.29 - 1.77)	2.2E-07 [a]
Human immunodeficiency virus and/or acquired immunodeficiency syndrome HIV/AIDS	103 (0.7)	83 (0.8)	20 (0.7)	0.98 (0.56 - 1.70)	0.93
Hyperlipidemia	4665 (33.3)	3510 (31.7)	1155 (39.0)	0.84 (0.75 - 0.94)	0.003 [a]
Hypertension	6332 (45.2)	4642 (42.0)	1690 (57.1)	1.04 (0.93 - 1.16)	0.49
Ischemic heart disease	2066 (14.7)	1381 (12.5)	685 (23.1)	0.92 (0.81 - 1.05)	0.23
Leukemias and lymphomas	1336 (9.5)	993 (9.0)	343 (11.6)	1.58 (1.33 - 1.88)	1.7E-07 [a]
Liver disease cirrhosis and other liver conditions except viral hepatitis	785 (5.6)	577 (5.2)	208 (7.0)	1.29 (1.06 - 1.57)	0.01 [a]
Lung cancer	289 (2.1)	173 (1.6)	116 (3.9)	1.38 (1.05 - 1.80)	0.02 [a]
Mobility impairments	166 (1.2)	94 (0.8)	72 (2.4)	1.73 (1.20 - 2.51)	0.003 [a]
Multiple sclerosis and transverse myelitis	207 (1.5)	166 (1.5)	41 (1.4)	2.02 (1.31 - 3.11)	0.001 [a]
Peripheral vascular disease	558 (4.0)	317 (2.9)	241 (8.1)	1.26 (1.03 - 1.54)	0.03

© 2022 Vo AD et al. *JAMA Network Open.*

Pressure and chronic ulcers	235 (1.7)	117 (1.1)	118 (4.0)	1.68 (1.24 - 2.29)	9.0E-04 [a]
Prostate cancer	774 (5.5)	547 (4.9)	227 (7.7)	0.96 (0.81 - 1.16)	0.70
Schizophrenia and other psychotic disorders	327 (2.3)	236 (2.1)	91 (3.1)	1.41 (1.06 - 1.88)	0.02 [a]
Tobacco use	1523 (10.9)	1105 (10.0)	418 (14.1)	1.25 (1.07 - 1.45)	0.004 [a]
Viral hepatitis	271 (1.9)	194 (1.8)	77 (2.6)	0.74 (0.54 - 1.01)	0.06
Death from breakthrough	539 (3.8)	0 (0.0)	539 (18.2)		

[a] Meets criteria for statistical significance after adjustment for multiple comparisons, P<0.027 in this analysis. OR = odds ratio. CI = 95% confidence interval.

© 2022 Vo AD et al. *JAMA Network Open.*

eTable 7. Immunosuppressive Medications Before and/or After Vaccination

	Before initial vaccination only		After vaccination (before breakthrough) only		Both before and after vaccination (before breakthrough)	
	Non-severe	Severe	Non-severe	Severe	Non-severe	Severe
N	1687	263	6129	1767	2534	730
Sex (%)						
Male	1467 (87.0)	254 (96.6)	5320 (86.8)	1710 (96.8)	2182 (86.1)	693 (94.9)
Female	220 (13.0)	9 (3.4)	809 (13.2)	57 (3.2)	352 (13.9)	37 (5.1)
Age ranges (%)						
Less than 40	143 (8.5)	1 (0.4)	479 (7.8)	14 (0.8)	182 (7.2)	2 (0.3)
40-45	80 (4.7)	2 (0.8)	291 (4.7)	10 (0.6)	125 (4.9)	6 (0.8)
45-50	102 (6.0)	4 (1.5)	345 (5.6)	9 (0.5)	151 (6.0)	9 (1.2)
50-55	167 (9.9)	8 (3.0)	502 (8.2)	47 (2.7)	220 (8.7)	22 (3.0)
55-60	157 (9.3)	9 (3.4)	625 (10.2)	70 (4.0)	266 (10.5)	50 (6.8)
60-65	264 (15.6)	20 (7.6)	790 (12.9)	156 (8.8)	339 (13.4)	77 (10.5)
65-70	227 (13.5)	38 (14.4)	808 (13.2)	247 (14.0)	328 (12.9)	117 (16.0)
70-75	278 (16.5)	86 (32.7)	1121 (18.3)	490 (27.7)	517 (20.4)	203 (27.8)
75-80	158 (9.4)	45 (17.1)	715 (11.7)	350 (19.8)	277 (10.9)	127 (17.4)
80 or greater	111 (6.6)	50 (19.0)	453 (7.4)	374 (21.2)	129 (5.1)	117 (16.0)
Race (%)						
American Indian or Alaska Native	12 (0.7)	0 (0.0)	43 (0.7)	9 (0.5)	21 (0.8)	4 (0.5)
Asian	19 (1.1)	1 (0.4)	55 (0.9)	10 (0.6)	24 (0.9)	4 (0.5)
Black or African American	460 (27.3)	57 (21.7)	1538 (25.1)	379 (21.4)	579 (22.8)	178 (24.4)
Native Hawaiian or other Pacific Islander	22 (1.3)	1 (0.4)	58 (0.9)	11 (0.6)	23 (0.9)	7 (1.0)
White	1082 (64.1)	193 (73.4)	4093 (66.8)	1274 (72.1)	1710 (67.5)	503 (68.9)
Unknown	92 (5.5)	11 (4.2)	342 (5.6)	84 (4.8)	177 (7.0)	34 (4.7)
Ethnicity Hispanic/Latino (%)						
Hispanic or Latino	141 (8.4)	27 (10.3)	506 (8.3)	102 (5.8)	217 (8.6)	42 (5.8)
Not Hispanic or Latino	1486 (88.1)	231 (87.8)	5358 (87.4)	1619 (91.6)	2244 (88.6)	665 (91.1)
Unknown	60 (3.6)	5 (1.9)	265 (4.3)	46 (2.6)	73 (2.9)	23 (3.2)
US Region (%)						
Continental	287 (17.0)	35 (13.3)	1087 (17.7)	296 (16.8)	374 (14.8)	120 (16.4)
Midwest	335 (19.9)	67 (25.5)	1175 (19.2)	370 (20.9)	616 (24.3)	191 (26.2)
North Atlantic	367 (21.8)	56 (21.3)	1342 (21.9)	379 (21.4)	560 (22.1)	147 (20.1)
Pacific	240 (14.2)	40 (15.2)	862 (14.1)	253 (14.3)	430 (17.0)	109 (14.9)
Southeast	440 (26.1)	65 (24.7)	1573 (25.7)	466 (26.4)	530 (20.9)	163 (22.3)
Unknown	18 (1.1)	0 (0.0)	90 (1.5)	3 (0.2)	24 (0.9)	0 (0.0)

© 2022 Vo AD et al. *JAMA Network Open.*

Period of dominant variant (%)						
Pre-delta period	49 (2.9)	15 (5.7)	142 (2.3)	110 (6.2)	75 (3.0)	53 (7.3)
Delta period	399 (23.7)	119 (45.2)	1834 (29.9)	760 (43.0)	730 (28.8)	325 (44.5)
Omicron period	1239 (73.4)	129 (49.0)	4153 (67.8)	897 (50.8)	1729 (68.2)	352 (48.2)
Vaccine type (%)						
Janssen	166 (9.8)	29 (11.0)	530 (8.6)	153 (8.7)	195 (7.7)	42 (5.8)
Moderna	694 (41.1)	117 (44.5)	2763 (45.1)	731 (41.4)	1066 (42.1)	308 (42.2)
Pfizer	827 (49.0)	117 (44.5)	2836 (46.3)	883 (50.0)	1273 (50.2)	380 (52.1)
History of infection before vaccinated (%)	99 (5.9)	12 (4.6)	248 (4.0)	70 (4.0)	135 (5.3)	28 (3.8)
Months since full vax at breakthrough (%)						
4 or less	198 (11.7)	47 (17.9)	644 (10.5)	238 (13.5)	304 (12.0)	121 (16.6)
4-5	115 (6.8)	19 (7.2)	396 (6.5)	138 (7.8)	188 (7.4)	63 (8.6)
5-6	123 (7.3)	33 (12.5)	463 (7.6)	207 (11.7)	181 (7.1)	77 (10.5)
6-7	116 (6.9)	26 (9.9)	496 (8.1)	182 (10.3)	189 (7.5)	65 (8.9)
7-8	162 (9.6)	22 (8.4)	648 (10.6)	149 (8.4)	221 (8.7)	70 (9.6)
8-9	271 (16.1)	28 (10.6)	984 (16.1)	191 (10.8)	403 (15.9)	66 (9.0)
9-10	322 (19.1)	35 (13.3)	1103 (18.0)	255 (14.4)	520 (20.5)	99 (13.6)
10-11	281 (16.7)	39 (14.8)	925 (15.1)	265 (15.0)	366 (14.4)	114 (15.6)
11-12	86 (5.1)	11 (4.2)	414 (6.8)	130 (7.4)	149 (5.9)	51 (7.0)
12 or more	13 (0.8)	3 (1.1)	56 (0.9)	12 (0.7)	13 (0.5)	4 (0.5)
Months since boosted at breakthrough (%)						
Not boosted	1102 (65.3)	218 (82.9)	4290 (70.0)	1347 (76.2)	1449 (57.2)	486 (66.6)
1 or less	84 (5.0)	8 (3.0)	287 (4.7)	70 (4.0)	138 (5.4)	34 (4.7)
1-2	138 (8.2)	5 (1.9)	402 (6.6)	79 (4.5)	148 (5.8)	46 (6.3)
2-3	163 (9.7)	19 (7.2)	462 (7.5)	115 (6.5)	220 (8.7)	56 (7.7)
3-4	107 (6.3)	8 (3.0)	384 (6.3)	84 (4.8)	303 (12.0)	46 (6.3)
4-5	69 (4.1)	3 (1.1)	192 (3.1)	44 (2.5)	224 (8.8)	48 (6.6)
5-6	16 (0.9)	2 (0.8)	51 (0.8)	14 (0.8)	29 (1.1)	11 (1.5)
6 or more	8 (0.5)	0 (0.0)	61 (1.0)	14 (0.8)	23 (0.9)	3 (0.4)
Immune-suppressive medications before breakthrough (%)						
Before breakthrough Chemotherapy			455 (7.4)	207 (11.7)	241 (9.5)	103 (14.1)
Before breakthrough Cytokine-blocking			385 (6.3)	88 (5.0)	1016 (40.1)	112 (15.3)
Before breakthrough Glucocorticoids			4942 (80.6)	1394 (78.9)	781 (30.8)	427 (58.5)
Before breakthrough Leukocyte-inhibitory			495 (8.1)	208 (11.8)	943 (37.2)	278 (38.1)
Before breakthrough Lymphocyte-depleting			203 (3.3)	88 (5.0)	203 (8.0)	91 (12.5)
Immune-suppressive medications before initial vaccination (%)						
Before vaccine Chemotherapy	79 (4.7)	15 (5.7)			237 (9.4)	99 (13.6)

© 2022 Vo AD et al. *JAMA Network Open.*

Before vaccine Cytokine-blocking	110 (6.5)	9 (3.4)			986 (38.9)	106 (14.5)
Before vaccine Glucocorticoids	1363 (80.8)	218 (82.9)			761 (30.0)	412 (56.4)
Before vaccine Leukocyte-inhibitory	187 (11.1)	33 (12.5)			912 (36.0)	236 (32.3)
Before vaccine Lymphocyte-depleting	0 (0.0)	0 (0.0)			153 (6.0)	70 (9.6)
Persistent positive test (%)	16 (0.9)	3 (1.1)	36 (0.6)	21 (1.2)	18 (0.7)	10 (1.4)
BMI Class (%)						
Underweight	8 (0.5)	5 (1.9)	37 (0.6)	44 (2.5)	14 (0.6)	9 (1.2)
Normal	209 (12.4)	47 (17.9)	761 (12.4)	397 (22.5)	305 (12.0)	139 (19.0)
Overweight	499 (29.6)	73 (27.8)	1737 (28.3)	461 (26.1)	788 (31.1)	215 (29.5)
Obesity I	488 (28.9)	67 (25.5)	1728 (28.2)	424 (24.0)	741 (29.2)	179 (24.5)
Obesity II	273 (16.2)	38 (14.4)	1016 (16.6)	227 (12.8)	397 (15.7)	115 (15.8)
Severe Obesity	205 (12.2)	33 (12.5)	708 (11.6)	201 (11.4)	285 (11.2)	72 (9.9)
Unknown	5 (0.3)	0 (0.0)	142 (2.3)	13 (0.7)	4 (0.2)	1 (0.1)
Comorbidities (%)						
Acquired hypothyroidism	103 (6.1)	30 (11.4)	291 (4.7)	114 (6.5)	146 (5.8)	67 (9.2)
Acute myocardial infarction	22 (1.3)	8 (3.0)	47 (0.8)	27 (1.5)	19 (0.7)	16 (2.2)
Alzheimers disease and related disorders or senile dementia	45 (2.7)	31 (11.8)	121 (2.0)	118 (6.7)	43 (1.7)	54 (7.4)
Anemia	225 (13.3)	86 (32.7)	544 (8.9)	310 (17.5)	329 (13.0)	192 (26.3)
Anxiety disorders	510 (30.2)	75 (28.5)	1549 (25.3)	296 (16.8)	609 (24.0)	144 (19.7)
Asthma	137 (8.1)	23 (8.7)	385 (6.3)	74 (4.2)	215 (8.5)	51 (7.0)
Atrial fibrillation	136 (8.1)	61 (23.2)	366 (6.0)	239 (13.5)	152 (6.0)	121 (16.6)
Bipolar disorder	123 (7.3)	25 (9.5)	366 (6.0)	101 (5.7)	160 (6.3)	44 (6.0)
Chronic kidney disease	291 (17.2)	104 (39.5)	674 (11.0)	425 (24.1)	366 (14.4)	262 (35.9)
Chronic obstructive pulmonary disease and bronchiectasis	298 (17.7)	115 (43.7)	821 (13.4)	559 (31.6)	348 (13.7)	251 (34.4)
Colorectal cancer	21 (1.2)	6 (2.3)	56 (0.9)	26 (1.5)	34 (1.3)	9 (1.2)
Depression	461 (27.3)	74 (28.1)	1379 (22.5)	297 (16.8)	565 (22.3)	160 (21.9)
Diabetes	462 (27.4)	119 (45.2)	1472 (24.0)	623 (35.3)	638 (25.2)	300 (41.1)
Epilepsy	18 (1.1)	10 (3.8)	67 (1.1)	32 (1.8)	21 (0.8)	10 (1.4)
Heart failure	140 (8.3)	81 (30.8)	326 (5.3)	257 (14.5)	158 (6.2)	159 (21.8)
Human immunodeficiency virus and/or acquired immunodeficiency syndrome HIV/AIDS	12 (0.7)	1 (0.4)	47 (0.8)	15 (0.8)	13 (0.5)	4 (0.5)
Hyperlipidemia	592 (35.1)	131 (49.8)	1851 (30.2)	637 (36.0)	777 (30.7)	309 (42.3)
Hypertension	805 (47.7)	171 (65.0)	2363 (38.6)	934 (52.9)	1096 (43.3)	469 (64.2)
Ischemic heart disease	257 (15.2)	84 (31.9)	705 (11.5)	377 (21.3)	280 (11.0)	179 (24.5)
Leukemias and lymphomas	31 (1.8)	7 (2.7)	136 (2.2)	72 (4.1)	114 (4.5)	65 (8.9)
Liver disease cirrhosis and other liver conditions except viral hepatitis	104 (6.2)	27 (10.3)	281 (4.6)	106 (6.0)	143 (5.6)	56 (7.7)

© 2022 Vo AD et al. *JAMA Network Open.*

Lung cancer	32 (1.9)	16 (6.1)	78 (1.3)	65 (3.7)	47 (1.9)	32 (4.4)
Mobility impairments	19 (1.1)	4 (1.5)	48 (0.8)	43 (2.4)	22 (0.9)	21 (2.9)
Multiple sclerosis and transverse myelitis	8 (0.5)	0 (0.0)	71 (1.2)	23 (1.3)	83 (3.3)	17 (2.3)
Peripheral vascular disease	58 (3.4)	29 (11.0)	171 (2.8)	139 (7.9)	67 (2.6)	57 (7.8)
Pressure and chronic ulcers	30 (1.8)	12 (4.6)	49 (0.8)	70 (4.0)	26 (1.0)	28 (3.8)
Prostate cancer	88 (5.2)	25 (9.5)	260 (4.2)	129 (7.3)	134 (5.3)	57 (7.8)
Schizophrenia and other psychotic disorders	45 (2.7)	13 (4.9)	131 (2.1)	62 (3.5)	50 (2.0)	12 (1.6)
Tobacco use	218 (12.9)	49 (18.6)	576 (9.4)	255 (14.4)	247 (9.7)	96 (13.2)
Viral hepatitis	31 (1.8)	13 (4.9)	101 (1.6)	38 (2.2)	43 (1.7)	23 (3.2)
Medication classes at breakthrough (%)						
1			5807 (94.7)	1579 (89.4)	2062 (81.4)	559 (76.6)
2			293 (4.8)	162 (9.2)	429 (16.9)	150 (20.5)
3			29 (0.5)	22 (1.2)	43 (1.7)	20 (2.7)
4			0 (0.0)	4 (0.2)	0 (0.0)	1 (0.1)
Medication classes at vaccination (%)						
1	1637 (97.0)	251 (95.4)			1948 (76.9)	484 (66.3)
2	48 (2.8)	12 (4.6)			524 (20.7)	216 (29.6)
3	2 (0.1)	0 (0.0)			60 (2.4)	25 (3.4)
4	0 (0.0)	0 (0.0)			2 (0.1)	5 (0.7)
Death from breakthrough	0 (0.0)	36 (13.7)	0 (0.0)	287 (16.2)	0 (0.0)	157 (21.5)

© 2022 Vo AD et al. *JAMA Network Open.*

eTable 8. Subcohort of Immunocompromised Patients, Analysis by Number of Medication Classes

	Overall	Non-severe	Severe	Multivariable OR (CI) number of drug classes	P
N	14021	11062	2959		
Sex (%)					
Male	12505 (89.2)	9653 (87.3)	2852 (96.4)	1.0 (referent)	
Female	1516 (10.8)	1409 (12.7)	107 (3.6)	0.58 (0.47 - 0.73)	2.3E-06 [a]
Age ranges (%)					
Less than 40	848 (6.0)	829 (7.5)	19 (0.6)	0.66 (0.35 - 1.24)	0.20
40-45	524 (3.7)	506 (4.6)	18 (0.6)	0.97 (0.51 - 1.84)	0.93
45-50	636 (4.5)	613 (5.5)	23 (0.8)	1.0 (referent)	
50-55	1004 (7.2)	925 (8.4)	79 (2.7)	2.06 (1.27 - 3.35)	0.004 [a]
55-60	1228 (8.8)	1093 (9.9)	135 (4.6)	2.49 (1.56 - 3.96)	1.2E-04 [a]
60-65	1733 (12.4)	1467 (13.3)	266 (9.0)	3.53 (2.26 - 5.53)	3.5E-08 [a]
65-70	1874 (13.4)	1452 (13.1)	422 (14.3)	5.03 (3.22 - 7.85)	<1.0E-08 [a]
70-75	2955 (21.1)	2122 (19.2)	833 (28.2)	6.79 (4.38 - 10.6)	<1.0E-08 [a]
75-80	1841 (13.1)	1274 (11.5)	567 (19.2)	7.83 (5.01 - 12.2)	<1.0E-08 [a]
80 or greater	1378 (9.8)	781 (7.1)	597 (20.2)	12.9 (8.22 - 20.3)	<1.0E-08 [a]
Race (%)					
American Indian or Alaska Native	95 (0.7)	81 (0.7)	14 (0.5)	0.93 (0.50 - 1.72)	0.81
Asian	117 (0.8)	102 (0.9)	15 (0.5)	1.10 (0.60 - 2.01)	0.76
Black or African American	3339 (23.8)	2691 (24.3)	648 (21.9)	1.10 (0.98 - 1.24)	0.11
Native Hawaiian or other Pacific Islander	130 (0.9)	110 (1.0)	20 (0.7)	0.93 (0.56 - 1.57)	0.80
White	9554 (68.1)	7438 (67.2)	2116 (71.5)	1.0 (referent)	
Unknown	786 (5.6)	640 (5.8)	146 (4.9)	0.99 (0.79 - 1.24)	0.90
Ethnicity Hispanic/Latino (%)					
Hispanic or Latino	1094 (7.8)	912 (8.2)	182 (6.2)	1.07 (0.88 - 1.30)	0.49
Not Hispanic or Latino	12426 (88.6)	9731 (88.0)	2695 (91.1)	1.0 (referent)	
Unknown	501 (3.6)	419 (3.8)	82 (2.8)	0.94 (0.70 - 1.26)	0.68
US Region (%)					
Continental	2314 (16.5)	1835 (16.6)	479 (16.2)	1.03 (0.89 - 1.19)	0.67
Midwest	2973 (21.2)	2297 (20.8)	676 (22.8)	0.96 (0.84 - 1.09)	0.51
North Atlantic	3047 (21.7)	2419 (21.9)	628 (21.2)	0.88 (0.77 - 1.01)	0.07
Pacific	2096 (14.9)	1665 (15.1)	431 (14.6)	0.96 (0.82 - 1.11)	0.56
Southeast	3449 (24.6)	2708 (24.5)	741 (25.0)	1.0 (referent)	
Unknown	142 (1.0)	138 (1.2)	4 (0.1)	0.31 (0.11 - 0.87)	0.03
Period of dominant variant (%)					

© 2022 Vo AD et al. *JAMA Network Open.*

Pre-delta period	501 (3.6)	299 (2.7)	202 (6.8)	1.0 (referent)	
Delta period	4422 (31.5)	3146 (28.4)	1276 (43.1)	0.71 (0.55 - 0.92)	0.01 [a]
Omicron period	9098 (64.9)	7617 (68.9)	1481 (50.1)	0.42 (0.32 - 0.56)	<1.0E-08 [a]
Vaccine type (%)					
Janssen	1172 (8.4)	940 (8.5)	232 (7.8)	1.12 (0.93 - 1.34)	0.23
Moderna	6098 (43.5)	4860 (43.9)	1238 (41.8)	0.79 (0.71 - 0.87)	1.1E-06 [a]
Pfizer	6751 (48.1)	5262 (47.6)	1489 (50.3)	1.0 (referent)	
History of infection before vaccinated (%)	636 (4.5)	515 (4.7)	121 (4.1)	0.62 (0.48 - 0.78)	8.4E-05 [a]
Months since full vax at breakthrough (%)					
4 or less	1672 (11.9)	1225 (11.1)	447 (15.1)	1.0 (referent)	
4-5	975 (7.0)	743 (6.7)	232 (7.8)	1.06 (0.84 - 1.33)	0.62
5-6	1159 (8.3)	831 (7.5)	328 (11.1)	1.12 (0.90 - 1.40)	0.29
6-7	1140 (8.1)	845 (7.6)	295 (10.0)	1.05 (0.84 - 1.31)	0.67
7-8	1336 (9.5)	1083 (9.8)	253 (8.6)	0.94 (0.75 - 1.17)	0.57
8-9	2069 (14.8)	1759 (15.9)	310 (10.5)	0.98 (0.78 - 1.22)	0.84
9-10	2488 (17.7)	2070 (18.7)	418 (14.1)	1.11 (0.88 - 1.40)	0.37
10-11	2160 (15.4)	1709 (15.4)	451 (15.2)	1.33 (1.04 - 1.69)	0.02 [a]
11-12	915 (6.5)	710 (6.4)	205 (6.9)	1.42 (1.07 - 1.88)	0.02 [a]
12 or more	107 (0.8)	87 (0.8)	20 (0.7)	0.96 (0.54 - 1.70)	0.88
Months since boosted at breakthrough (%)					
Not boosted	9408 (67.1)	7234 (65.4)	2174 (73.5)	1.0 (referent)	
1 or less	663 (4.7)	541 (4.9)	122 (4.1)	0.80 (0.64 – 1.00)	0.05
1-2	878 (6.3)	734 (6.6)	144 (4.9)	0.64 (0.52 - 0.79)	3.9E-05 [a]
2-3	1107 (7.9)	903 (8.2)	204 (6.9)	0.70 (0.58 - 0.85)	2.2E-04 [a]
3-4	1040 (7.4)	883 (8.0)	157 (5.3)	0.51 (0.42 - 0.63)	<1.0E-08 [a]
4-5	670 (4.8)	563 (5.1)	107 (3.6)	0.54 (0.42 - 0.69)	1.2E-06 [a]
5-6	141 (1.0)	109 (1.0)	32 (1.1)	0.78 (0.50 - 1.22)	0.28
6 or more	114 (0.8)	95 (0.9)	19 (0.6)	0.75 (0.44 - 1.30)	0.30
Immune-suppressive medications before breakthrough (%)					
Before breakthrough Chemotherapy	1006 (7.2)	696 (6.3)	310 (10.5)		
Before breakthrough Cytokine-blocking	1601 (11.4)	1401 (12.7)	200 (6.8)		
Before breakthrough Glucocorticoids	7544 (53.8)	5723 (51.7)	1821 (61.5)		
Before breakthrough Leukocyte-inhibitory	1924 (13.7)	1438 (13.0)	486 (16.4)		
Before breakthrough Lymphocyte-depleting	585 (4.2)	406 (3.7)	179 (6.0)		
Immune-suppressive medications before initial vaccination (%)					
Before vaccine Chemotherapy	430 (3.1)	316 (2.9)	114 (3.9)		
Before vaccine Cytokine-blocking	1211 (8.6)	1096 (9.9)	115 (3.9)		
Before vaccine Glucocorticoids	2754 (19.6)	2124 (19.2)	630 (21.3)		

© 2022 Vo AD et al. *JAMA Network Open.*

Before vaccine Leukocyte-inhibitory	1368 (9.8)	1099 (9.9)	269 (9.1)		
Before vaccine Lymphocyte-depleting	223 (1.6)	153 (1.4)	70 (2.4)		
Persistent positive test (%)	111 (0.8)	74 (0.7)	37 (1.3)	1.17 (0.75 - 1.81)	0.48
BMI Class (%)					
Underweight	119 (0.8)	60 (0.5)	59 (2.0)	1.70 (1.12 - 2.56)	0.01 [a]
Normal	1994 (14.2)	1372 (12.4)	622 (21.0)	1.0 (referent)	
Overweight	4085 (29.1)	3271 (29.6)	814 (27.5)	0.65 (0.57 - 0.75)	<1.0E-08 [a]
Obesity I	3883 (27.7)	3155 (28.5)	728 (24.6)	0.69 (0.60 - 0.80)	5.0E-07 [a]
Obesity II	2204 (15.7)	1798 (16.3)	406 (13.7)	0.75 (0.64 - 0.89)	7.8E-04 [a]
Severe Obesity	1568 (11.2)	1252 (11.3)	316 (10.7)	0.91 (0.75 - 1.09)	0.30
Unknown	168 (1.2)	154 (1.4)	14 (0.5)	0.57 (0.32 - 1.05)	0.07
Comorbidities (%)					
Acquired hypothyroidism	834 (5.9)	600 (5.4)	234 (7.9)	1.05 (0.88 - 1.27)	0.57
Acute myocardial infarction	150 (1.1)	95 (0.9)	55 (1.9)	0.91 (0.61 - 1.35)	0.64
Alzheimers disease and related disorders or senile dementia	450 (3.2)	228 (2.1)	222 (7.5)	1.52 (1.22 - 1.89)	2.0E-04 [a]
Anemia	1850 (13.2)	1213 (11.0)	637 (21.5)	1.21 (1.06 - 1.38)	0.005 [a]
Anxiety disorders	3382 (24.1)	2827 (25.6)	555 (18.8)	0.85 (0.74 - 0.96)	0.01 [a]
Asthma	913 (6.5)	756 (6.8)	157 (5.3)	1.02 (0.83 - 1.25)	0.87
Atrial fibrillation	1172 (8.4)	719 (6.5)	453 (15.3)	1.19 (1.02 - 1.38)	0.03
Bipolar disorder	871 (6.2)	683 (6.2)	188 (6.4)	1.30 (1.06 - 1.60)	0.01 [a]
Chronic kidney disease	2285 (16.3)	1441 (13.0)	844 (28.5)	1.61 (1.42 - 1.83)	<1.0E-08 [a]
Chronic obstructive pulmonary disease and bronchiectasis	2489 (17.8)	1539 (13.9)	950 (32.1)	1.76 (1.57 - 1.97)	<1.0E-08 [a]
Colorectal cancer	164 (1.2)	117 (1.1)	47 (1.6)	1.12 (0.77 - 1.64)	0.55
Depression	3113 (22.2)	2542 (23.0)	571 (19.3)	0.99 (0.86 - 1.13)	0.88
Diabetes	3931 (28.0)	2800 (25.3)	1131 (38.2)	1.22 (1.10 - 1.36)	2.8E-04 [a]
Epilepsy	175 (1.2)	117 (1.1)	58 (2.0)	1.45 (1.00 - 2.10)	0.05
Heart failure	1205 (8.6)	675 (6.1)	530 (17.9)	1.53 (1.31 - 1.78)	1.1E-07 [a]
Human immunodeficiency virus and/or acquired immunodeficiency syndrome HIV/AIDS	103 (0.7)	83 (0.8)	20 (0.7)	0.95 (0.54 - 1.66)	0.85
Hyperlipidemia	4665 (33.3)	3510 (31.7)	1155 (39.0)	0.84 (0.75 - 0.94)	0.002 [a]
Hypertension	6332 (45.2)	4642 (42.0)	1690 (57.1)	1.04 (0.93 - 1.17)	0.44
Ischemic heart disease	2066 (14.7)	1381 (12.5)	685 (23.1)	0.93 (0.81 - 1.06)	0.29
Leukemias and lymphomas	1336 (9.5)	993 (9.0)	343 (11.6)	1.46 (1.23 - 1.74)	2.2E-05 [a]
Liver disease cirrhosis and other liver conditions except viral hepatitis	785 (5.6)	577 (5.2)	208 (7.0)	1.29 (1.06 - 1.56)	0.01 [a]
Lung cancer	289 (2.1)	173 (1.6)	116 (3.9)	1.45 (1.11 - 1.89)	0.006 [a]
Mobility impairments	166 (1.2)	94 (0.8)	72 (2.4)	1.75 (1.21 - 2.52)	0.003 [a]

© 2022 Vo AD et al. *JAMA Network Open.*

Multiple sclerosis and transverse myelitis	207 (1.5)	166 (1.5)	41 (1.4)	2.28 (1.51 - 3.42)	7.5E-05 [a]
Peripheral vascular disease	558 (4.0)	317 (2.9)	241 (8.1)	1.27 (1.04 - 1.56)	0.02 [a]
Pressure and chronic ulcers	235 (1.7)	117 (1.1)	118 (4.0)	1.69 (1.25 - 2.30)	7.7E-04 [a]
Prostate cancer	774 (5.5)	547 (4.9)	227 (7.7)	0.98 (0.82 - 1.17)	0.79
Schizophrenia and other psychotic disorders	327 (2.3)	236 (2.1)	91 (3.1)	1.44 (1.08 - 1.91)	0.01 [a]
Tobacco use	1523 (10.9)	1105 (10.0)	418 (14.1)	1.25 (1.08 - 1.45)	0.003 [a]
Viral hepatitis	271 (1.9)	194 (1.8)	77 (2.6)	0.73 (0.54 – 1.00)	0.05 [a]
Number of medication classes after vaccination (%)					
0	2961 (21.1)	2399 (21.7)	562 (19.0)	0.60 (0.51 - 0.69)	<1.0E-08 [a]
1	10007 (71.4)	7869 (71.1)	2138 (72.3)	1.0 (referent)	
2	934 (6.7)	722 (6.5)	212 (7.2)	1.92 (1.64 - 2.26)	<1.0E-08 [a]
3	114 (0.8)	72 (0.7)	42 (1.4)	2.53 (1.69 - 3.80)	7.5E-06 [a]
4	5 (0.0)	0 (0.0)	5 (0.2)	20.8 (3.93 - 110)	3.6E-04 [a]
Number of medication classes before vaccination (%)					
0	8817 (62.9)	6841 (61.8)	1966 (66.4)	1.16 (1.04 - 1.31)	0.009 [a]
1	4320 (30.8)	3585 (32.4)	735 (24.8)	1.0 (referent)	
2	790 (5.6)	572 (5.2)	228 (7.7)	1.16 (0.92 - 1.46)	0.21
3	87 (0.6)	62 (0.06)	25 (0.8)	1.09 (0.58 - 2.04)	0.80
4	7 (0.0)	2 (0.0)	5 (0.2)	ND	
Death from breakthrough	539 (3.8)	0 (0.0)	539 (18.2)		

[a] Meets criteria for statistical significance after adjustment for multiple comparisons, P<0.025 for this analysis. OR = odds ratio. CI = 95% confidence interval.

© 2022 Vo AD et al. *JAMA Network Open.*

eTable 9. Subcohort of Nonimmunocompromised Patients, Data and Analysis

	Overall	Non-severe	Severe	Multivariable OR (CI)	P
N	96739	89086	7653		
Sex (%)					
Male	85109 (88.0)	77736 (87.3)	7373 (96.3)	1.0 (referent)	
Female	11630 (12.0)	11350 (12.7)	280 (3.7)	0.70 (0.62 - 0.80)	1.0E-07 [a]
Age ranges (%)					
Less than 40	12266 (12.7)	12175 (13.7)	91 (1.2)	0.55 (0.41 - 0.75)	1.0E-04 [a]
40-45	5717 (5.9)	5647 (6.3)	70 (0.9)	0.86 (0.62 - 1.19)	0.36
45-50	5671 (5.9)	5587 (6.3)	84 (1.1)	1.0 (referent)	
50-55	8717 (9.0)	8519 (9.6)	198 (2.6)	1.47 (1.13 - 1.90)	0.004 [a]
55-60	9706 (10.0)	9365 (10.5)	341 (4.5)	2.15 (1.69 - 2.75)	<1.0E-08 [a]
60-65	11238 (11.6)	10615 (11.9)	623 (8.1)	3.14 (2.49 - 3.97)	<1.0E-08 [a]
65-70	10479 (10.8)	9561 (10.7)	918 (12.0)	4.70 (3.73 - 5.92)	<1.0E-08 [a]
70-75	15372 (15.9)	13581 (15.2)	1791 (23.4)	6.52 (5.19 - 8.18)	<1.0E-08 [a]
75-80	9721 (10.0)	8272 (9.3)	1449 (18.9)	8.97 (7.13 - 11.3)	<1.0E-08 [a]
80 or greater	7852 (8.1)	5764 (6.5)	2088 (27.3)	17.7 (14.1 - 22.4)	<1.0E-08 [a]
Race (%)					
American Indian or Alaska Native	795 (0.8)	721 (0.8)	74 (1.0)	1.40 (1.07 - 1.84)	0.01 [a]
Asian	1464 (1.5)	1423 (1.6)	41 (0.5)	0.76 (0.54 - 1.07)	0.12
Black or African American	23614 (24.4)	22099 (24.8)	1515 (19.8)	0.94 (0.88 - 1.01)	0.07
Native Hawaiian or other Pacific Islander	1012 (1.0)	956 (1.1)	56 (0.7)	0.87 (0.65 - 1.16)	0.34
White	62111 (64.2)	56580 (63.5)	5531 (72.3)	1.0 (referent)	
Unknown	7743 (8.0)	7307 (8.2)	436 (5.7)	0.89 (0.79 – 1.00)	0.05
Ethnicity Hispanic/Latino (%)					
Hispanic or Latino	10165 (10.5)	9593 (10.8)	572 (7.5)	1.01 (0.91 - 1.12)	0.84
Not Hispanic or Latino	80974 (83.7)	74210 (83.3)	6764 (88.4)	1.0 (referent)	
Unknown	5600 (5.8)	5283 (5.9)	317 (4.1)	1.05 (0.91 - 1.21)	0.50
US Region (%)					
Continental	15826 (16.4)	14408 (16.2)	1418 (18.5)	1.20 (1.11 - 1.31)	7.4E-06 [a]
Midwest	18916 (19.6)	17264 (19.4)	1652 (21.6)	1.00 (0.92 - 1.08)	0.98
North Atlantic	20743 (21.4)	19165 (21.5)	1578 (20.6)	0.96 (0.89 - 1.04)	0.29
Pacific	18136 (18.7)	16858 (18.9)	1278 (16.7)	0.97 (0.89 - 1.05)	0.46
Southeast	20955 (21.7)	19246 (21.6)	1709 (22.3)	1.0 (referent)	
Unknown	2163 (2.2)	2145 (2.4)	18 (0.2)	0.34 (0.21 - 0.55)	8.0E-06 [a]
Period of dominant variant (%)					
Pre-delta period	2387 (2.5)	1952 (2.2)	435 (5.7)	1.0 (referent)	
Delta period	25071 (25.9)	21616 (24.3)	3455 (45.1)	1.04 (0.89 - 1.20)	0.65

© 2022 Vo AD et al. *JAMA Network Open.*

Omicron period	69281 (71.6)	65518 (73.5)	3763 (49.2)	0.52 (0.44 - 0.61)	<1.0E-08 [a]
Vaccine type (%)					
Janssen	9894 (10.2)	9088 (10.2)	806 (10.5)	1.35 (1.24 - 1.48)	<1.0E-08 [a]
Moderna	39432 (40.8)	36429 (40.9)	3003 (39.2)	0.80 (0.75 - 0.84)	<1.0E-08 [a]
Pfizer	47413 (49.0)	43569 (48.9)	3844 (50.2)	1.0 (referent)	
History of infection before vaccinated (%)	3737 (3.9)	3444 (3.9)	293 (3.8)	0.71 (0.62 - 0.83)	5.4E-06 [a]
Months since full vax at breakthrough (%)					
4 or less	10825 (11.2)	9758 (11.0)	1067 (13.9)	1.0 (referent)	
4-5	6241 (6.5)	5612 (6.3)	629 (8.2)	1.04 (0.92 - 1.18)	0.52
5-6	6989 (7.2)	6208 (7.0)	781 (10.2)	0.97 (0.86 - 1.10)	0.63
6-7	7762 (8.0)	6918 (7.8)	844 (11.0)	1.09 (0.96 - 1.23)	0.17
7-8	10403 (10.8)	9582 (10.8)	821 (10.7)	1.18 (1.04 - 1.33)	0.008 [a]
8-9	17049 (17.6)	16114 (18.1)	935 (12.2)	1.19 (1.05 - 1.34)	0.006 [a]
9-10	17509 (18.1)	16500 (18.5)	1009 (13.2)	1.15 (1.01 - 1.31)	0.03 [a]
10-11	13571 (14.0)	12543 (14.1)	1028 (13.4)	1.33 (1.16 - 1.52)	5.3E-05 [a]
11-12	5604 (5.8)	5143 (5.8)	461 (6.0)	1.48 (1.26 - 1.74)	1.6E-06 [a]
12 or more	786 (0.8)	708 (0.8)	78 (1.0)	1.77 (1.32 - 2.36)	1.0E-04 [a]
Months since boosted at breakthrough (%)					
Not boosted	71631 (74.0)	65249 (73.2)	6382 (83.4)	1.0 (referent)	
1 or less	5092 (5.3)	4860 (5.5)	232 (3.0)	0.50 (0.43 - 0.57)	<1.0E-08 [a]
1-2	6854 (7.1)	6607 (7.4)	247 (3.2)	0.37 (0.32 - 0.43)	<1.0E-08 [a]
2-3	7443 (7.7)	7093 (8.0)	350 (4.6)	0.39 (0.34 - 0.44)	<1.0E-08 [a]
3-4	3593 (3.7)	3314 (3.7)	279 (3.6)	0.55 (0.48 - 0.64)	<1.0E-08 [a]
4-5	1079 (1.1)	980 (1.1)	99 (1.3)	0.64 (0.51 - 0.81)	1.6E-04 [a]
5-6	229 (0.2)	211 (0.2)	18 (0.2)	0.59 (0.35 - 0.99)	0.05
6 or more	818 (0.8)	772 (0.9)	46 (0.6)	0.77 (0.56 - 1.07)	0.12
Persistent positive test (%)	351 (0.4)	285 (0.3)	66 (0.9)	1.35 (1.00 - 1.82)	0.05
BMI Class (%)					
Underweight	558 (0.6)	436 (0.5)	122 (1.6)	1.47 (1.16 - 1.87)	0.001 [a]
Normal	12400 (12.8)	10927 (12.3)	1473 (19.2)	1.0 (referent)	
Overweight	28622 (29.6)	26521 (29.8)	2101 (27.5)	0.74 (0.68 - 0.80)	<1.0E-08 [a]
Obesity I	27273 (28.2)	25402 (28.5)	1871 (24.4)	0.81 (0.75 - 0.88)	7.2E-07 [a]
Obesity II	14658 (15.2)	13537 (15.2)	1121 (14.6)	1.01 (0.92 - 1.11)	0.81
Severe Obesity	9073 (9.4)	8258 (9.3)	815 (10.6)	1.36 (1.22 - 1.51)	1.0E-08 [a]
Unknown	4155 (4.3)	4005 (4.5)	150 (2.0)	0.97 (0.80 - 1.17)	0.75
Comorbidities (%)					
Acquired hypothyroidism	4131 (4.3)	3573 (4.0)	558 (7.3)	1.02 (0.92 - 1.14)	0.66
Acute myocardial infarction	637 (0.7)	478 (0.5)	159 (2.1)	1.15 (0.92 - 1.44)	0.21

© 2022 Vo AD et al. *JAMA Network Open.*

Alzheimers disease and related disorders or senile dementia	2600 (2.7)	1687 (1.9)	913 (11.9)	2.12 (1.92 - 2.35)	<1.0E-08 [a]
Anemia	6284 (6.5)	5055 (5.7)	1229 (16.1)	1.19 (1.09 - 1.29)	8.6E-05 [a]
Anxiety disorders	22899 (23.7)	21516 (24.2)	1383 (18.1)	0.92 (0.85 - 0.99)	0.03 [a]
Asthma	2605 (2.7)	2450 (2.8)	155 (2.0)	0.79 (0.66 - 0.95)	0.01 [a]
Atrial fibrillation	4627 (4.8)	3580 (4.0)	1047 (13.7)	1.18 (1.08 - 1.29)	2.4E-04 [a]
Bipolar disorder	5426 (5.6)	4971 (5.6)	455 (5.9)	1.22 (1.08 - 1.38)	0.001 [a]
Chronic kidney disease	9547 (9.9)	7630 (8.6)	1917 (25.0)	1.59 (1.47 - 1.71)	<1.0E-08 [a]
Chronic obstructive pulmonary disease and bronchiectasis	5848 (6.0)	4564 (5.1)	1284 (16.8)	1.62 (1.50 - 1.76)	<1.0E-08 [a]
Colorectal cancer	444 (0.5)	370 (0.4)	74 (1.0)	0.99 (0.75 - 1.31)	0.95
Depression	19429 (20.1)	17975 (20.2)	1454 (19.0)	1.12 (1.03 - 1.21)	0.005 [a]
Diabetes	22152 (22.9)	19119 (21.5)	3033 (39.6)	1.26 (1.19 - 1.34)	<1.0E-08 [a]
Epilepsy	906 (0.9)	753 (0.8)	153 (2.0)	1.50 (1.22 - 1.84)	1.0E-04 [a]
Heart failure	4239 (4.4)	3006 (3.4)	1233 (16.1)	1.84 (1.68 - 2.02)	<1.0E-08 [a]
Human immunodeficiency virus and/or acquired immunodeficiency syndrome HIV/AIDS	771 (0.8)	708 (0.8)	63 (0.8)	1.40 (1.06 - 1.86)	0.02 [a]
Hyperlipidemia	26846 (27.8)	24114 (27.1)	2732 (35.7)	0.78 (0.73 - 0.83)	<1.0E-08 [a]
Hypertension	32577 (33.7)	28548 (32.0)	4029 (52.6)	1.09 (1.03 - 1.17)	0.006 [a]
Ischemic heart disease	8941 (9.2)	7339 (8.2)	1602 (20.9)	1.00 (0.93 - 1.09)	0.91
Liver disease cirrhosis and other liver conditions except viral hepatitis	3408 (3.5)	2991 (3.4)	417 (5.4)	1.38 (1.22 - 1.56)	1.8E-07 [a]
Lung cancer	535 (0.6)	400 (0.4)	135 (1.8)	1.78 (1.43 - 2.22)	2.9E-07 [a]
Mobility impairments	864 (0.9)	634 (0.7)	230 (3.0)	2.00 (1.67 - 2.40)	<1.0E-08 [a]
Multiple sclerosis and transverse myelitis	247 (0.3)	196 (0.2)	51 (0.7)	3.33 (2.32 - 4.78)	<1.0E-08 [a]
Peripheral vascular disease	2539 (2.6)	1924 (2.2)	615 (8.0)	1.26 (1.12 - 1.41)	8.1E-05 [a]
Pressure and chronic ulcers	1112 (1.1)	755 (0.8)	357 (4.7)	1.53 (1.31 - 1.79)	9.9E-08 [a]
Prostate cancer	3041 (3.1)	2635 (3.0)	406 (5.3)	0.87 (0.77 - 0.98)	0.02 [a]
Schizophrenia and other psychotic disorders	2428 (2.5)	2084 (2.3)	344 (4.5)	1.79 (1.56 - 2.05)	<1.0E-08 [a]
Tobacco use	7799 (8.1)	7009 (7.9)	790 (10.3)	1.32 (1.21 - 1.45)	<1.0E-08 [a]
Viral hepatitis	1145 (1.2)	986 (1.1)	159 (2.1)	1.11 (0.91 - 1.35)	0.30
Death from breakthrough	1016 (1.1)	0 (0.0)	1016 (13.3)		

[a] Meets criteria for statistical significance after adjustment for multiple comparisons, P<0.035 for this analysis. OR = odds ratio. CI = 95% confidence interval.

© 2022 Vo AD et al. *JAMA Network Open.*

eTable 10. Subcohort of Patients Aged 50 or Older, Data and Analysis

	Overall	Non-severe	Severe	Multivariable OR (CI)	P
N	85098	74791	10307		
Sex (%)					
Male	78268 (92.0)	68307 (91.3)	9961 (96.6)	1.0 (referent)	
Female	6830 (8.0)	6484 (8.7)	346 (3.4)	0.72 (0.64 - 0.81)	5.0E-08 [a]
Age ranges (%)					
50-55	9721 (11.4)	9444 (12.6)	277 (2.7)		
55-60	10934 (12.8)	10458 (14.0)	476 (4.6)		
60-65	12971 (15.2)	12082 (16.2)	889 (8.6)		
65-70	12353 (14.5)	11013 (14.7)	1340 (13.0)		
70-75	18327 (21.5)	15703 (21.0)	2624 (25.5)		
75-80	11562 (13.6)	9546 (12.8)	2016 (19.6)		
80 or greater	9230 (10.8)	6545 (8.8)	2685 (26.1)		
Age (per 5 years)				1.42 (1.4 - 1.44)	<1.0E-08 [a]
Race (%)					
American Indian or Alaska Native	626 (0.7)	546 (0.7)	80 (0.8)	1.24 (0.96 - 1.61)	0.10
Asian	759 (0.9)	709 (0.9)	50 (0.5)	0.81 (0.59 - 1.12)	0.20
Black or African American	21252 (25.0)	19152 (25.6)	2100 (20.4)	0.98 (0.92 - 1.04)	0.52
Native Hawaiian or other Pacific Islander	798 (0.9)	724 (1.0)	74 (0.7)	0.89 (0.69 - 1.15)	0.38
White	56038 (65.9)	48587 (65.0)	7451 (72.3)	1.0 (referent)	
Unknown	5625 (6.6)	5073 (6.8)	552 (5.4)	0.90 (0.80 – 1.00)	0.05
Ethnicity Hispanic/Latino (%)					
Hispanic or Latino	6850 (8.0)	6140 (8.2)	710 (6.9)	1.02 (0.93 - 1.12)	0.70
Not Hispanic or Latino	74494 (87.5)	65272 (87.3)	9222 (89.5)	1.0 (referent)	
Unknown	3754 (4.4)	3379 (4.5)	375 (3.6)	1.01 (0.89 - 1.16)	0.86
US Region (%)					
Continental	13575 (16.0)	11751 (15.7)	1824 (17.7)	1.16 (1.08 - 1.25)	3.7E-05 [a]
Midwest	17725 (20.8)	15448 (20.7)	2277 (22.1)	0.99 (0.92 - 1.06)	0.70
North Atlantic	18426 (21.7)	16275 (21.8)	2151 (20.9)	0.93 (0.87 – 1.00)	0.05
Pacific	14739 (17.3)	13082 (17.5)	1657 (16.1)	0.97 (0.90 - 1.04)	0.41
Southeast	19480 (22.9)	17099 (22.9)	2381 (23.1)	1.0 (referent)	
Unknown	1153 (1.4)	1136 (1.5)	17 (0.2)	0.31 (0.19 - 0.50)	2.4E-06 [a]
Period of dominant variant (%)					
Pre-delta period	2640 (3.1)	2009 (2.7)	631 (6.1)	1.0 (referent)	
Delta period	24712 (29.0)	20096 (26.9)	4616 (44.8)	0.94 (0.83 - 1.08)	0.40
Omicron period	57746 (67.9)	52686 (70.4)	5060 (49.1)	0.49 (0.43 - 0.57)	<1.0E-08 [a]

© 2022 Vo AD et al. *JAMA Network Open.*

Vaccine type (%)					
Janssen	7503 (8.8)	6532 (8.7)	971 (9.4)	1.29 (1.19 - 1.41)	<1.0E-08 [a]
Moderna	36379 (42.7)	32230 (43.1)	4149 (40.3)	0.78 (0.75 - 0.82)	<1.0E-08 [a]
Pfizer	41216 (48.4)	36029 (48.2)	5187 (50.3)	1.0 (referent)	
History of infection before vaccinated (%)	3172 (3.7)	2772 (3.7)	400 (3.9)	0.69 (0.61 - 0.79)	1.8E-08 [a]
Months since full vax at breakthrough (%)					
4 or less	8486 (10.0)	7040 (9.4)	1446 (14.0)	1.0 (referent)	
4-5	5379 (6.3)	4551 (6.1)	828 (8.0)	1.07 (0.95 - 1.20)	0.28
5-6	6521 (7.7)	5438 (7.3)	1083 (10.5)	1.02 (0.92 - 1.14)	0.69
6-7	6700 (7.9)	5593 (7.5)	1107 (10.7)	1.09 (0.98 - 1.22)	0.12
7-8	7930 (9.3)	6895 (9.2)	1035 (10.0)	1.14 (1.02 - 1.27)	0.02 [a]
8-9	13320 (15.7)	12116 (16.2)	1204 (11.7)	1.17 (1.05 - 1.31)	0.006 [a]
9-10	16676 (19.6)	15281 (20.4)	1395 (13.5)	1.15 (1.02 - 1.29)	0.02 [a]
10-11	13907 (16.3)	12446 (16.6)	1461 (14.2)	1.32 (1.16 - 1.49)	1.2E-05 [a]
11-12	5455 (6.4)	4805 (6.4)	650 (6.3)	1.43 (1.24 - 1.65)	1.3E-06 [a]
12 or more	724 (0.9)	626 (0.8)	98 (1.0)	1.56 (1.20 - 2.02)	8.4E-04 [a]
Months since boosted at breakthrough (%)					
Not boosted	59003 (69.3)	50725 (67.8)	8278 (80.3)	1.0 (referent)	
1 or less	4655 (5.5)	4308 (5.8)	347 (3.4)	0.55 (0.49 - 0.62)	<1.0E-08 [a]
1-2	6656 (7.8)	6270 (8.4)	386 (3.7)	0.43 (0.38 - 0.48)	<1.0E-08 [a]
2-3	7825 (9.2)	7277 (9.7)	548 (5.3)	0.46 (0.41 - 0.51)	<1.0E-08 [a]
3-4	4343 (5.1)	3910 (5.2)	433 (4.2)	0.55 (0.48 - 0.62)	<1.0E-08 [a]
4-5	1624 (1.9)	1420 (1.9)	204 (2.0)	0.61 (0.51 - 0.72)	1.1E-08 [a]
5-6	330 (0.4)	281 (0.4)	49 (0.5)	0.67 (0.48 - 0.95)	0.02 [a]
6 or more	662 (0.8)	600 (0.8)	62 (0.6)	0.78 (0.59 - 1.04)	0.09
Immune-suppressive medications before breakthrough (%)					
Before breakthrough Chemotherapy	942 (1.1)	634 (0.8)	308 (3.0)	2.77 (2.32 - 3.32)	<1.0E-08 [a]
Before breakthrough Cytokine-blocking	1251 (1.5)	1055 (1.4)	196 (1.9)	1.66 (1.31 - 2.10)	2.7E-05 [a]
Before breakthrough Glucocorticoids	6467 (7.6)	4684 (6.3)	1783 (17.3)	2.34 (2.18 - 2.5)	<1.0E-08 [a]
Before breakthrough Leukocyte-inhibitory	1702 (2.0)	1223 (1.6)	479 (4.6)	2.9 (2.47 - 3.41)	<1.0E-08 [a]
Before breakthrough Lymphocyte-depleting	497 (0.6)	326 (0.4)	171 (1.7)	2.00 (1.51 - 2.66)	1.7E-06 [a]
Immune-suppressive medications before initial vaccination (%)					
Before vaccine Chemotherapy	391 (0.5)	278 (0.4)	113 (1.1)	0.86 (0.64 - 1.15)	0.31
Before vaccine Cytokine-blocking	922 (1.1)	808 (1.1)	114 (1.1)	0.63 (0.47 - 0.85)	0.002 [a]
Before vaccine Glucocorticoids	2377 (2.8)	1764 (2.4)	613 (5.9)	1.36 (1.21 - 1.53)	1.4E-07 [a]
Before vaccine Leukocyte-inhibitory	1209 (1.4)	945 (1.3)	264 (2.6)	0.78 (0.64 - 0.96)	0.02 [a]
Before vaccine Lymphocyte-depleting	193 (0.2)	125 (0.2)	68 (0.7)	1.15 (0.74 - 1.81)	0.53
Persistent positive test (%)	404 (0.5)	303 (0.4)	101 (1.0)	1.27 (0.98 - 1.63)	0.07

© 2022 Vo AD et al. *JAMA Network Open.*

BMI Class (%)					
Underweight	591 (0.7)	413 (0.6)	178 (1.7)	1.44 (1.17 - 1.78)	5.4E-04 [a]
Normal	11472 (13.5)	9409 (12.6)	2063 (20.0)	1.0 (referent)	
Overweight	25795 (30.3)	22941 (30.7)	2854 (27.7)	0.73 (0.68 - 0.79)	<1.0E-08 [a]
Obesity I	24261 (28.5)	21759 (29.1)	2502 (24.3)	0.80 (0.75 - 0.86)	<1.0E-08 [a]
Obesity II	12843 (15.1)	11357 (15.2)	1486 (14.4)	1 (0.92 - 1.08)	0.95
Severe Obesity	7870 (9.2)	6795 (9.1)	1075 (10.4)	1.29 (1.17 - 1.42)	1.3E-07 [a]
Unknown	2266 (2.7)	2117 (2.8)	149 (1.4)	0.92 (0.76 - 1.11)	0.38
Comorbidities (%)					
Acquired hypothyroidism	4393 (5.2)	3612 (4.8)	781 (7.6)	1.02 (0.92 - 1.12)	0.75
Acute myocardial infarction	749 (0.9)	542 (0.7)	207 (2.0)	1.05 (0.86 - 1.28)	0.63
Alzheimers disease and related disorders or senile dementia	3005 (3.5)	1876 (2.5)	1129 (11.0)	1.89 (1.72 - 2.07)	<1.0E-08 [a]
Anemia	7295 (8.6)	5458 (7.3)	1837 (17.8)	1.18 (1.10 - 1.27)	5.4E-06 [a]
Anxiety disorders	16675 (19.6)	14868 (19.9)	1807 (17.5)	0.88 (0.82 - 0.94)	2.3E-04 [a]
Asthma	2524 (3.0)	2233 (3.0)	291 (2.8)	0.87 (0.75 – 1.00)	0.05
Atrial fibrillation	5701 (6.7)	4203 (5.6)	1498 (14.5)	1.17 (1.09 - 1.27)	5.0E-05 [a]
Bipolar disorder	4288 (5.0)	3685 (4.9)	603 (5.9)	1.24 (1.11 - 1.38)	1.1E-04 [a]
Chronic kidney disease	11069 (13.0)	8356 (11.2)	2713 (26.3)	1.58 (1.48 - 1.68)	<1.0E-08 [a]
Chronic obstructive pulmonary disease and bronchiectasis	8150 (9.6)	5926 (7.9)	2224 (21.6)	1.67 (1.56 - 1.78)	<1.0E-08 [a]
Colorectal cancer	589 (0.7)	468 (0.6)	121 (1.2)	1.05 (0.83 - 1.31)	0.70
Depression	15229 (17.9)	13311 (17.8)	1918 (18.6)	1.09 (1.02 - 1.17)	0.01 [a]
Diabetes	24430 (28.7)	20324 (27.2)	4106 (39.8)	1.26 (1.19 - 1.33)	<1.0E-08 [a]
Epilepsy	891 (1.0)	687 (0.9)	204 (2.0)	1.49 (1.24 - 1.79)	2.1E-05 [a]
Heart failure	5322 (6.3)	3571 (4.8)	1751 (17.0)	1.72 (1.59 - 1.86)	<1.0E-08 [a]
Human immunodeficiency virus and/or acquired immunodeficiency syndrome HIV/AIDS	688 (0.8)	607 (0.8)	81 (0.8)	1.37 (1.06 - 1.77)	0.02 [a]
Hyperlipidemia	27594 (32.4)	23772 (31.8)	3822 (37.1)	0.80 (0.76 - 0.85)	<1.0E-08 [a]
Hypertension	35573 (41.8)	29939 (40.0)	5634 (54.7)	1.07 (1.01 - 1.13)	0.02 [a]
Ischemic heart disease	10764 (12.6)	8492 (11.4)	2272 (22.0)	0.98 (0.92 - 1.05)	0.63
Leukemias and lymphomas	1268 (1.5)	932 (1.2)	336 (3.3)	1.86 (1.60 - 2.17)	<1.0E-08 [a]
Liver disease cirrhosis and other liver conditions except viral hepatitis	3386 (4.0)	2792 (3.7)	594 (5.8)	1.34 (1.20 - 1.49)	7.9E-08 [a]
Lung cancer	820 (1.0)	569 (0.8)	251 (2.4)	1.67 (1.41 - 1.98)	<1.0E-08 [a]
Mobility impairments	939 (1.1)	648 (0.9)	291 (2.8)	1.89 (1.61 - 2.24)	<1.0E-08 [a]
Multiple sclerosis and transverse myelitis	326 (0.4)	244 (0.3)	82 (0.8)	2.80 (2.08 - 3.76)	<1.0E-08 [a]
Peripheral vascular disease	3050 (3.6)	2200 (2.9)	850 (8.2)	1.25 (1.13 - 1.38)	1.0E-05 [a]
Pressure and chronic ulcers	1306 (1.5)	837 (1.1)	469 (4.6)	1.58 (1.37 - 1.81)	<1.0E-08 [a]
Prostate cancer	3803 (4.5)	3170 (4.2)	633 (6.1)	0.89 (0.81 - 0.98)	0.02 [a]

© 2022 Vo AD et al. *JAMA Network Open.*

Schizophrenia and other psychotic disorders	2148 (2.5)	1730 (2.3)	418 (4.1)	1.75 (1.54 - 1.98)	<1.0E-08 [a]
Tobacco use	7134 (8.4)	5974 (8.0)	1160 (11.3)	1.33 (1.23 - 1.45)	<1.0E-08 [a]
Viral hepatitis	1310 (1.5)	1079 (1.4)	231 (2.2)	0.99 (0.84 - 1.18)	0.94
Death from breakthrough	1546 (1.8)	0 (0.0)	1546 (15.0)		

[a] Meets criteria for statistical significance after adjustment for multiple comparisons, P<0.035 for this analysis. OR = odds ratio. CI = 95% confidence interval.

© 2022 Vo AD et al. *JAMA Network Open.*

eTable 11. Subcohort of Nonimmunocompromised Patients Younger Than 65 Years With No Booster, Data and Analysis

	Overall	Non-severe	Severe	Multivariable OR (CI)	P
N	16996	16595	401		
Sex (%)					
Male	13626 (80.2)	13265 (79.9)	361 (90.0)	1.0 (referent)	
Female	3370 (19.8)	3330 (20.1)	40 (10.0)	0.62 (0.44 - 0.89)	0.008
Age ranges (%)					
Less than 40	5644 (33.2)	5602 (33.8)	42 (10.5)	0.78 (0.46 - 1.31)	0.35
40-45	2226 (13.1)	2194 (13.2)	32 (8.0)	1.25 (0.72 - 2.16)	0.43
45-50	1905 (11.2)	1882 (11.3)	23 (5.7)	1.0 (referent)	
50-55	2483 (14.6)	2429 (14.6)	54 (13.5)	1.59 (0.96 - 2.63)	0.07
55-60	2279 (13.4)	2183 (13.2)	96 (23.9)	2.99 (1.86 - 4.81)	5.6E-06 [a]
60-65	2240 (13.2)	2114 (12.7)	126 (31.4)	3.28 (2.05 - 5.25)	7.4E-07 [a]
65-70	219 (1.3)	191 (1.2)	28 (7.0)	7.41 (3.98 - 13.8)	<1.0E-08 [a]
Race (%)					
American Indian or Alaska Native	146 (0.9)	137 (0.8)	9 (2.2)	3.89 (1.87 - 8.09)	2.7E-04 [a]
Asian	319 (1.9)	313 (1.9)	6 (1.5)	1.77 (0.75 - 4.17)	0.19
Black or African American	4674 (27.5)	4556 (27.5)	118 (29.4)	1.15 (0.89 - 1.48)	0.28
Native Hawaiian or other Pacific Islander	196 (1.2)	193 (1.2)	3 (0.7)	0.91 (0.28 - 2.92)	0.87
White	9878 (58.1)	9638 (58.1)	240 (59.9)	1.0 (referent)	
Unknown	1783 (10.5)	1758 (10.6)	25 (6.2)	0.71 (0.45 - 1.14)	0.16
Ethnicity Hispanic/Latino (%)					
Hispanic or Latino	2203 (13.0)	2169 (13.1)	34 (8.5)	0.96 (0.65 - 1.42)	0.84
Not Hispanic or Latino	13288 (78.2)	12948 (78.0)	340 (84.8)	1.0 (referent)	
Unknown	1505 (8.9)	1478 (8.9)	27 (6.7)	1.45 (0.90 - 2.32)	0.13
US Region (%)					
Continental	2984 (17.6)	2893 (17.4)	91 (22.7)	1.30 (0.96 - 1.77)	0.09
Midwest	2842 (16.7)	2769 (16.7)	73 (18.2)	0.81 (0.58 - 1.13)	0.22
North Atlantic	3623 (21.3)	3565 (21.5)	58 (14.5)	0.63 (0.45 - 0.89)	0.009
Pacific	3352 (19.7)	3275 (19.7)	77 (19.2)	0.97 (0.70 - 1.35)	0.85
Southeast	3549 (20.9)	3450 (20.8)	99 (24.7)	1.0 (referent)	
Unknown	646 (3.8)	643 (3.9)	3 (0.7)	0.30 (0.09 - 0.96)	0.04
Period of dominant variant (%)					
Pre-delta period	90 (0.5)	83 (0.5)	7 (1.7)	1.0 (referent)	
Delta period	2841 (16.7)	2698 (16.3)	143 (35.7)	0.93 (0.38 - 2.27)	0.88
Omicron period	14065 (82.8)	13814 (83.2)	251 (62.6)	0.37 (0.15 - 0.90)	0.03
Vaccine type (%)					

© 2022 Vo AD et al. *JAMA Network Open.*

Janssen	4351 (25.6)	4181 (25.2)	170 (42.4)	1.70 (1.32 - 2.17)	2.9E-05 [a]
Moderna	4924 (29.0)	4832 (29.1)	92 (22.9)	0.96 (0.73 - 1.28)	0.79
Pfizer	7721 (45.4)	7582 (45.7)	139 (34.7)	1.0 (referent)	
History of infection before vaccinated (%)	833 (4.9)	823 (5.0)	10 (2.5)	0.44 (0.22 - 0.85)	0.01
Months since full vax at breakthrough (%)					
4 or less	4762 (28.0)	4621 (27.8)	141 (35.2)	1.0 (referent)	
4-5	1871 (11.0)	1818 (11.0)	53 (13.2)	1.00 (0.70 - 1.42)	0.98
5-6	1574 (9.3)	1538 (9.3)	36 (9.0)	0.81 (0.54 - 1.21)	0.31
6-7	2260 (13.3)	2210 (13.3)	50 (12.5)	1.08 (0.76 - 1.55)	0.66
7-8	3916 (23.0)	3849 (23.2)	67 (16.7)	1.02 (0.72 - 1.44)	0.90
8-9	2258 (13.3)	2214 (13.3)	44 (11.0)	1.22 (0.82 - 1.82)	0.32
9-10	341 (2.0)	331 (2.0)	10 (2.5)	1.29 (0.63 - 2.63)	0.48
10-11	14 (0.1)	14 (0.1)	0 (0.0)	ND	ND
11-12	0	0	0	ND	ND
12 or more	0	0	0	ND	ND
Persistent positive test (%)	36 (0.2)	35 (0.2)	1 (0.2)	0.29 (0.02 - 3.85)	0.35
BMI Class (%)					
Underweight	59 (0.3)	57 (0.3)	2 (0.5)	1.23 (0.27 - 5.63)	0.79
Normal	1912 (11.2)	1858 (11.2)	54 (13.5)	1.0 (referent)	
Overweight	4474 (26.3)	4400 (26.5)	74 (18.5)	0.64 (0.44 - 0.94)	0.02
Obesity I	4793 (28.2)	4679 (28.2)	114 (28.4)	0.93 (0.65 - 1.34)	0.70
Obesity II	2696 (15.9)	2617 (15.8)	79 (19.7)	1.15 (0.78 - 1.70)	0.49
Severe Obesity	1882 (11.1)	1818 (11.0)	64 (16.0)	1.19 (0.78 - 1.81)	0.41
Unknown	1180 (6.9)	1166 (7.0)	14 (3.5)	0.90 (0.47 - 1.74)	0.76
Comorbidities (%)					
Acquired hypothyroidism	592 (3.5)	563 (3.4)	29 (7.2)	1.32 (0.85 - 2.05)	0.22
Acute myocardial infarction	91 (0.5)	79 (0.5)	12 (3.0)	1.40 (0.63 - 3.12)	0.41
Alzheimers disease and related disorders or senile dementia	75 (0.4)	58 (0.3)	17 (4.2)	2.28 (1.16 - 4.49)	0.02
Anemia	796 (4.7)	750 (4.5)	46 (11.5)	0.97 (0.65 - 1.45)	0.89
Anxiety disorders	5909 (34.8)	5766 (34.7)	143 (35.7)	0.99 (0.77 - 1.28)	0.95
Asthma	647 (3.8)	626 (3.8)	21 (5.2)	1.23 (0.75 - 2.02)	0.41
Atrial fibrillation	233 (1.4)	214 (1.3)	19 (4.7)	0.80 (0.44 - 1.45)	0.46
Bipolar disorder	1411 (8.3)	1353 (8.2)	58 (14.5)	1.35 (0.96 - 1.90)	0.09
Chronic kidney disease	991 (5.8)	904 (5.4)	87 (21.7)	1.46 (1.05 - 2.03)	0.02
Chronic obstructive pulmonary disease and bronchiectasis	458 (2.7)	406 (2.4)	52 (13.0)	1.52 (1.04 - 2.23)	0.03
Colorectal cancer	38 (0.2)	34 (0.2)	4 (1.0)	1.38 (0.39 - 4.85)	0.62
Depression	4867 (28.6)	4712 (28.4)	155 (38.7)	1.38 (1.06 - 1.80)	0.02

© 2022 Vo AD et al. *JAMA Network Open.*

Diabetes	2280 (13.4)	2135 (12.9)	145 (36.2)	1.68 (1.28 - 2.22)	1.9E-04 [a]
Epilepsy	170 (1.0)	152 (0.9)	18 (4.5)	2.38 (1.31 - 4.31)	0.004 [a]
Heart failure	263 (1.5)	219 (1.3)	44 (11.0)	2.63 (1.66 - 4.17)	3.8E-05 [a]
Human immunodeficiency virus and/or acquired immunodeficiency syndrome HIV/AIDS	113 (0.7)	107 (0.6)	6 (1.5)	1.24 (0.46 - 3.32)	0.67
Hyperlipidemia	4322 (25.4)	4167 (25.1)	155 (38.7)	0.73 (0.57 - 0.94)	0.02
Hypertension	4270 (25.1)	4067 (24.5)	203 (50.6)	1.16 (0.90 - 1.51)	0.26
Ischemic heart disease	620 (3.6)	571 (3.4)	49 (12.2)	0.94 (0.62 - 1.43)	0.78
Liver disease cirrhosis and other liver conditions except viral hepatitis	691 (4.1)	645 (3.9)	46 (11.5)	1.55 (1.07 - 2.25)	0.02
Lung cancer	12 (0.1)	8 (0.0)	4 (1.0)	10.8 (2.69 - 43.5)	8.1E-04 [a]
Mobility impairments	82 (0.5)	74 (0.4)	8 (2.0)	0.80 (0.34 - 1.90)	0.62
Multiple sclerosis and transverse myelitis	34 (0.2)	31 (0.2)	3 (0.7)	5.17 (1.45 - 18.4)	0.01
Peripheral vascular disease	153 (0.9)	133 (0.8)	20 (5.0)	1.09 (0.59 - 2.01)	0.78
Pressure and chronic ulcers	87 (0.5)	69 (0.4)	18 (4.5)	2.81 (1.40 - 5.65)	0.004 [a]
Prostate cancer	116 (0.7)	109 (0.7)	7 (1.7)	1.17 (0.51 - 2.68)	0.71
Schizophrenia and other psychotic disorders	447 (2.6)	414 (2.5)	33 (8.2)	1.73 (1.11 - 2.70)	0.02
Tobacco use	2058 (12.1)	1956 (11.8)	102 (25.4)	1.42 (1.07 - 1.88)	0.01
Viral hepatitis	155 (0.9)	137 (0.8)	18 (4.5)	1.69 (0.93 - 3.09)	0.09
Death from breakthrough	42 (0.2)	0 (0.0)	42 (8.6)		

[a] Meets criteria for statistical significance after adjustment for multiple comparisons, $P<0.007$ for this analysis. OR = odds ratio. CI = 95% confidence interval.

© 2022 Vo AD et al. *JAMA Network Open.*

eTable 12. Subcohort of Nonimmunocompromised Patients Aged 65 Years or Older With No Booster, Data and Analysis

	Overall	Non-severe	Severe	Multivariable OR (CI)	P
N	17756	14428	3328		
Sex (%)					
Male	17210 (96.9)	13946 (96.7)	3264 (98.1)	1.0 (referent)	
Female	546 (3.1)	482 (3.3)	64 (1.9)	0.75 (0.57 – 1.00)	0.05
Age ranges (%)					
65-70	3815 (21.5)	3407 (23.6)	408 (12.3)	1.0 (referent)	
70-75	6389 (36.0)	5443 (37.7)	946 (28.4)	1.50 (1.32 - 1.71)	<1.0E-08 [a]
75-80	4017 (22.6)	3273 (22.7)	744 (22.4)	2.07 (1.79 - 2.38)	<1.0E-08 [a]
80 or greater	3535 (19.9)	2305 (16.0)	1230 (37.0)	4.58 (3.97 - 5.28)	<1.0E-08 [a]
Race (%)					
American Indian or Alaska Native	129 (0.7)	104 (0.7)	25 (0.8)	1.03 (0.63 - 1.66)	0.92
Asian	110 (0.6)	94 (0.7)	16 (0.5)	0.91 (0.51 - 1.63)	0.75
Black or African American	2744 (15.5)	2207 (15.3)	537 (16.1)	1.12 (1.00 - 1.27)	0.06
Native Hawaiian or other Pacific Islander	141 (0.8)	115 (0.8)	26 (0.8)	1.13 (0.71 - 1.81)	0.60
White	13512 (76.1)	10966 (76.0)	2546 (76.5)	1.0 (referent)	
Unknown	1120 (6.3)	942 (6.5)	178 (5.3)	0.83 (0.68 - 1.01)	0.06
Ethnicity Hispanic/Latino (%)					
Hispanic or Latino	1187 (6.7)	967 (6.7)	220 (6.6)	1.13 (0.95 - 1.34)	0.17
Not Hispanic or Latino	15826 (89.1)	12852 (89.1)	2974 (89.4)	1.0 (referent)	
Unknown	743 (4.2)	609 (4.2)	134 (4.0)	1.09 (0.86 - 1.38)	0.46
US Region (%)					
Continental	2972 (16.7)	2334 (16.2)	638 (19.2)	1.21 (1.07 - 1.37)	0.003 [a]
Midwest	3660 (20.6)	2964 (20.5)	696 (20.9)	1.03 (0.91 - 1.17)	0.59
North Atlantic	3628 (20.4)	2955 (20.5)	673 (20.2)	1.02 (0.90 - 1.16)	0.74
Pacific	3243 (18.3)	2700 (18.7)	543 (16.3)	0.94 (0.83 - 1.07)	0.37
Southeast	4188 (23.6)	3415 (23.7)	773 (23.2)	1.0 (referent)	
Unknown	65 (0.4)	60 (0.4)	5 (0.2)	0.51 (0.20 - 1.31)	0.16
Period of dominant variant (%)					
Pre-delta period	634 (3.6)	442 (3.1)	192 (5.8)	1.0 (referent)	
Delta period	5775 (32.5)	4311 (29.9)	1464 (44.0)	0.99 (0.78 - 1.26)	0.94
Omicron period	11347 (63.9)	9675 (67.1)	1672 (50.2)	0.51 (0.40 - 0.66)	2.1E-07 [a]
Vaccine type (%)					
Janssen	1684 (9.5)	1315 (9.1)	369 (11.1)	1.21 (1.05 - 1.40)	0.008 [a]
Moderna	8379 (47.2)	6963 (48.3)	1416 (42.5)	0.81 (0.75 - 0.89)	3.6E-06 [a]

© 2022 Vo AD et al. *JAMA Network Open.*

Pfizer	7693 (43.3)	6150 (42.6)	1543 (46.4)	1.0 (referent)	
History of infection before vaccinated (%)	552 (3.1)	451 (3.1)	101 (3.0)	0.58 (0.45 - 0.75)	3.3E-05 [a]
Months since full vax at breakthrough (%)					
4 or less	1975 (11.1)	1488 (10.3)	487 (14.6)	1.0 (referent)	
4-5	1188 (6.7)	930 (6.4)	258 (7.8)	1.03 (0.84 - 1.27)	0.75
5-6	1450 (8.2)	1106 (7.7)	344 (10.3)	1.13 (0.93 - 1.37)	0.23
6-7	1500 (8.4)	1132 (7.8)	368 (11.1)	1.19 (0.98 - 1.44)	0.08
7-8	1706 (9.6)	1355 (9.4)	351 (10.5)	1.09 (0.90 - 1.32)	0.37
8-9	2350 (13.2)	1929 (13.4)	421 (12.7)	1.16 (0.96 - 1.40)	0.12
9-10	3514 (19.8)	3034 (21.0)	480 (14.4)	1.03 (0.85 - 1.25)	0.76
10-11	3019 (17.0)	2570 (17.8)	449 (13.5)	1.11 (0.91 - 1.36)	0.31
11-12	958 (5.4)	807 (5.6)	151 (4.5)	1.06 (0.82 - 1.36)	0.67
12 or more	96 (0.5)	77 (0.5)	19 (0.6)	1.26 (0.72 - 2.18)	0.42
Persistent positive test (%)	101 (0.6)	78 (0.5)	23 (0.7)	0.88 (0.53 - 1.45)	0.61
BMI Class (%)					
Underweight	181 (1.0)	107 (0.7)	74 (2.2)	1.73 (1.23 - 2.44)	0.002 [a]
Normal	3028 (17.1)	2282 (15.8)	746 (22.4)	1.0 (referent)	
Overweight	5903 (33.2)	4941 (34.2)	962 (28.9)	0.72 (0.64 - 0.81)	3.7E-08 [a]
Obesity I	4731 (26.6)	3959 (27.4)	772 (23.2)	0.81 (0.72 - 0.92)	0.001 [a]
Obesity II	2266 (12.8)	1837 (12.7)	429 (12.9)	1.02 (0.88 - 1.19)	0.77
Severe Obesity	1229 (6.9)	946 (6.6)	283 (8.5)	1.38 (1.16 - 1.65)	3.3E-04 [a]
Unknown	418 (2.4)	356 (2.5)	62 (1.9)	0.91 (0.67 - 1.24)	0.56
Comorbidities (%)					
Acquired hypothyroidism	1087 (6.1)	816 (5.7)	271 (8.1)	1.01 (0.86 - 1.19)	0.92
Acute myocardial infarction	188 (1.1)	119 (0.8)	69 (2.1)	1.09 (0.76 - 1.56)	0.66
Alzheimers disease and related disorders or senile dementia	1057 (6.0)	593 (4.1)	464 (13.9)	2.01 (1.73 - 2.33)	<1.0E-08 [a]
Anemia	1717 (9.7)	1135 (7.9)	582 (17.5)	1.27 (1.11 - 1.45)	5.0E-04 [a]
Anxiety disorders	2637 (14.9)	2152 (14.9)	485 (14.6)	0.95 (0.83 - 1.08)	0.41
Asthma	316 (1.8)	266 (1.8)	50 (1.5)	0.68 (0.49 - 0.96)	0.03
Atrial fibrillation	1676 (9.4)	1161 (8.0)	515 (15.5)	1.15 (1.01 - 1.32)	0.04
Bipolar disorder	634 (3.6)	490 (3.4)	144 (4.3)	1.18 (0.94 - 1.48)	0.15
Chronic kidney disease	2600 (14.6)	1745 (12.1)	855 (25.7)	1.60 (1.42 - 1.80)	<1.0E-08 [a]
Chronic obstructive pulmonary disease and bronchiectasis	1922 (10.8)	1328 (9.2)	594 (17.8)	1.6 0(1.42 - 1.82)	<1.0E-08 [a]
Colorectal cancer	136 (0.8)	103 (0.7)	33 (1.0)	0.94 (0.61 - 1.46)	0.79
Depression	2345 (13.2)	1853 (12.8)	492 (14.8)	1.03 (0.90 - 1.19)	0.63
Diabetes	5514 (31.1)	4220 (29.2)	1294 (38.9)	1.21 (1.10 - 1.34)	1.1E-04 [a]
Epilepsy	172 (1.0)	121 (0.8)	51 (1.5)	1.23 (0.85 - 1.78)	0.27
Heart failure	1472 (8.3)	882 (6.1)	590 (17.7)	1.88 (1.63 - 2.16)	<1.0E-08 [a]

© 2022 Vo AD et al. *JAMA Network Open.*

Human immunodeficiency virus and/or acquired immunodeficiency syndrome HIV/AIDS	66 (0.4)	50 (0.3)	16 (0.5)	1.76 (0.96 - 3.24)	0.07
Hyperlipidemia	6055 (34.1)	4836 (33.5)	1219 (36.6)	0.78 (0.71 - 0.87)	4.3E-06 [a]
Hypertension	8009 (45.1)	6205 (43.0)	1804 (54.2)	1.10 (0.99 - 1.22)	0.07
Ischemic heart disease	2995 (16.9)	2247 (15.6)	748 (22.5)	0.91 (0.80 - 1.02)	0.10
Liver disease cirrhosis and other liver conditions except viral hepatitis	609 (3.4)	463 (3.2)	146 (4.4)	1.17 (0.94 - 1.45)	0.16
Lung cancer	173 (1.0)	114 (0.8)	59 (1.8)	1.64 (1.16 - 2.33)	0.005 [a]
Mobility impairments	223 (1.3)	137 (0.9)	86 (2.6)	1.70 (1.24 - 2.33)	0.001 [a]
Multiple sclerosis and transverse myelitis	35 (0.2)	21 (0.1)	14 (0.4)	3.45 (1.61 - 7.37)	0.001 [a]
Peripheral vascular disease	866 (4.9)	580 (4.0)	286 (8.6)	1.20 (1.01 - 1.43)	0.04
Pressure and chronic ulcers	359 (2.0)	205 (1.4)	154 (4.6)	1.31 (1.02 - 1.69)	0.04
Prostate cancer	926 (5.2)	740 (5.1)	186 (5.6)	0.80 (0.67 - 0.97)	0.02
Schizophrenia and other psychotic disorders	376 (2.1)	271 (1.9)	105 (3.2)	1.54 (1.19 - 1.99)	9.4E-04 [a]
Tobacco use	1215 (6.8)	923 (6.4)	292 (8.8)	1.28 (1.09 - 1.51)	0.003 [a]
Viral hepatitis	246 (1.4)	193 (1.3)	53 (1.6)	1.00 (0.70 - 1.42)	1.00
Death from breakthrough	786 (4.4)	0 (0.0)	786 (23.6)		

[a] Meets criteria for statistical significance after adjustment for multiple comparisons, P<0.015 in this analysis. OR = odds ratio. CI = 95% confidence interval.

© 2022 Vo AD et al. *JAMA Network Open.*

eFigure. Age Distributions of Male and Female Patients

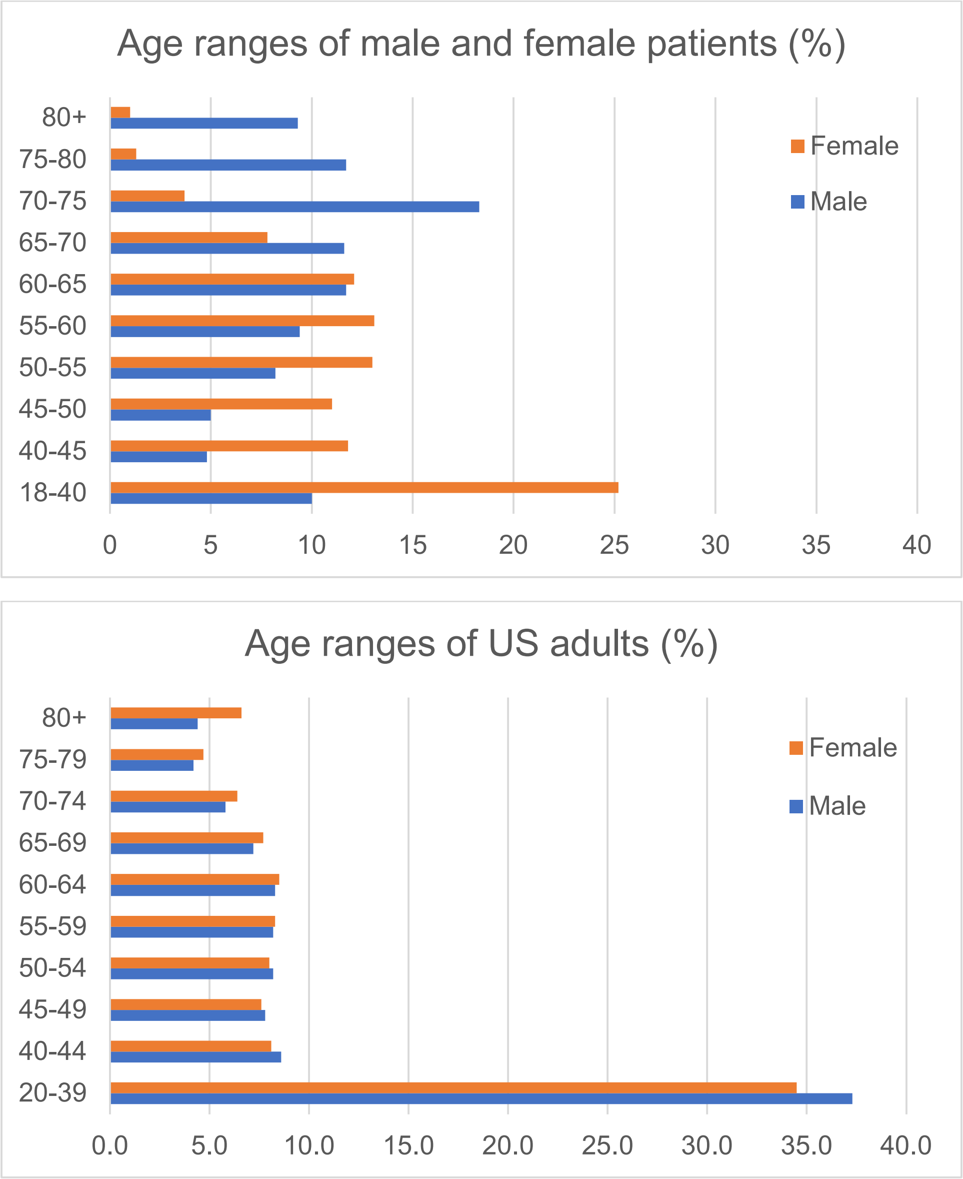

The X-axis indicates percentages within the group indicated. The male patients in the VA cohort are older than the female patients and than the general US male population.

© 2022 Vo AD et al. *JAMA Network Open.*

eTable 13. Subcohort of Female Patients, Data and Analysis

	Overall	Non-severe	Severe	Multivariable OR (CI)	P (multi)
N	13146	12759	387		
Age ranges (%)					
Less than 40	3314 (25.2)	3299 (25.9)	15 (3.9)	0.83 (0.38 - 1.83)	0.65
40-45	1553 (11.8)	1539 (12.1)	14 (3.6)	1.41 (0.63 - 3.12)	0.40
45-50	1449 (11.0)	1437 (11.3)	12 (3.1)	1.0 (referent)	
50-55	1706 (13.0)	1667 (13.1)	39 (10.1)	3.01 (1.53 - 5.91)	0.001 [a]
55-60	1721 (13.1)	1677 (13.1)	44 (11.4)	2.86 (1.46 - 5.60)	0.002 [a]
60-65	1593 (12.1)	1520 (11.9)	73 (18.9)	4.72 (2.46 - 9.05)	3.0E-06 [a]
65-70	1024 (7.8)	955 (7.5)	69 (17.8)	5.32 (2.73 - 10.4)	9.0E-07 [a]
70-75	489 (3.7)	428 (3.4)	61 (15.8)	9.40 (4.72 - 18.7)	<1.0E-08 [a]
75-80	171 (1.3)	145 (1.1)	26 (6.7)	14.9 (6.80 - 32.8)	<1.0E-08 [a]
80 or greater	126 (1.0)	92 (0.7)	34 (8.8)	43.5 (19.8 - 95.5)	<1.0E-08 [a]
Race (%)					
American Indian or Alaska Native	155 (1.2)	153 (1.2)	2 (0.5)	0.46 (0.10 - 2.04)	0.31
Asian	252 (1.9)	251 (2.0)	1 (0.3)	0.26 (0.03 - 1.97)	0.19
Black or African American	4537 (34.5)	4409 (34.6)	128 (33.1)	0.76 (0.58 - 1.01)	0.06
Native Hawaiian or other Pacific Islander	164 (1.2)	159 (1.2)	5 (1.3)	0.78 (0.27 - 2.29)	0.65
White	6713 (51.1)	6482 (50.8)	231 (59.7)	1.0 (referent)	
Unknown	1325 (10.1)	1305 (10.2)	20 (5.2)	0.68 (0.38 - 1.21)	0.19
Ethnicity Hispanic/Latino (%)					
Hispanic or Latino	1397 (10.6)	1377 (10.8)	20 (5.2)	0.64 (0.38 - 1.09)	0.10
Not Hispanic or Latino	10671 (81.2)	10318 (80.9)	353 (91.2)	1.0 (referent)	
Unknown	1078 (8.2)	1064 (8.3)	14 (3.6)	1.40 (0.73 - 2.72)	0.31
US Region (%)					
Continental	2340 (17.8)	2259 (17.7)	81 (20.9)	1.33 (0.96 - 1.86)	0.09
Midwest	2119 (16.1)	2053 (16.1)	66 (17.1)	0.85 (0.60 - 1.22)	0.39
North Atlantic	2754 (20.9)	2688 (21.1)	66 (17.1)	0.82 (0.57 - 1.16)	0.26
Pacific	2169 (16.5)	2106 (16.5)	63 (16.3)	1.11 (0.77 - 1.59)	0.59
Southeast	3220 (24.5)	3111 (24.4)	109 (28.2)	1.0 (referent)	
Unknown	544 (4.1)	542 (4.2)	2 (0.5)	0.33 (0.08 - 1.36)	0.13
Period of dominant variant (%)					
Pre-delta period				1.0 (referent)	
Delta period	2712 (20.6)	2556 (20.0)	156 (40.3)	0.89 (0.44 - 1.80)	0.75
Omicron period	10251 (78.0)	10036 (78.7)	215 (55.6)	0.41 (0.20 - 0.86)	0.02
Vaccine type (%)					

© 2022 Vo AD et al. *JAMA Network Open.*

Janssen	1317 (10.0)	1273 (10.0)	44 (11.4)	1.08 (0.74 - 1.56)	0.69
Moderna	4813 (36.6)	4703 (36.9)	110 (28.4)	0.58 (0.45 - 0.76)	5.4E-05 [a]
Pfizer	7016 (53.4)	6783 (53.2)	233 (60.2)	1.0 (referent)	
History of infection before vaccinated (%)	524 (4.0)	513 (4.0)	11 (2.8)	0.60 (0.30 - 1.19)	0.14
Months since full vax at breakthrough (%)					
4 or less	1694 (12.9)	1632 (12.8)	62 (16.0)	1.0 (referent)	
4-5	963 (7.3)	924 (7.2)	39 (10.1)	1.08 (0.66 - 1.77)	0.76
5-6	909 (6.9)	879 (6.9)	30 (7.8)	0.79 (0.46 - 1.34)	0.37
6-7	1036 (7.9)	1001 (7.8)	35 (9.0)	0.98 (0.58 - 1.65)	0.94
7-8	1616 (12.3)	1573 (12.3)	43 (11.1)	1.40 (0.87 - 2.28)	0.17
8-9	2671 (20.3)	2616 (20.5)	55 (14.2)	1.17 (0.72 - 1.89)	0.52
9-10	2191 (16.7)	2140 (16.8)	51 (13.2)	1.06 (0.63 - 1.78)	0.84
10-11	1278 (9.7)	1230 (9.6)	48 (12.4)	1.63 (0.93 - 2.85)	0.09
11-12	685 (5.2)	665 (5.2)	20 (5.2)	1.77 (0.89 - 3.53)	0.10
12 or more	103 (0.8)	99 (0.8)	4 (1.0)	2.58 (0.77 - 8.71)	0.13
Months since boosted at breakthrough (%)					
Not boosted	10214 (77.7)	9905 (77.6)	309 (79.8)	1.0 (referent)	
1 or less	656 (5.0)	640 (5.0)	16 (4.1)	0.71 (0.39 - 1.31)	0.28
1-2	790 (6.0)	783 (6.1)	7 (1.8)	0.17 (0.07 - 0.41)	1.1E-04 [a]
2-3	790 (6.0)	769 (6.0)	21 (5.4)	0.63 (0.37 - 1.07)	0.09
3-4	361 (2.7)	342 (2.7)	19 (4.9)	0.95 (0.53 - 1.71)	0.87
4-5	155 (1.2)	144 (1.1)	11 (2.8)	1.18 (0.57 - 2.44)	0.66
5-6	23 (0.2)	21 (0.2)	2 (0.5)	2.16 (0.40 - 11.8)	0.37
6 or more	157 (1.2)	155 (1.2)	2 (0.5)	0.53 (0.12 - 2.38)	0.40
Immune-suppressive medications before breakthrough (%)					
Before breakthrough Chemotherapy	49 (0.4)	42 (0.3)	7 (1.8)	4.55 (1.25 - 16.5)	0.02
Before breakthrough Cytokine-blocking	212 (1.6)	199 (1.6)	13 (3.4)	0.97 (0.38 - 2.50)	0.96
Before breakthrough Glucocorticoids	819 (6.2)	762 (6.0)	57 (14.7)	1.64 (1.16 - 2.33)	0.005 [a]
Before breakthrough Leukocyte-inhibitory	256 (1.9)	227 (1.8)	29 (7.5)	2.62 (1.24 - 5.53)	0.01
Before breakthrough Lymphocyte-depleting	69 (0.5)	59 (0.5)	10 (2.6)	2.37 (0.69 - 8.11)	0.17
Immune-suppressive medications before initial vaccination (%)					
Before vaccine Chemotherapy	27 (0.2)	24 (0.2)	3 (0.8)	0.44 (0.07 - 2.82)	0.39
Before vaccine Cytokine-blocking	176 (1.3)	166 (1.3)	10 (2.6)	0.96 (0.32 - 2.90)	0.94
Before vaccine Glucocorticoids	283 (2.2)	264 (2.1)	19 (4.9)	1.34 (0.76 - 2.35)	0.31
Before vaccine Leukocyte-inhibitory	202 (1.5)	181 (1.4)	21 (5.4)	1.13 (0.49 - 2.62)	0.77
Before vaccine Lymphocyte-depleting	28 (0.2)	24 (0.2)	4 (1.0)	0.31 (0.04 - 2.45)	0.27
Persistent positive test (%)	32 (0.2)	27 (0.2)	5 (1.3)	3.37 (1.07 - 10.7)	0.04

© 2022 Vo AD et al. *JAMA Network Open.*

BMI Class (%)					
Underweight	77 (0.6)	67 (0.5)	10 (2.6)	2.04 (0.86 - 4.82)	0.10
Normal	1752 (13.3)	1685 (13.2)	67 (17.3)	1.0 (referent)	
Overweight	3175 (24.2)	3111 (24.4)	64 (16.5)	0.49 (0.33 - 0.74)	05.4E-04 [a]
Obesity I	3364 (25.6)	3276 (25.7)	88 (22.7)	0.74 (0.51 - 1.08)	0.11
Obesity II	2216 (16.9)	2147 (16.8)	69 (17.8)	0.87 (0.58 - 1.29)	0.48
Severe Obesity	1688 (12.8)	1602 (12.6)	86 (22.2)	1.31 (0.88 - 1.95)	0.18
Unknown	874 (6.6)	871 (6.8)	3 (0.8)	0.27 (0.08 - 0.92)	0.04
Comorbidities (%)					
Acquired hypothyroidism	943 (7.2)	897 (7.0)	46 (11.9)	0.76 (0.52 - 1.12)	0.16
Acute myocardial infarction	32 (0.2)	27 (0.2)	5 (1.3)	0.92 (0.28 – 3.00)	0.89
Alzheimers disease and related disorders or senile dementia	95 (0.7)	76 (0.6)	19 (4.9)	1.57 (0.83 - 2.97)	0.17
Anemia	923 (7.0)	852 (6.7)	71 (18.3)	1.24 (0.85 - 1.79)	0.26
Anxiety disorders	4862 (37.0)	4729 (37.1)	133 (34.4)	1.02 (0.77 - 1.34)	0.92
Asthma	825 (6.3)	785 (6.2)	40 (10.3)	1.35 (0.91 - 1.99)	0.13
Atrial fibrillation	141 (1.1)	123 (1.0)	18 (4.7)	0.61 (0.33 - 1.16)	0.13
Bipolar disorder	1202 (9.1)	1152 (9.0)	50 (12.9)	1.53 (1.07 - 2.19)	0.02
Chronic kidney disease	625 (4.8)	530 (4.2)	95 (24.5)	2.14 (1.51 - 3.02)	1.7E-05 [a]
Chronic obstructive pulmonary disease and bronchiectasis	414 (3.1)	343 (2.7)	71 (18.3)	2.27 (1.57 - 3.29)	1.3E-05 [a]
Colorectal cancer	27 (0.2)	26 (0.2)	1 (0.3)	0.46 (0.05 - 3.89)	0.47
Depression	4579 (34.8)	4428 (34.7)	151 (39.0)	1.04 (0.79 - 1.37)	0.78
Diabetes	1624 (12.4)	1489 (11.7)	135 (34.9)	1.38 (1.03 - 1.85)	0.03
Epilepsy	113 (0.9)	102 (0.8)	11 (2.8)	2.10 (0.94 - 4.72)	0.07
Heart failure	166 (1.3)	130 (1.0)	36 (9.3)	2.04 (1.21 - 3.47)	0.008
HIV/AIDS	35 (0.3)	30 (0.2)	5 (1.3)	5.60 (1.85 - 16.9)	0.002 [a]
Hyperlipidemia	2329 (17.7)	2190 (17.2)	139 (35.9)	0.96 (0.72 - 1.27)	0.77
Hypertension	2749 (20.9)	2556 (20.0)	193 (49.9)	1.26 (0.95 - 1.66)	0.11
Ischemic heart disease	305 (2.3)	259 (2.0)	46 (11.9)	1.14 (0.71 - 1.82)	0.60
Leukemias and lymphomas	44 (0.3)	38 (0.3)	6 (1.6)	1.37 (0.44 - 4.25)	0.58
Liver disease: cirrhosis and other liver conditions except viral hepatitis	347 (2.6)	326 (2.6)	21 (5.4)	1.07 (0.62 - 1.83)	0.81
Lung cancer	33 (0.3)	27 (0.2)	6 (1.6)	1.68 (0.57 - 4.98)	0.34
Mobility impairments	59 (0.4)	49 (0.4)	10 (2.6)	2.61 (1.08 - 6.31)	0.03
Multiple sclerosis and transverse myelitis	121 (0.9)	111 (0.9)	10 (2.6)	3.01 (1.35 - 6.73)	0.007 [a]
Peripheral vascular disease	101 (0.8)	84 (0.7)	17 (4.4)	0.93 (0.47 - 1.83)	0.84
Pressure and chronic ulcers	42 (0.3)	30 (0.2)	12 (3.1)	2.33 (0.95 - 5.75)	0.07
Prostate cancer	1 (0.0)	1 (0.0)	0 (0.0)	ND	ND

© 2022 Vo AD et al. *JAMA Network Open.*

Schizophrenia and other psychotic disorders	307 (2.3)	284 (2.2)	23 (5.9)	1.61 (0.94 - 2.74)	0.08
Tobacco use	897 (6.8)	842 (6.6)	55 (14.2)	1.28 (0.88 - 1.86)	0.20
Viral hepatitis	57 (0.4)	52 (0.4)	5 (1.3)	1.11 (0.34 - 3.65)	0.86
Death from breakthrough	21 (0.2)	0 (0.0)	21 (5.4)		

[a] Meets criteria for statistical significance after adjustment for multiple comparisons, P<0.008 for this analysis. OR = odds ratio. CI = 95% confidence interval.

© 2022 Vo AD et al. *JAMA Network Open.*

eTable 14. Subcohort of Male Patients, Data and Analysis

	Overall	Non-severe	Severe	Multivariable OR (CI)	P (multi)
N	97614	87389	10225		
Age ranges (%)					
Less than 40	9800 (10.0)	9705 (11.1)	95 (0.9)	0.55 (0.41 - 0.74)	6.4E-05 [a]
40-45	4688 (4.8)	4614 (5.3)	74 (0.7)	0.83 (0.60 - 1.13)	0.24
45-50	4858 (5.0)	4763 (5.5)	95 (0.9)	1.0 (referent)	
50-55	8015 (8.2)	7777 (8.9)	238 (2.3)	1.41 (1.10 - 1.80)	0.007 [a]
55-60	9213 (9.4)	8781 (10.0)	432 (4.2)	2.04 (1.62 - 2.57)	<1.0E-08 [a]
60-65	11378 (11.7)	10562 (12.1)	816 (8.0)	2.85 (2.28 - 3.56)	<1.0E-08 [a]
65-70	11329 (11.6)	10058 (11.5)	1271 (12.4)	4.17 (3.35 - 5.19)	<1.0E-08 [a]
70-75	17838 (18.3)	15275 (17.5)	2563 (25.1)	5.56 (4.48 - 6.90)	<1.0E-08 [a]
75-80	11391 (11.7)	9401 (10.8)	1990 (19.5)	7.13 (5.72 - 8.87)	<1.0E-08 [a]
80 or greater	9104 (9.3)	6453 (7.4)	2651 (25.9)	12.5 (10.1 - 15.6)	<1.0E-08 [a]
Race (%)					
American Indian or Alaska Native	735 (0.8)	649 (0.7)	86 (0.8)	1.37 (1.05 - 1.79)	0.02 [a]
Asian	1329 (1.4)	1274 (1.5)	55 (0.5)	0.88 (0.64 - 1.20)	0.43
Black or African American	22416 (23.0)	20381 (23.3)	2035 (19.9)	0.98 (0.92 - 1.05)	0.63
Native Hawaiian or other Pacific Islander	978 (1.0)	907 (1.0)	71 (0.7)	0.81 (0.61 - 1.08)	0.15
White	64952 (66.5)	57536 (65.8)	7416 (72.5)	1.0 (referent)	
Unknown	7204 (7.4)	6642 (7.6)	562 (5.5)	0.92 (0.82 - 1.03)	0.16
Ethnicity Hispanic/Latino (%)					
Hispanic or Latino	9862 (10.1)	9128 (10.4)	734 (7.2)	1.06 (0.97 - 1.17)	0.21
Not Hispanic or Latino	82729 (84.8)	73623 (84.2)	9106 (89.1)	1.0 (referent)	
Unknown	5023 (5.1)	4638 (5.3)	385 (3.8)	1.07 (0.94 - 1.23)	0.32
US Region (%)					
Continental	15800 (16.2)	13984 (16.0)	1816 (17.8)	1.16 (1.07 - 1.25)	2.2E-04 [a]
Midwest	19770 (20.3)	17508 (20.0)	2262 (22.1)	0.98 (0.91 - 1.06)	0.67
North Atlantic	21036 (21.6)	18896 (21.6)	2140 (20.9)	0.94 (0.87 - 1.01)	0.09
Pacific	18063 (18.5)	16417 (18.8)	1646 (16.1)	0.95 (0.88 - 1.03)	0.24
Southeast	21184 (21.7)	18843 (21.6)	2341 (22.9)	1.0 (referent)	
Unknown	1761 (1.8)	1741 (2.0)	20 (0.2)	0.29 (0.18 - 0.48)	1.5E-06 [a]
Period of dominant variant (%)					
Pre-delta period				1.0 (referent)	
Delta period	26781 (27.4)	22206 (25.4)	4575 (44.7)	0.93 (0.81 - 1.07)	0.32
Omicron period	68128 (69.8)	63099 (72.2)	5029 (49.2)	0.48 (0.42 - 0.56)	<1.0E-08 [a]
Vaccine type (%)					

© 2022 Vo AD et al. *JAMA Network Open.*

Janssen	9749 (10.0)	8755 (10.0)	994 (9.7)	1.29 (1.19 - 1.41)	<1.0E-08 [a]
Moderna	40717 (41.7)	36586 (41.9)	4131 (40.4)	0.80 (0.76 - 0.84)	<1.0E-08 [a]
Pfizer	47148 (48.3)	42048 (48.1)	5100 (49.9)	1.0 (referent)	
History of infection before vaccinated (%)	3849 (3.9)	3446 (3.9)	403 (3.9)	0.76 (0.67 - 0.86)	3.1E-05 [a]
Months since full vax at breakthrough (%)					
4 or less	10803 (11.1)	9351 (10.7)	1452 (14.2)	1.0 (referent)	
4-5	6253 (6.4)	5431 (6.2)	822 (8.0)	1.07 (0.95 - 1.21)	0.26
5-6	7239 (7.4)	6160 (7.0)	1079 (10.6)	1.01 (0.90 - 1.13)	0.91
6-7	7866 (8.1)	6762 (7.7)	1104 (10.8)	1.07 (0.95 - 1.20)	0.24
7-8	10123 (10.4)	9092 (10.4)	1031 (10.1)	1.13 (1.01 - 1.27)	0.03 [a]
8-9	16447 (16.8)	15257 (17.5)	1190 (11.6)	1.13 (1.00 - 1.27)	0.04
9-10	17806 (18.2)	16430 (18.8)	1376 (13.5)	1.14 (1.01 - 1.28)	0.04
10-11	14453 (14.8)	13022 (14.9)	1431 (14.0)	1.31 (1.15 - 1.49)	4.6E-05 [a]
11-12	5834 (6.0)	5188 (5.9)	646 (6.3)	1.51 (1.30 - 1.75)	1.0E-07 [a]
12 or more	790 (0.8)	696 (0.8)	94 (0.9)	1.58 (1.19 - 2.08)	0.001 [a]
Months since boosted at breakthrough (%)					
Not boosted	70825 (72.6)	62578 (71.6)	8247 (80.7)	1.0 (referent)	
1 or less	5099 (5.2)	4761 (5.4)	338 (3.3)	0.57 (0.50 - 0.65)	<1.0E-08 [a]
1-2	6942 (7.1)	6558 (7.5)	384 (3.8)	0.46 (0.40 - 0.52)	<1.0E-08 [a]
2-3	7760 (7.9)	7227 (8.3)	533 (5.2)	0.46 (0.41 - 0.51)	<1.0E-08 [a]
3-4	4272 (4.4)	3855 (4.4)	417 (4.1)	0.55 (0.48 - 0.62)	<1.0E-08 [a]
4-5	1594 (1.6)	1399 (1.6)	195 (1.9)	0.59 (0.49 - 0.71)	<1.0E-08 [a]
5-6	347 (0.4)	299 (0.3)	48 (0.5)	0.72 (0.50 - 1.02)	0.07
6 or more	775 (0.8)	712 (0.8)	63 (0.6)	0.79 (0.59 - 1.07)	0.13
Immune-suppressive medications before breakthrough (%)					
Before breakthrough Chemotherapy	957 (1.0)	654 (0.7)	303 (3.0)	2.46 (2.03 - 2.99)	<1.0E-08 [a]
Before breakthrough Cytokine-blocking	1389 (1.4)	1202 (1.4)	187 (1.8)	1.62 (1.25 - 2.10)	2.3E-04 [a]
Before breakthrough Glucocorticoids	6725 (6.9)	4961 (5.7)	1764 (17.3)	2.36 (2.20 - 2.54)	<1.0E-08 [a]
Before breakthrough Leukocyte-inhibitory	1668 (1.7)	1211 (1.4)	457 (4.5)	2.48 (2.08 - 2.97)	<1.0E-08 [a]
Before breakthrough Lymphocyte-depleting	516 (0.5)	347 (0.4)	169 (1.7)	1.77 (1.30 - 2.40)	2.9E-04 [a]
Immune-suppressive medications before initial vaccination (%)				0.79 (0.57 - 1.10)	0.16
Before vaccine Chemotherapy	403 (0.4)	292 (0.3)	111 (1.1)	0.61 (0.44 - 0.84)	0.003 [a]
Before vaccine Cytokine-blocking	1035 (1.1)	930 (1.1)	105 (1.0)	1.31 (1.16 - 1.48)	2.0E-05 [a]
Before vaccine Glucocorticoids	2471 (2.5)	1860 (2.1)	611 (6.0)	0.76 (0.60 - 0.95)	0.02 [a]
Before vaccine Leukocyte-inhibitory	1166 (1.2)	918 (1.1)	248 (2.4)	1.24 (0.76 - 2.01)	0.39
Before vaccine Lymphocyte-depleting	195 (0.2)	129 (0.1)	66 (0.6)	1.47 (1.14 - 1.89)	0.003 [a]
Persistent positive test (%)	430 (0.4)	332 (0.4)	98 (1.0)	2.46 (2.03 - 2.99)	<1.0E-08 [a]

© 2022 Vo AD et al. *JAMA Network Open.*

BMI Class (%)					
Underweight	600 (0.6)	429 (0.5)	171 (1.7)	1.43 (1.14 - 1.79)	0.002 [a]
Normal	12642 (13.0)	10614 (12.1)	2028 (19.8)	1.0 (referent)	
Overweight	29532 (30.3)	26681 (30.5)	2851 (27.9)	0.76 (0.70 - 0.82)	<1.0E-08 [a]
Obesity I	27792 (28.5)	25281 (28.9)	2511 (24.6)	0.82 (0.76 - 0.89)	9.5E-07 [a]
Obesity II	14646 (15.0)	13188 (15.1)	1458 (14.3)	1.01 (0.92 - 1.10)	0.84
Severe Obesity	8953 (9.2)	7908 (9.0)	1045 (10.2)	1.30 (1.17 - 1.43)	3.3E-07 [a]
Unknown	3449 (3.5)	3288 (3.8)	161 (1.6)	0.95 (0.78 - 1.16)	0.62
Comorbidities (%)					
Acquired hypothyroidism	4022 (4.1)	3276 (3.7)	746 (7.3)	1.03 (0.93 - 1.14)	0.60
Acute myocardial infarction	755 (0.8)	546 (0.6)	209 (2.0)	1.14 (0.93 - 1.40)	0.21
Alzheimers disease and related disorders or senile dementia	2955 (3.0)	1839 (2.1)	1116 (10.9)	1.93 (1.75 - 2.13)	<1.0E-08 [a]
Anemia	7211 (7.4)	5416 (6.2)	1795 (17.6)	1.18 (1.09 - 1.27)	3.7E-05 [a]
Anxiety disorders	21419 (21.9)	19614 (22.4)	1805 (17.7)	0.90 (0.84 - 0.96)	0.003 [a]
Asthma	2693 (2.8)	2421 (2.8)	272 (2.7)	0.90 (0.78 - 1.05)	0.19
Atrial fibrillation	5658 (5.8)	4176 (4.8)	1482 (14.5)	1.21 (1.12 - 1.32)	3.5E-06 [a]
Bipolar disorder	5095 (5.2)	4502 (5.2)	593 (5.8)	1.23 (1.10 - 1.37)	3.4E-04 [a]
Chronic kidney disease	11207 (11.5)	8541 (9.8)	2666 (26.1)	1.52 (1.42 - 1.63)	<1.0E-08 [a]
Chronic obstructive pulmonary disease and bronchiectasis	7923 (8.1)	5760 (6.6)	2163 (21.2)	1.72 (1.60 - 1.84)	<1.0E-08 [a]
Colorectal cancer	581 (0.6)	461 (0.5)	120 (1.2)	1.01 (0.79 - 1.29)	0.94
Depression	17963 (18.4)	16089 (18.4)	1874 (18.3)	1.10 (1.02 - 1.18)	0.02 [a]
Diabetes	24459 (25.1)	20430 (23.4)	4029 (39.4)	1.24 (1.17 - 1.31)	<1.0E-08 [a]
Epilepsy	968 (1.0)	768 (0.9)	200 (2.0)	1.48 (1.22 - 1.79)	6.9E-05
Heart failure	5278 (5.4)	3551 (4.1)	1727 (16.9)	1.70 (1.56 - 1.85)	<1.0E-08 [a]
HIV/AIDS	839 (0.9)	761 (0.9)	78 (0.8)	1.22 (0.93 - 1.60)	0.16
Hyperlipidemia	29182 (29.9)	25434 (29.1)	3748 (36.7)	0.81 (0.76 - 0.86)	<1.0E-08 [a]
Hypertension	36160 (37.0)	30634 (35.1)	5526 (54.0)	1.07 (1.01 - 1.14)	0.02 [a]
Ischemic heart disease	10702 (11.0)	8461 (9.7)	2241 (21.9)	0.97 (0.90 - 1.04)	0.37
Leukemias and lymphomas	1292 (1.3)	955 (1.1)	337 (3.3)	1.60 (1.36 - 1.90)	3.7E-08 [a]
Liver disease: cirrhosis and other liver conditions except viral hepatitis	3846 (3.9)	3242 (3.7)	604 (5.9)	1.30 (1.16 - 1.46)	4.3E-06 [a]
Lung cancer	791 (0.8)	546 (0.6)	245 (2.4)	1.53 (1.27 - 1.84)	8.0E-06 [a]
Mobility impairments	971 (1.0)	679 (0.8)	292 (2.9)	1.97 (1.66 - 2.34)	<1.0E-08 [a]
Multiple sclerosis and transverse myelitis	333 (0.3)	251 (0.3)	82 (0.8)	3.13 (2.30 - 4.26)	<1.0E-08 [a]
Peripheral vascular disease	2996 (3.1)	2157 (2.5)	839 (8.2)	1.22 (1.10 - 1.36)	2.0E-04 [a]
Pressure and chronic ulcers	1305 (1.3)	842 (1.0)	463 (4.5)	1.52 (1.31 - 1.76)	3.6E-08 [a]
Prostate cancer	3814 (3.9)	3181 (3.6)	633 (6.2)	0.92 (0.83 - 1.02)	0.11

© 2022 Vo AD et al. *JAMA Network Open.*

Schizophrenia and other psychotic disorders	2448 (2.5)	2036 (2.3)	412 (4.0)	1.68 (1.47 - 1.92)	<1.0E-08 [a]
Tobacco use	8425 (8.6)	7272 (8.3)	1153 (11.3)	1.37 (1.26 - 1.49)	<1.0E-08 [a]
Viral hepatitis	1359 (1.4)	1128 (1.3)	231 (2.3)	0.98 (0.82 - 1.17)	0.79
Death from breakthrough	1534 (1.6)	0 (0.0)	1534 (15.0)		

[a] Meets criteria for statistical significance after adjustment for multiple comparisons, P<0.033 for this analysis. OR = odds ratio. CI = 95% confidence interval.

© 2022 Vo AD et al. *JAMA Network Open.*

eTable 15. Subcohorts of Female and Male, Comparison of Analyses

	Female			Male		
	Non-severe	Severe	Multivariable OR (CI)	Non-severe	Severe	Multivariable OR (CI)
N	12759	387		87389	10225	
Age ranges (%)						
Less than 40	3299 (25.9)	15 (3.9)	0.83 (0.38 - 1.83)	9705 (11.1)	95 (0.9)	0.55 (0.41 - 0.74)
40-45	1539 (12.1)	14 (3.6)	1.41 (0.63 - 3.12)	4614 (5.3)	74 (0.7)	0.83 (0.60 - 1.13)
45-50	1437 (11.3)	12 (3.1)	1.0 (referent)	4763 (5.5)	95 (0.9)	1.0 (referent)
50-55	1667 (13.1)	39 (10.1)	3.01 (1.53 - 5.91)	7777 (8.9)	238 (2.3)	1.41 (1.10 - 1.80)
55-60	1677 (13.1)	44 (11.4)	2.86 (1.46 - 5.60)	8781 (10.0)	432 (4.2)	2.04 (1.62 - 2.57)
60-65	1520 (11.9)	73 (18.9)	4.72 (2.46 - 9.05)	10562 (12.1)	816 (8.0)	2.85 (2.28 - 3.56)
65-70	955 (7.5)	69 (17.8)	5.32 (2.73 - 10.4)	10058 (11.5)	1271 (12.4)	4.17 (3.35 - 5.19)
70-75	428 (3.4)	61 (15.8)	9.40 (4.72 - 18.7)	15275 (17.5)	2563 (25.1)	5.56 (4.48 - 6.90)
75-80	145 (1.1)	26 (6.7)	14.9 (6.80 - 32.8)	9401 (10.8)	1990 (19.5)	7.13 (5.72 - 8.87)
80 or greater	92 (0.7)	34 (8.8)	43.5 (19.8 - 95.5)	6453 (7.4)	2651 (25.9)	12.5 (10.1 - 15.6)
Race (%)						
American Indian or Alaska Native	153 (1.2)	2 (0.5)	0.46 (0.10 - 2.04)	649 (0.7)	86 (0.8)	1.37 (1.05 - 1.79)
Asian	251 (2.0)	1 (0.3)	0.26 (0.03 - 1.97)	1274 (1.5)	55 (0.5)	0.88 (0.64 - 1.20)
Black or African American	4409 (34.6)	128 (33.1)	0.76 (0.58 - 1.01)	20381 (23.3)	2035 (19.9)	0.98 (0.92 - 1.05)
Native Hawaiian or other Pacific Islander	159 (1.2)	5 (1.3)	0.78 (0.27 - 2.29)	907 (1.0)	71 (0.7)	0.81 (0.61 - 1.08)
White	6482 (50.8)	231 (59.7)	1.0 (referent)	57536 (65.8)	7416 (72.5)	1.0 (referent)
Unknown	1305 (10.2)	20 (5.2)	0.68 (0.38 - 1.21)	6642 (7.6)	562 (5.5)	0.92 (0.82 - 1.03)
Ethnicity Hispanic/Latino (%)						
Hispanic or Latino	1377 (10.8)	20 (5.2)	0.64 (0.38 - 1.09)	9128 (10.4)	734 (7.2)	1.06 (0.97 - 1.17)
Not Hispanic or Latino	10318 (80.9)	353 (91.2)	1.0 (referent)	73623 (84.2)	9106 (89.1)	1.0 (referent)
Unknown	1064 (8.3)	14 (3.6)	1.40 (0.73 - 2.72)	4638 (5.3)	385 (3.8)	1.07 (0.94 - 1.23)
US Region (%)						
Continental	2259 (17.7)	81 (20.9)	1.33 (0.96 - 1.86)	13984 (16.0)	1816 (17.8)	1.16 (1.07 - 1.25)
Midwest	2053 (16.1)	66 (17.1)	0.85 (0.60 - 1.22)	17508 (20.0)	2262 (22.1)	0.98 (0.91 - 1.06)
North Atlantic	2688 (21.1)	66 (17.1)	0.82 (0.57 - 1.16)	18896 (21.6)	2140 (20.9)	0.94 (0.87 - 1.01)
Pacific	2106 (16.5)	63 (16.3)	1.11 (0.77 - 1.59)	16417 (18.8)	1646 (16.1)	0.95 (0.88 - 1.03)
Southeast	3111 (24.4)	109 (28.2)	1.0 (referent)	18843 (21.6)	2341 (22.9)	1.0 (referent)
Unknown	542 (4.2)	2 (0.5)	0.33 (0.08 - 1.36)	1741 (2.0)	20 (0.2)	0.29 (0.18 - 0.48)
Period of dominant variant (%)						
Pre-delta period			1.0 (referent)			1.0 (referent)
Delta period	2556 (20.0)	156 (40.3)	0.89 (0.44 - 1.80)	22206 (25.4)	4575 (44.7)	0.93 (0.81 - 1.07)
Omicron period	10036 (78.7)	215 (55.6)	0.41 (0.20 - 0.86)	63099 (72.2)	5029 (49.2)	0.48 (0.42 - 0.56)

© 2022 Vo AD et al. *JAMA Network Open.*

Vaccine type (%)						
Janssen	1273 (10.0)	44 (11.4)	1.08 (0.74 - 1.56)	8755 (10.0)	994 (9.7)	1.29 (1.19 - 1.41)
Moderna	4703 (36.9)	110 (28.4)	0.58 (0.45 - 0.76)	36586 (41.9)	4131 (40.4)	0.80 (0.76 - 0.84)
Pfizer	6783 (53.2)	233 (60.2)	1.0 (referent)	42048 (48.1)	5100 (49.9)	1.0 (referent)
History of infection before vaccinated (%)	513 (4.0)	11 (2.8)	0.60 (0.30 - 1.19)	3446 (3.9)	403 (3.9)	0.76 (0.67 - 0.86)
Months since full vax at breakthrough (%)						
4 or less	1632 (12.8)	62 (16.0)	1.0 (referent)	9351 (10.7)	1452 (14.2)	1.0 (referent)
4-5	924 (7.2)	39 (10.1)	1.08 (0.66 - 1.77)	5431 (6.2)	822 (8.0)	1.07 (0.95 - 1.21)
5-6	879 (6.9)	30 (7.8)	0.79 (0.46 - 1.34)	6160 (7.0)	1079 (10.6)	1.01 (0.90 - 1.13)
6-7	1001 (7.8)	35 (9.0)	0.98 (0.58 - 1.65)	6762 (7.7)	1104 (10.8)	1.07 (0.95 - 1.20)
7-8	1573 (12.3)	43 (11.1)	1.40 (0.87 - 2.28)	9092 (10.4)	1031 (10.1)	1.13 (1.01 - 1.27)
8-9	2616 (20.5)	55 (14.2)	1.17 (0.72 - 1.89)	15257 (17.5)	1190 (11.6)	1.13 (1.00 - 1.27)
9-10	2140 (16.8)	51 (13.2)	1.06 (0.63 - 1.78)	16430 (18.8)	1376 (13.5)	1.14 (1.01 - 1.28)
10-11	1230 (9.6)	48 (12.4)	1.63 (0.93 - 2.85)	13022 (14.9)	1431 (14.0)	1.31 (1.15 - 1.49)
11-12	665 (5.2)	20 (5.2)	1.77 (0.89 - 3.53)	5188 (5.9)	646 (6.3)	1.51 (1.30 - 1.75)
12 or more	99 (0.8)	4 (1.0)	2.58 (0.77 - 8.71)	696 (0.8)	94 (0.9)	1.58 (1.19 - 2.08)
Months since boosted at breakthrough (%)						
Not boosted	9905 (77.6)	309 (79.8)	1.0 (referent)	62578 (71.6)	8247 (80.7)	1.0 (referent)
1 or less	640 (5.0)	16 (4.1)	0.71 (0.39 - 1.31)	4761 (5.4)	338 (3.3)	0.57 (0.50 - 0.65)
1-2	783 (6.1)	7 (1.8)	0.17 (0.07 - 0.41)	6558 (7.5)	384 (3.8)	0.46 (0.40 - 0.52)
2-3	769 (6.0)	21 (5.4)	0.63 (0.37 - 1.07)	7227 (8.3)	533 (5.2)	0.46 (0.41 - 0.51)
3-4	342 (2.7)	19 (4.9)	0.95 (0.53 - 1.71)	3855 (4.4)	417 (4.1)	0.55 (0.48 - 0.62)
4-5	144 (1.1)	11 (2.8)	1.18 (0.57 - 2.44)	1399 (1.6)	195 (1.9)	0.59 (0.49 - 0.71)
5-6	21 (0.2)	2 (0.5)	2.16 (0.40 - 11.8)	299 (0.3)	48 (0.5)	0.72 (0.50 - 1.02)
6 or more	155 (1.2)	2 (0.5)	0.53 (0.12 - 2.38)	712 (0.8)	63 (0.6)	0.79 (0.59 - 1.07)
Immune-suppressive medications before breakthrough (%)						
Before breakthrough Chemotherapy	42 (0.3)	7 (1.8)	4.55 (1.25 - 16.5)	654 (0.7)	303 (3.0)	2.46 (2.03 - 2.99)
Before breakthrough Cytokine-blocking	199 (1.6)	13 (3.4)	0.97 (0.38 - 2.50)	1202 (1.4)	187 (1.8)	1.62 (1.25 - 2.10)
Before breakthrough Glucocorticoids	762 (6.0)	57 (14.7)	1.64 (1.16 - 2.33)	4961 (5.7)	1764 (17.3)	2.36 (2.20 - 2.54)
Before breakthrough Leukocyte-inhibitory	227 (1.8)	29 (7.5)	2.62 (1.24 - 5.53)	1211 (1.4)	457 (4.5)	2.48 (2.08 - 2.97)
Before breakthrough Lymphocyte-depleting	59 (0.5)	10 (2.6)	2.37 (0.69 - 8.11)	347 (0.4)	169 (1.7)	1.77 (1.30 - 2.40)
Immune-suppressive medications before initial vaccination (%)						0.79 (0.57 - 1.10)
Before vaccine Chemotherapy	24 (0.2)	3 (0.8)	0.44 (0.07 - 2.82)	292 (0.3)	111 (1.1)	0.61 (0.44 - 0.84)
Before vaccine Cytokine-blocking	166 (1.3)	10 (2.6)	0.96 (0.32 - 2.90)	930 (1.1)	105 (1.0)	1.31 (1.16 - 1.48)
Before vaccine Glucocorticoids	264 (2.1)	19 (4.9)	1.34 (0.76 - 2.35)	1860 (2.1)	611 (6.0)	0.76 (0.60 - 0.95)
Before vaccine Leukocyte-inhibitory	181 (1.4)	21 (5.4)	1.13 (0.49 - 2.62)	918 (1.1)	248 (2.4)	1.24 (0.76 - 2.01)
Before vaccine Lymphocyte-depleting	24 (0.2)	4 (1.0)	0.31 (0.04 - 2.45)	129 (0.1)	66 (0.6)	1.47 (1.14 - 1.89)

© 2022 Vo AD et al. *JAMA Network Open.*

Persistent positive test (%)	27 (0.2)	5 (1.3)	3.37 (1.07 - 10.7)	332 (0.4)	98 (1.0)	2.46 (2.03 - 2.99)
BMI Class (%)						
Underweight	67 (0.5)	10 (2.6)	2.04 (0.86 - 4.82)	429 (0.5)	171 (1.7)	1.43 (1.14 - 1.79)
Normal	1685 (13.2)	67 (17.3)	1.0 (referent)	10614 (12.1)	2028 (19.8)	1.0 (referent)
Overweight	3111 (24.4)	64 (16.5)	0.49 (0.33 - 0.74)	26681 (30.5)	2851 (27.9)	0.76 (0.70 - 0.82)
Obesity I	3276 (25.7)	88 (22.7)	0.74 (0.51 - 1.08)	25281 (28.9)	2511 (24.6)	0.82 (0.76 - 0.89)
Obesity II	2147 (16.8)	69 (17.8)	0.87 (0.58 - 1.29)	13188 (15.1)	1458 (14.3)	1.01 (0.92 - 1.10)
Severe Obesity	1602 (12.6)	86 (22.2)	1.31 (0.88 - 1.95)	7908 (9.0)	1045 (10.2)	1.30 (1.17 - 1.43)
Unknown	871 (6.8)	3 (0.8)	0.27 (0.08 - 0.92)	3288 (3.8)	161 (1.6)	0.95 (0.78 - 1.16)
Comorbidities (%)						
Acquired hypothyroidism	897 (7.0)	46 (11.9)	0.76 (0.52 - 1.12)	3276 (3.7)	746 (7.3)	1.03 (0.93 - 1.14)
Acute myocardial infarction	27 (0.2)	5 (1.3)	0.92 (0.28 – 3.00)	546 (0.6)	209 (2.0)	1.14 (0.93 - 1.40)
Alzheimers disease and related disorders or senile dementia	76 (0.6)	19 (4.9)	1.57 (0.83 - 2.97)	1839 (2.1)	1116 (10.9)	1.93 (1.75 - 2.13)
Anemia	852 (6.7)	71 (18.3)	1.24 (0.85 - 1.79)	5416 (6.2)	1795 (17.6)	1.18 (1.09 - 1.27)
Anxiety disorders	4729 (37.1)	133 (34.4)	1.02 (0.77 - 1.34)	19614 (22.4)	1805 (17.7)	0.90 (0.84 - 0.96)
Asthma	785 (6.2)	40 (10.3)	1.35 (0.91 - 1.99)	2421 (2.8)	272 (2.7)	0.90 (0.78 - 1.05)
Atrial fibrillation	123 (1.0)	18 (4.7)	0.61 (0.33 - 1.16)	4176 (4.8)	1482 (14.5)	1.21 (1.12 - 1.32)
Bipolar disorder	1152 (9.0)	50 (12.9)	1.53 (1.07 - 2.19)	4502 (5.2)	593 (5.8)	1.23 (1.10 - 1.37)
Chronic kidney disease	530 (4.2)	95 (24.5)	2.14 (1.51 - 3.02)	8541 (9.8)	2666 (26.1)	1.52 (1.42 - 1.63)
Chronic obstructive pulmonary disease and bronchiectasis	343 (2.7)	71 (18.3)	2.27 (1.57 - 3.29)	5760 (6.6)	2163 (21.2)	1.72 (1.60 - 1.84)
Colorectal cancer	26 (0.2)	1 (0.3)	0.46 (0.05 - 3.89)	461 (0.5)	120 (1.2)	1.01 (0.79 - 1.29)
Depression	4428 (34.7)	151 (39.0)	1.04 (0.79 - 1.37)	16089 (18.4)	1874 (18.3)	1.10 (1.02 - 1.18)
Diabetes	1489 (11.7)	135 (34.9)	1.38 (1.03 - 1.85)	20430 (23.4)	4029 (39.4)	1.24 (1.17 - 1.31)
Epilepsy	102 (0.8)	11 (2.8)	2.10 (0.94 - 4.72)	768 (0.9)	200 (2.0)	1.48 (1.22 - 1.79)
Heart failure	130 (1.0)	36 (9.3)	2.04 (1.21 - 3.47)	3551 (4.1)	1727 (16.9)	1.70 (1.56 - 1.85)
HIV/AIDS	30 (0.2)	5 (1.3)	5.60 (1.85 - 16.9)	761 (0.9)	78 (0.8)	1.22 (0.93 - 1.60)
Hyperlipidemia	2190 (17.2)	139 (35.9)	0.96 (0.72 - 1.27)	25434 (29.1)	3748 (36.7)	0.81 (0.76 - 0.86)
Hypertension	2556 (20.0)	193 (49.9)	1.26 (0.95 - 1.66)	30634 (35.1)	5526 (54.0)	1.07 (1.01 - 1.14)
Ischemic heart disease	259 (2.0)	46 (11.9)	1.14 (0.71 - 1.82)	8461 (9.7)	2241 (21.9)	0.97 (0.90 - 1.04)
Leukemias and lymphomas	38 (0.3)	6 (1.6)	1.37 (0.44 - 4.25)	955 (1.1)	337 (3.3)	1.60 (1.36 - 1.90)
Liver disease: cirrhosis and other liver conditions except viral hepatitis	326 (2.6)	21 (5.4)	1.07 (0.62 - 1.83)	3242 (3.7)	604 (5.9)	1.30 (1.16 - 1.46)
Lung cancer	27 (0.2)	6 (1.6)	1.68 (0.57 - 4.98)	546 (0.6)	245 (2.4)	1.53 (1.27 - 1.84)
Mobility impairments	49 (0.4)	10 (2.6)	2.61 (1.08 - 6.31)	679 (0.8)	292 (2.9)	1.97 (1.66 - 2.34)
Multiple sclerosis and transverse myelitis	111 (0.9)	10 (2.6)	3.01 (1.35 - 6.73)	251 (0.3)	82 (0.8)	3.13 (2.30 - 4.26)
Peripheral vascular disease	84 (0.7)	17 (4.4)	0.93 (0.47 - 1.83)	2157 (2.5)	839 (8.2)	1.22 (1.10 - 1.36)
Pressure and chronic ulcers	30 (0.2)	12 (3.1)	2.33 (0.95 - 5.75)	842 (1.0)	463 (4.5)	1.52 (1.31 - 1.76)

© 2022 Vo AD et al. *JAMA Network Open.*

Prostate cancer	1 (0.0)	0 (0.0)	ND	3181 (3.6)	633 (6.2)	0.92 (0.83 - 1.02)
Schizophrenia and other psychotic disorders	284 (2.2)	23 (5.9)	1.61 (0.94 - 2.74)	2036 (2.3)	412 (4.0)	1.68 (1.47 - 1.92)
Tobacco use	842 (6.6)	55 (14.2)	1.28 (0.88 - 1.86)	7272 (8.3)	1153 (11.3)	1.37 (1.26 - 1.49)
Viral hepatitis	52 (0.4)	5 (1.3)	1.11 (0.34 - 3.65)	1128 (1.3)	231 (2.3)	0.98 (0.82 - 1.17)
Death from breakthrough	0 (0.0)	21 (5.4)		0 (0.0)	1534 (15.0)	

OR = odds ratio. CI = 95% confidence interval.

© 2022 Vo AD et al. *JAMA Network Open.*

eTable 16. Subcohort During Delta Period, Data and Analysis

	Overall	Non-severe	Severe	Multivariable OR (CI)	P (multi)
N	29493	24762	4731		
Sex (%)					
Male	26781 (90.8)	22206 (89.7)	4575 (96.7)	1.0 (referent	
Female	2712 (9.2)	2556 (10.3)	156 (3.3)	0.66 (0.55 - 0.8)	1.2E-05 [a]
Age ranges (%)					
Less than 40	2306 (7.8)	2276 (9.2)	30 (0.6)	0.37 (0.23 - 0.59)	2.6E-05 [a]
40-45	1201 (4.1)	1169 (4.7)	32 (0.7)	0.64 (0.40 - 1.02)	0.06
45-50	1274 (4.3)	1221 (4.9)	53 (1.1)	1.0 (referent)	
50-55	2082 (7.1)	1957 (7.9)	125 (2.6)	1.38 (0.98 - 1.95)	0.06
55-60	2654 (9.0)	2465 (10.0)	189 (4.0)	1.53 (1.11 - 2.12)	0.01 [a]
60-65	3411 (11.6)	3025 (12.2)	386 (8.2)	2.32 (1.71 - 3.16)	8.4E-08
65-70	3550 (12.0)	2986 (12.1)	564 (11.9)	3.20 (2.36 - 4.34)	<1.0E-08 [a]
70-75	6141 (20.8)	4908 (19.8)	1233 (26.1)	4.61 (3.43 - 6.21)	<1.0E-08 [a]
75-80	3656 (12.4)	2757 (11.1)	899 (19.0)	6.06 (4.47 - 8.20)	<1.0E-08 [a]
80 or greater	3218 (10.9)	1998 (8.1)	1220 (25.8)	10.9 (8.06 - 14.9)	<1.0E-08 [a]
Race (%)					
American Indian or Alaska Native	231 (0.8)	193 (0.8)	38 (0.8)	1.13 (0.74 - 1.71)	0.57
Asian	245 (0.8)	222 (0.9)	23 (0.5)	1.05 (0.63 - 1.74)	0.86
Black or African American	5322 (18.0)	4485 (18.1)	837 (17.7)	1.14 (1.03 - 1.26)	0.01 [a]
Native Hawaiian or other Pacific Islander	242 (0.8)	205 (0.8)	37 (0.8)	1.24 (0.82 - 1.85)	0.31
White	21418 (72.6)	17885 (72.2)	3533 (74.7)	1.0 (referent)	
Unknown	2035 (6.9)	1772 (7.2)	263 (5.6)	0.92 (0.78 - 1.09)	0.34
Ethnicity Hispanic/Latino (%)					
Hispanic or Latino	2322 (7.9)	1986 (8.0)	336 (7.1)	1.25 (1.08 - 1.45)	0.003 [a]
Not Hispanic or Latino	25769 (87.4)	21567 (87.1)	4202 (88.8)	1.0 (referent)	
Unknown	1402 (4.8)	1209 (4.9)	193 (4.1)	1.26 (1.03 - 1.54)	0.03
US Region (%)					
Continental	4864 (16.5)	3944 (15.9)	920 (19.4)	1.31 (1.16 - 1.46)	5.0E-06 [a]
Midwest	6765 (22.9)	5682 (22.9)	1083 (22.9)	0.96 (0.86 - 1.07)	0.45
North Atlantic	6073 (20.6)	5182 (20.9)	891 (18.8)	0.90 (0.81 - 1.01)	0.09
Pacific	5028 (17.0)	4261 (17.2)	767 (16.2)	1.05 (0.93 - 1.19)	0.39
Southeast	6368 (21.6)	5305 (21.4)	1063 (22.5)	1.0 (referent)	
Unknown	395 (1.3)	388 (1.6)	7 (0.1)	0.26 (0.12 - 0.60)	0.001 [a]
Vaccine type (%)					
Janssen	3449 (11.7)	2969 (12.0)	480 (10.1)	1.24 (1.09 - 1.41)	9.3E-04 [a]

© 2022 Vo AD et al. *JAMA Network Open.*

Moderna	11809 (40.0)	9968 (40.3)	1841 (38.9)	0.72 (0.67 - 0.78)	<1.0E-08 [a]
Pfizer	14235 (48.3)	11825 (47.8)	2410 (50.9)	1.0 (referent)	
History of infection before vaccinated (%)	598 (2.0)	487 (2.0)	111 (2.3)	0.85 (0.66 - 1.09)	0.20
Months since full vax at breakthrough (%)					
4 or less	5400 (18.3)	4697 (19.0)	703 (14.9)	1.0 (referent)	
4-5	4750 (16.1)	4062 (16.4)	688 (14.5)	0.99 (0.86 - 1.13)	0.85
5-6	5901 (20.0)	4942 (20.0)	959 (20.3)	0.93 (0.82 - 1.06)	0.31
6-7	5506 (18.7)	4546 (18.4)	960 (20.3)	1.04 (0.91 - 1.19)	0.54
7-8	4263 (14.5)	3539 (14.3)	724 (15.3)	1.08 (0.94 - 1.25)	0.26
8-9	2569 (8.7)	2092 (8.4)	477 (10.1)	1.10 (0.94 - 1.29)	0.24
9-10	1007 (3.4)	803 (3.2)	204 (4.3)	1.23 (0.99 - 1.53)	0.06
10-11	95 (0.3)	79 (0.3)	16 (0.3)	1.92 (1.00 - 3.68)	0.05
11-12	2 (0.0)	2 (0.0)	0 (0.0)	ND	ND
12 or more	0	0	0	ND	ND
Months since boosted at breakthrough (%)					
Not boosted	28142 (95.4)	23636 (95.5)	4506 (95.2)	1.0 (referent)	
1 or less	653 (2.2)	542 (2.2)	111 (2.3)	0.69 (0.54 - 0.88)	0.003 [a]
1-2	251 (0.9)	206 (0.8)	45 (1.0)	0.62 (0.42 - 0.91)	0.01 [a]
2-3	158 (0.5)	129 (0.5)	29 (0.6)	0.46 (0.27 - 0.77)	0.004 [a]
3-4	49 (0.2)	44 (0.2)	5 (0.1)	0.36 (0.12 - 1.08)	0.07
4-5	39 (0.1)	32 (0.1)	7 (0.1)	2.25 (0.95 - 5.36)	0.07
5-6	59 (0.2)	52 (0.2)	7 (0.1)	0.98 (0.41 - 2.36)	0.96
6 or more	142 (0.5)	121 (0.5)	21 (0.4)	1.22 (0.73 - 2.04)	0.45
Immune-suppressive medications before breakthrough (%)					
Before breakthrough Chemotherapy	304 (1.0)	186 (0.8)	118 (2.5)	2.40 (1.72 - 3.36)	3.1E-07 [a]
Before breakthrough Cytokine-blocking	506 (1.7)	395 (1.6)	111 (2.3)	1.61 (1.09 - 2.38)	0.02 [a]
Before breakthrough Glucocorticoids	2595 (8.8)	1812 (7.3)	783 (16.6)	1.93 (1.73 - 2.16)	<1.0E-08 [a]
Before breakthrough Leukocyte-inhibitory	568 (1.9)	356 (1.4)	212 (4.5)	2.77 (2.06 - 3.71)	<1.0E-08 [a]
Before breakthrough Lymphocyte-depleting	167 (0.6)	106 (0.4)	61 (1.3)	1.26 (0.73 - 2.18)	0.40
Immune-suppressive medications before initial vaccination (%)					
Before vaccine Chemotherapy	150 (0.5)	100 (0.4)	50 (1.1)	0.75 (0.45 - 1.26)	0.29
Before vaccine Cytokine-blocking	375 (1.3)	308 (1.2)	67 (1.4)	0.78 (0.48 - 1.25)	0.30
Before vaccine Glucocorticoids	846 (2.9)	570 (2.3)	276 (5.8)	1.37 (1.13 - 1.65)	0.001 [a]
Before vaccine Leukocyte-inhibitory	393 (1.3)	277 (1.1)	116 (2.5)	0.68 (0.47 - 0.98)	0.04
Before vaccine Lymphocyte-depleting	66 (0.2)	40 (0.2)	26 (0.5)	1.05 (0.45 - 2.45)	0.91
Persistent positive test (%)	231 (0.8)	182 (0.7)	49 (1.0)	1.12 (0.79 - 1.59)	0.52
BMI Class (%)					

© 2022 Vo AD et al. *JAMA Network Open.*

Underweight	193 (0.7)	119 (0.5)	74 (1.6)	1.60 (1.10 - 2.32)	0.01 [a]
Normal	3858 (13.1)	3044 (12.3)	814 (17.2)	1.0 (referent)	
Overweight	8545 (29.0)	7261 (29.3)	1284 (27.1)	0.82 (0.73 - 0.92)	9.9E-04 [a]
Obesity I	8366 (28.4)	7120 (28.8)	1246 (26.3)	0.94 (0.83 - 1.06)	0.30
Obesity II	4672 (15.8)	3984 (16.1)	688 (14.5)	1.07 (0.93 - 1.23)	0.33
Severe Obesity	3037 (10.3)	2487 (10.0)	550 (11.6)	1.57 (1.36 - 1.82)	<1.0E-08 [a]
Unknown	822 (2.8)	747 (3.0)	75 (1.6)	1.14 (0.84 - 1.53)	0.40
Comorbidities (%)					
Acquired hypothyroidism	1499 (5.1)	1143 (4.6)	356 (7.5)	1.10 (0.95 - 1.28)	0.22
Acute myocardial infarction	231 (0.8)	150 (0.6)	81 (1.7)	1.15 (0.83 - 1.61)	0.40
Alzheimers disease and related disorders or senile dementia	937 (3.2)	485 (2.0)	452 (9.6)	1.96 (1.67 - 2.30)	<1.0E-08 [a]
Anemia	2352 (8.0)	1612 (6.5)	740 (15.6)	1.15 (1.02 - 1.30)	0.03
Anxiety disorders	6171 (20.9)	5354 (21.6)	817 (17.3)	0.90 (0.81 - 1.01)	0.06
Asthma	902 (3.1)	746 (3.0)	156 (3.3)	1.09 (0.89 - 1.35)	0.40
Atrial fibrillation	1946 (6.6)	1305 (5.3)	641 (13.5)	1.16 (1.02 - 1.32)	0.02 [a]
Bipolar disorder	1549 (5.3)	1280 (5.2)	269 (5.7)	1.26 (1.06 - 1.50)	0.008 [a]
Chronic kidney disease	3457 (11.7)	2329 (9.4)	1128 (23.8)	1.44 (1.30 - 1.61)	<1.0E-08 [a]
Chronic obstructive pulmonary disease and bronchiectasis	2668 (9.0)	1741 (7.0)	927 (19.6)	1.64 (1.47 - 1.83)	<1.0E-08 [a]
Colorectal cancer	185 (0.6)	136 (0.5)	49 (1.0)	0.98 (0.66 - 1.45)	0.92
Depression	5496 (18.6)	4634 (18.7)	862 (18.2)	1.15 (1.03 - 1.28)	0.01 [a]
Diabetes	7862 (26.7)	5971 (24.1)	1891 (40.0)	1.31 (1.20 - 1.43)	<1.0E-08 [a]
Epilepsy	287 (1.0)	195 (0.8)	92 (1.9)	1.81 (1.33 - 2.45)	1.6E-04 [a]
Heart failure	1707 (5.8)	1002 (4.0)	705 (14.9)	1.54 (1.35 - 1.77)	<1.0E-08 [a]
HIV/AIDS	253 (0.9)	212 (0.9)	41 (0.9)	1.43 (0.97 - 2.12)	0.07
Hyperlipidemia	8677 (29.4)	6994 (28.2)	1683 (35.6)	0.83 (0.76 - 0.91)	6.7E-05 [a]
Hypertension	11170 (37.9)	8678 (35.0)	2492 (52.7)	1.07 (0.98 - 1.17)	0.15
Ischemic heart disease	3494 (11.8)	2507 (10.1)	987 (20.9)	0.99 (0.89 - 1.11)	0.92
Leukemias and lymphomas	394 (1.3)	272 (1.1)	122 (2.6)	1.23 (0.93 - 1.64)	0.15
Liver disease: cirrhosis and other liver conditions except viral hepatitis	1173 (4.0)	916 (3.7)	257 (5.4)	1.24 (1.04 - 1.47)	0.02 [a]
Lung cancer	250 (0.8)	149 (0.6)	101 (2.1)	1.69 (1.25 - 2.29)	6.8E-04 [a]
Mobility impairments	319 (1.1)	186 (0.8)	133 (2.8)	2.06 (1.56 - 2.73)	3.7E-07 [a]
Multiple sclerosis and transverse myelitis	111 (0.4)	78 (0.3)	33 (0.7)	3.19 (1.96 - 5.20)	3.3E-06 [a]
Peripheral vascular disease	985 (3.3)	638 (2.6)	347 (7.3)	1.13 (0.96 - 1.34)	0.15
Pressure and chronic ulcers	428 (1.5)	240 (1.0)	188 (4.0)	1.38 (1.07 - 1.77)	0.01 [a]
Prostate cancer	1127 (3.8)	874 (3.5)	253 (5.3)	0.85 (0.72 – 1.00)	0.05
Schizophrenia and other psychotic disorders	677 (2.3)	514 (2.1)	163 (3.4)	1.64 (1.32 - 2.03)	8.5E-06 [a]

© 2022 Vo AD et al. *JAMA Network Open.*

Tobacco use	2399 (8.1)	1930 (7.8)	469 (9.9)	1.22 (1.07 - 1.40)	0.004 [a]
Viral hepatitis	374 (1.3)	279 (1.1)	95 (2.0)	1.07 (0.80 - 1.42)	0.65
Death from breakthrough infection	736 (2.5)	0 (0.0)	736 (15.6)		

[a] Meets criteria for statistical significance after adjustment for multiple comparisons, P<0.024 in this analysis. OR = odds ratio. CI = 95% confidence interval.

© 2022 Vo AD et al. *JAMA Network Open.*

eTable 17. Subcohort During Omicron Period, Data and Analysis

	Overall	Non-severe	Severe	Multivariable OR (CI)	P (multi)
N	78379	73135	5244		
Sex (%)					
Male	68128 (86.9)	63099 (86.3)	5029 (95.9)	1.0 (referent)	
Female	11251 (13.1)	10036 (13.7)	215 (4.1)	0.73 (0.63 - 0.86)	7.6E-05 [a]
Age ranges (%)					
Less than 40	10702 (13.7)	10626 (14.5)	76 (1.4)	0.70 (0.49 - 1.00)	0.05
40-45	4968 (6.3)	4914 (6.7)	54 (1.0)	1.04 (0.71 - 1.53)	0.85
45-50	4963 (6.3)	4909 (6.7)	54 (1.0)	1.0 (referent)	
50-55	7489 (9.6)	7344 (10.0)	145 (2.8)	1.69 (1.23 - 2.33)	0.001 [a]
55-60	8088 (10.3)	7827 (10.7)	261 (5.0)	2.59 (1.92 - 3.50)	<1.0E-08 [a]
60-65	9247 (11.8)	8791 (12.0)	456 (8.7)	3.61 (2.70 - 4.83)	<1.0E-08 [a]
65-70	8441 (10.8)	7747 (10.6)	694 (13.2)	5.43 (4.07 - 7.25)	<1.0E-08 [a]
70-75	11485 (14.7)	10256 (14.0)	1229 (23.4)	7.26 (5.46 - 9.65)	<1.0E-08 [a]
75-80	7449 (9.5)	6457 (8.8)	992 (18.9)	9.20 (6.91 - 12.3)	<1.0E-08 [a]
80 or greater	5547 (7.1)	4264 (5.8)	1283 (24.5)	16.6 (12.4 - 22.2)	<1.0E-08 [a]
Race (%)					
American Indian or Alaska Native	642 (0.8)	596 (0.8)	46 (0.9)	1.42 (1.00 - 2.01)	0.05
Asian	1321 (1.7)	1289 (1.8)	32 (0.6)	0.77 (0.52 - 1.15)	0.21
Black or African American	21032 (26.8)	19824 (27.1)	1208 (23.0)	0.88 (0.81 - 0.95)	0.002 [a]
Native Hawaiian or other Pacific Islander	868 (1.1)	835 (1.1)	33 (0.6)	0.59 (0.38 - 0.89)	0.01 [a]
White	48203 (61.5)	44563 (60.9)	3640 (69.4)	1.0 (referent)	
Unknown	6313 (8.1)	6028 (8.2)	285 (5.4)	0.87 (0.74 - 1.01)	0.07
Ethnicity Hispanic/Latino (%)					
Hispanic or Latino	8679 (11.1)	8305 (11.4)	374 (7.1)	0.94 (0.83 - 1.07)	0.32
Not Hispanic or Latino	65094 (83.1)	60413 (82.6)	4681 (89.3)	1.0 (referent)	
Unknown	4606 (5.9)	4417 (6.0)	189 (3.6)	0.99 (0.82 - 1.19)	0.91
US Region (%)					
Continental	12828 (16.4)	11945 (16.3)	883 (16.8)	1.04 (0.94 - 1.16)	0.41
Midwest	14506 (18.5)	13418 (18.3)	1088 (20.7)	0.95 (0.86 - 1.05)	0.34
North Atlantic	17017 (21.7)	15850 (21.7)	1167 (22.3)	0.95 (0.86 - 1.05)	0.30
Pacific	14756 (18.8)	13915 (19.0)	841 (16.0)	0.87 (0.78 - 0.97)	0.01 [a]
Southeast	17381 (22.2)	16131 (22.1)	1250 (23.8)	1.0 (referent)	
Unknown	1891 (2.4)	1876 (2.6)	15 (0.3)	0.33 (0.18 - 0.59)	1.7E-04 [a]
Vaccine type (%)					
Janssen	7406 (9.4)	6884 (9.4)	522 (10.0)	1.27 (1.13 - 1.44)	7.6E-05 [a]

© 2022 Vo AD et al. *JAMA Network Open.*

Moderna	32604 (41.6)	30449 (41.6)	2155 (41.1)	0.84 (0.78 - 0.90)	1.2E-06 [a]
Pfizer	38369 (49.0)	35802 (49.0)	2567 (49.0)	1.0 (referent)	
History of infection before vaccinated (%)	3152 (4.0)	3015 (4.1)	137 (2.6)	0.56 (0.46 - 0.69)	2.6E-08 [a]
Months since full vax at breakthrough (%)					
4 or less	4272 (5.5)	4079 (5.6)	193 (3.7)	1.0 (referent)	
4-5	2408 (3.1)	2252 (3.1)	156 (3.0)	1.37 (1.06 - 1.77)	0.02 [a]
5-6	2242 (2.9)	2094 (2.9)	148 (2.8)	1.30 (1.00 - 1.69)	0.05
6-7	3396 (4.3)	3217 (4.4)	179 (3.4)	1.23 (0.96 - 1.57)	0.11
7-8	7476 (9.5)	7126 (9.7)	350 (6.7)	1.39 (1.13 - 1.72)	0.002 [a]
8-9	16549 (21.1)	15781 (21.6)	768 (14.6)	1.32 (1.09 - 1.60)	0.005 [a]
9-10	18990 (24.2)	17767 (24.3)	1223 (23.3)	1.28 (1.06 - 1.55)	0.009 [a]
10-11	15636 (19.9)	14173 (19.4)	1463 (27.9)	1.49 (1.23 - 1.81)	3.8E-05 [a]
11-12	6517 (8.3)	5851 (8.0)	666 (12.7)	1.73 (1.40 - 2.12)	2.0E-07 [a]
12 or more	893 (1.1)	795 (1.1)	98 (1.9)	1.88 (1.38 - 2.57)	5.7E-05 [a]
Months since boosted at breakthrough (%)					
Not boosted	50047 (63.9)	46630 (63.8)	3417 (65.2)	1.0 (referent)	
1 or less	5085 (6.5)	4843 (6.6)	242 (4.6)	0.54 (0.46 - 0.62)	<1.0E-08 [a]
1-2	7476 (9.5)	7130 (9.7)	346 (6.6)	0.43 (0.37 - 0.48)	<1.0E-08 [a]
2-3	8384 (10.7)	7860 (10.7)	524 (10.0)	0.46 (0.41 - 0.52)	<1.0E-08 [a]
3-4	4580 (5.8)	4150 (5.7)	430 (8.2)	0.55 (0.48 - 0.62)	<1.0E-08 [a]
4-5	1707 (2.2)	1509 (2.1)	198 (3.8)	0.57 (0.47 - 0.69)	<1.0E-08 [a]
5-6	310 (0.4)	267 (0.4)	43 (0.8)	0.66 (0.45 - 0.98)	0.04 [a]
6 or more	790 (1.0)	746 (1.0)	44 (0.8)	0.63 (0.44 - 0.91)	0.01 [a]
Immune-suppressive medications before breakthrough (%)					
Before breakthrough Chemotherapy	649 (0.8)	484 (0.7)	165 (3.1)	2.49 (1.95 - 3.18)	<1.0E-08 [a]
Before breakthrough Cytokine-blocking	1058 (1.3)	978 (1.3)	80 (1.5)	1.46 (1.04 - 2.05)	0.03 [a]
Before breakthrough Glucocorticoids	4683 (6.0)	3765 (5.1)	918 (17.5)	2.67 (2.42 - 2.95)	<1.0E-08 [a]
Before breakthrough Leukocyte-inhibitory	1284 (1.6)	1042 (1.4)	242 (4.6)	2.22 (1.77 - 2.79)	<1.0E-08 [a]
Before breakthrough Lymphocyte-depleting	401 (0.5)	293 (0.4)	108 (2.1)	2.19 (1.52 - 3.14)	<1.0E-08 [a]
Immune-suppressive medications before initial vaccination (%)					
Before vaccine Chemotherapy	257 (0.3)	202 (0.3)	55 (1.0)	0.86 (0.56 - 1.31)	0.48
Before vaccine Cytokine-blocking	813 (1.0)	769 (1.1)	44 (0.8)	0.52 (0.34 - 0.81)	0.004 [a]
Before vaccine Glucocorticoids	1788 (2.3)	1484 (2.0)	304 (5.8)	1.28 (1.09 - 1.51)	0.003 [a]
Before vaccine Leukocyte-inhibitory	933 (1.2)	792 (1.1)	141 (2.7)	0.96 (0.72 - 1.27)	0.76
Before vaccine Lymphocyte-depleting	149 (0.2)	111 (0.2)	38 (0.7)	1.02 (0.57 - 1.82)	0.96
Persistent positive test (%)	198 (0.3)	151 (0.2)	47 (0.9)	2.36 (1.61 - 3.48)	1.3E-05 [a]
BMI Class (%)					

© 2022 Vo AD et al. *JAMA Network Open.*

Underweight	452 (0.6)	357 (0.5)	95 (1.8)	1.48 (1.11 - 1.97)	0.007 [a]
Normal	10055 (12.8)	8927 (12.2)	1128 (21.5)	1.0 (referent)	
Overweight	23253 (29.7)	21802 (29.8)	1451 (27.7)	0.71 (0.64 - 0.78)	<1.0E-08 [a]
Obesity I	22054 (28.1)	20848 (28.5)	1206 (23.0)	0.75 (0.68 - 0.83)	5.1E-08 [a]
Obesity II	11774 (15.0)	11010 (15.1)	764 (14.6)	1.00 (0.89 - 1.13)	0.99
Severe Obesity	7335 (9.4)	6819 (9.3)	516 (9.8)	1.17 (1.02 - 1.34)	0.02 [a]
Unknown	3456 (4.4)	3372 (4.6)	84 (1.6)	0.79 (0.61 - 1.02)	0.08
Comorbidities (%)					
Acquired hypothyroidism	3296 (4.2)	2917 (4.0)	379 (7.2)	0.94 (0.82 - 1.08)	0.38
Acute myocardial infarction	506 (0.6)	394 (0.5)	112 (2.1)	1.05 (0.80 - 1.39)	0.71
Alzheimers disease and related disorders or senile dementia	1885 (2.4)	1293 (1.8)	592 (11.3)	2.00 (1.75 - 2.27)	<1.0E-08 [a]
Anemia	5305 (6.8)	4359 (6.0)	946 (18.0)	1.20 (1.08 - 1.33)	7.2E-04 [a]
Anxiety disorders	19505 (24.9)	18500 (25.3)	1005 (19.2)	0.90 (0.82 - 0.98)	0.02 [a]
Asthma	2525 (3.2)	2381 (3.3)	144 (2.7)	0.92 (0.76 - 1.12)	0.42
Atrial fibrillation	3516 (4.5)	2779 (3.8)	737 (14.1)	1.22 (1.09 - 1.37)	5.5E-04 [a]
Bipolar disorder	4603 (5.9)	4255 (5.8)	348 (6.6)	1.25 (1.09 - 1.44)	0.002 [a]
Chronic kidney disease	7772 (9.9)	6357 (8.7)	1415 (27.0)	1.66 (1.51 - 1.82)	<1.0E-08 [a]
Chronic obstructive pulmonary disease and bronchiectasis	5299 (6.8)	4145 (5.7)	1154 (22.0)	1.75 (1.59 - 1.92)	<1.0E-08 [a]
Colorectal cancer	389 (0.5)	329 (0.4)	60 (1.1)	0.95 (0.68 - 1.34)	0.79
Depression	16534 (21.1)	15478 (21.2)	1056 (20.1)	1.08 (0.98 - 1.19)	0.12
Diabetes	17226 (22.0)	15250 (20.9)	1976 (37.7)	1.18 (1.09 - 1.28)	2.7E-05 [a]
Epilepsy	744 (0.9)	639 (0.9)	105 (2.0)	1.38 (1.07 - 1.77)	0.01 [a]
Heart failure	3379 (4.3)	2477 (3.4)	902 (17.2)	1.84 (1.65 - 2.07)	<1.0E-08 [a]
HIV/AIDS	600 (0.8)	561 (0.8)	39 (0.7)	1.23 (0.84 - 1.80)	0.29
Hyperlipidemia	21819 (27.8)	19890 (27.2)	1929 (36.8)	0.79 (0.73 - 0.85)	<1.0E-08 [a]
Hypertension	26365 (33.6)	23518 (32.2)	2847 (54.3)	1.10 (1.01 - 1.19)	0.02 [a]
Ischemic heart disease	6967 (8.9)	5843 (8.0)	1124 (21.4)	0.97 (0.87 - 1.07)	0.49
Leukemias and lymphomas	853 (1.1)	675 (0.9)	178 (3.4)	1.81 (1.45 - 2.25)	1.5E-07 [a]
Liver disease: cirrhosis and other liver conditions except viral hepatitis	2853 (3.6)	2538 (3.5)	315 (6.0)	1.31 (1.13 - 1.53)	4.0E-04 [a]
Lung cancer	522 (0.7)	389 (0.5)	133 (2.5)	1.60 (1.25 - 2.04)	1.5E-04 [a]
Mobility impairments	648 (0.8)	499 (0.7)	149 (2.8)	2.08 (1.66 - 2.61)	<1.0E-08 [a]
Multiple sclerosis and transverse myelitis	329 (0.4)	272 (0.4)	57 (1.1)	3.36 (2.34 - 4.83)	<1.0E-08 [a]
Peripheral vascular disease	1927 (2.5)	1492 (2.0)	435 (8.3)	1.25 (1.09 - 1.44)	0.002 [a]
Pressure and chronic ulcers	798 (1.0)	568 (0.8)	230 (4.4)	1.59 (1.30 - 1.93)	5.1E-06
Prostate cancer	2512 (3.2)	2186 (3.0)	326 (6.2)	0.95 (0.82 - 1.09)	0.45
Schizophrenia and other psychotic disorders	1989 (2.5)	1736 (2.4)	253 (4.8)	1.79 (1.52 - 2.11)	<1.0E-08 [a]

© 2022 Vo AD et al. *JAMA Network Open.*

Tobacco use	6718 (8.6)	6023 (8.2)	695 (13.3)	1.49 (1.34 - 1.66)	<1.0E-08 [a]
Viral hepatitis	980 (1.3)	858 (1.2)	122 (2.3)	0.89 (0.70 - 1.13)	0.34
Death from breakthrough infection	728 (0.9)	0 (0.0)	728 (13.9)		

[a] Meets criteria for statistical significance after adjustment for multiple comparisons, P<0.044 in this analysis. OR = odds ratio. CI = 95% confidence interval.

© 2022 Vo AD et al. *JAMA Network Open.*

eTable 18. Subcohorts During Delta and Omicron, Comparison of Analyses

	Non-severe	Severe	Multivariable OR (CI)	Non-severe	Severe	Multivariable OR (CI)
N	24762	4731		73135	5244	
Sex (%)						
Male	22206 (89.7)	4575 (96.7)	1.0 (referent	63099 (86.3)	5029 (95.9)	1.0 (referent)
Female	2556 (10.3)	156 (3.3)	0.66 (0.55 - 0.8)	10036 (13.7)	215 (4.1)	0.73 (0.63 - 0.86)
Age ranges (%)						
Less than 40	2276 (9.2)	30 (0.6)	0.37 (0.23 - 0.59)	10626 (14.5)	76 (1.4)	0.70 (0.49 - 1.00)
40-45	1169 (4.7)	32 (0.7)	0.64 (0.40 - 1.02)	4914 (6.7)	54 (1.0)	1.04 (0.71 - 1.53)
45-50	1221 (4.9)	53 (1.1)	1.0 (referent)	4909 (6.7)	54 (1.0)	1.0 (referent)
50-55	1957 (7.9)	125 (2.6)	1.38 (0.98 - 1.95)	7344 (10.0)	145 (2.8)	1.69 (1.23 - 2.33)
55-60	2465 (10.0)	189 (4.0)	1.53 (1.11 - 2.12)	7827 (10.7)	261 (5.0)	2.59 (1.92 - 3.50)
60-65	3025 (12.2)	386 (8.2)	2.32 (1.71 - 3.16)	8791 (12.0)	456 (8.7)	3.61 (2.70 - 4.83)
65-70	2986 (12.1)	564 (11.9)	3.20 (2.36 - 4.34)	7747 (10.6)	694 (13.2)	5.43 (4.07 - 7.25)
70-75	4908 (19.8)	1233 (26.1)	4.61 (3.43 - 6.21)	10256 (14.0)	1229 (23.4)	7.26 (5.46 - 9.65)
75-80	2757 (11.1)	899 (19.0)	6.06 (4.47 - 8.20)	6457 (8.8)	992 (18.9)	9.20 (6.91 - 12.3)
80 or greater	1998 (8.1)	1220 (25.8)	10.9 (8.06 - 14.9)	4264 (5.8)	1283 (24.5)	16.6 (12.4 - 22.2)
Race (%)						
American Indian or Alaska Native	193 (0.8)	38 (0.8)	1.13 (0.74 - 1.71)	596 (0.8)	46 (0.9)	1.42 (1.00 - 2.01)
Asian	222 (0.9)	23 (0.5)	1.05 (0.63 - 1.74)	1289 (1.8)	32 (0.6)	0.77 (0.52 - 1.15)
Black or African American	4485 (18.1)	837 (17.7)	1.14 (1.03 - 1.26)	19824 (27.1)	1208 (23.0)	0.88 (0.81 - 0.95)
Native Hawaiian or other Pacific Islander	205 (0.8)	37 (0.8)	1.24 (0.82 - 1.85)	835 (1.1)	33 (0.6)	0.59 (0.38 - 0.89)
White	17885 (72.2)	3533 (74.7)	1.0 (referent)	44563 (60.9)	3640 (69.4)	1.0 (referent)
Unknown	1772 (7.2)	263 (5.6)	0.92 (0.78 - 1.09)	6028 (8.2)	285 (5.4)	0.87 (0.74 - 1.01)
Ethnicity Hispanic/Latino (%)						
Hispanic or Latino	1986 (8.0)	336 (7.1)	1.25 (1.08 - 1.45)	8305 (11.4)	374 (7.1)	0.94 (0.83 - 1.07)
Not Hispanic or Latino	21567 (87.1)	4202 (88.8)	1.0 (referent)	60413 (82.6)	4681 (89.3)	1.0 (referent)
Unknown	1209 (4.9)	193 (4.1)	1.26 (1.03 - 1.54)	4417 (6.0)	189 (3.6)	0.99 (0.82 - 1.19)
US Region (%)						
Continental	3944 (15.9)	920 (19.4)	1.31 (1.16 - 1.46)	11945 (16.3)	883 (16.8)	1.04 (0.94 - 1.16)
Midwest	5682 (22.9)	1083 (22.9)	0.96 (0.86 - 1.07)	13418 (18.3)	1088 (20.7)	0.95 (0.86 - 1.05)
North Atlantic	5182 (20.9)	891 (18.8)	0.90 (0.81 - 1.01)	15850 (21.7)	1167 (22.3)	0.95 (0.86 - 1.05)
Pacific	4261 (17.2)	767 (16.2)	1.05 (0.93 - 1.19)	13915 (19.0)	841 (16.0)	0.87 (0.78 - 0.97)
Southeast	5305 (21.4)	1063 (22.5)	1.0 (referent)	16131 (22.1)	1250 (23.8)	1.0 (referent)
Unknown	388 (1.6)	7 (0.1)	0.26 (0.12 - 0.60)	1876 (2.6)	15 (0.3)	0.33 (0.18 - 0.59)
Vaccine type (%)						
Janssen	2969 (12.0)	480 (10.1)	1.24 (1.09 - 1.41)	6884 (9.4)	522 (10.0)	1.27 (1.13 - 1.44)

© 2022 Vo AD et al. *JAMA Network Open.*

Moderna	9968 (40.3)	1841 (38.9)	0.72 (0.67 - 0.78)	30449 (41.6)	2155 (41.1)	0.84 (0.78 - 0.90)
Pfizer	11825 (47.8)	2410 (50.9)	1.0 (referent)	35802 (49.0)	2567 (49.0)	1.0 (referent)
History of infection before vaccinated (%)	487 (2.0)	111 (2.3)	0.85 (0.66 - 1.09)	3015 (4.1)	137 (2.6)	0.56 (0.46 - 0.69)
Months since full vax at breakthrough (%)						
4 or less	4697 (19.0)	703 (14.9)	1.0 (referent)	4079 (5.6)	193 (3.7)	1.0 (referent)
4-5	4062 (16.4)	688 (14.5)	0.99 (0.86 - 1.13)	2252 (3.1)	156 (3.0)	1.37 (1.06 - 1.77)
5-6	4942 (20.0)	959 (20.3)	0.93 (0.82 - 1.06)	2094 (2.9)	148 (2.8)	1.30 (1.00 - 1.69)
6-7	4546 (18.4)	960 (20.3)	1.04 (0.91 - 1.19)	3217 (4.4)	179 (3.4)	1.23 (0.96 - 1.57)
7-8	3539 (14.3)	724 (15.3)	1.08 (0.94 - 1.25)	7126 (9.7)	350 (6.7)	1.39 (1.13 - 1.72)
8-9	2092 (8.4)	477 (10.1)	1.10 (0.94 - 1.29)	15781 (21.6)	768 (14.6)	1.32 (1.09 - 1.60)
9-10	803 (3.2)	204 (4.3)	1.23 (0.99 - 1.53)	17767 (24.3)	1223 (23.3)	1.28 (1.06 - 1.55)
10-11	79 (0.3)	16 (0.3)	1.92 (1.00 - 3.68)	14173 (19.4)	1463 (27.9)	1.49 (1.23 - 1.81)
11-12	2 (0.0)	0 (0.0)	ND	5851 (8.0)	666 (12.7)	1.73 (1.40 - 2.12)
12 or more	0	0	ND	795 (1.1)	98 (1.9)	1.88 (1.38 - 2.57)
Months since boosted at breakthrough (%)						
Not boosted	23636 (95.5)	4506 (95.2)	1.0 (referent)	46630 (63.8)	3417 (65.2)	1.0 (referent)
1 or less	542 (2.2)	111 (2.3)	0.69 (0.54 - 0.88)	4843 (6.6)	242 (4.6)	0.54 (0.46 - 0.62)
1-2	206 (0.8)	45 (1.0)	0.62 (0.42 - 0.91)	7130 (9.7)	346 (6.6)	0.43 (0.37 - 0.48)
2-3	129 (0.5)	29 (0.6)	0.46 (0.27 - 0.77)	7860 (10.7)	524 (10.0)	0.46 (0.41 - 0.52)
3-4	44 (0.2)	5 (0.1)	0.36 (0.12 - 1.08)	4150 (5.7)	430 (8.2)	0.55 (0.48 - 0.62)
4-5	32 (0.1)	7 (0.1)	2.25 (0.95 - 5.36)	1509 (2.1)	198 (3.8)	0.57 (0.47 - 0.69)
5-6	52 (0.2)	7 (0.1)	0.98 (0.41 - 2.36)	267 (0.4)	43 (0.8)	0.66 (0.45 - 0.98)
6 or more	121 (0.5)	21 (0.4)	1.22 (0.73 - 2.04)	746 (1.0)	44 (0.8)	0.63 (0.44 - 0.91)
Immune-suppressive medications before breakthrough (%)						
Before breakthrough Chemotherapy	186 (0.8)	118 (2.5)	2.40 (1.72 - 3.36)	484 (0.7)	165 (3.1)	2.49 (1.95 - 3.18)
Before breakthrough Cytokine-blocking	395 (1.6)	111 (2.3)	1.61 (1.09 - 2.38)	978 (1.3)	80 (1.5)	1.46 (1.04 - 2.05)
Before breakthrough Glucocorticoids	1812 (7.3)	783 (16.6)	1.93 (1.73 - 2.16)	3765 (5.1)	918 (17.5)	2.67 (2.42 - 2.95)
Before breakthrough Leukocyte-inhibitory	356 (1.4)	212 (4.5)	2.77 (2.06 - 3.71)	1042 (1.4)	242 (4.6)	2.22 (1.77 - 2.79)
Before breakthrough Lymphocyte-depleting	106 (0.4)	61 (1.3)	1.26 (0.73 - 2.18)	293 (0.4)	108 (2.1)	2.19 (1.52 - 3.14)
Immune-suppressive medications before initial vaccination (%)						
Before vaccine Chemotherapy	100 (0.4)	50 (1.1)	0.75 (0.45 - 1.26)	202 (0.3)	55 (1.0)	0.86 (0.56 - 1.31)
Before vaccine Cytokine-blocking	308 (1.2)	67 (1.4)	0.78 (0.48 - 1.25)	769 (1.1)	44 (0.8)	0.52 (0.34 - 0.81)
Before vaccine Glucocorticoids	570 (2.3)	276 (5.8)	1.37 (1.13 - 1.65)	1484 (2.0)	304 (5.8)	1.28 (1.09 - 1.51)
Before vaccine Leukocyte-inhibitory	277 (1.1)	116 (2.5)	0.68 (0.47 - 0.98)	792 (1.1)	141 (2.7)	0.96 (0.72 - 1.27)
Before vaccine Lymphocyte-depleting	40 (0.2)	26 (0.5)	1.05 (0.45 - 2.45)	111 (0.2)	38 (0.7)	1.02 (0.57 - 1.82)
Persistent positive test (%)	182 (0.7)	49 (1.0)	1.12 (0.79 - 1.59)	151 (0.2)	47 (0.9)	2.36 (1.61 - 3.48)
BMI Class (%)						

© 2022 Vo AD et al. *JAMA Network Open.*

Underweight	119 (0.5)	74 (1.6)	1.60 (1.10 - 2.32)	357 (0.5)	95 (1.8)	1.48 (1.11 - 1.97)
Normal	3044 (12.3)	814 (17.2)	1.0 (referent)	8927 (12.2)	1128 (21.5)	1.0 (referent)
Overweight	7261 (29.3)	1284 (27.1)	0.82 (0.73 - 0.92)	21802 (29.8)	1451 (27.7)	0.71 (0.64 - 0.78)
Obesity I	7120 (28.8)	1246 (26.3)	0.94 (0.83 - 1.06)	20848 (28.5)	1206 (23.0)	0.75 (0.68 - 0.83)
Obesity II	3984 (16.1)	688 (14.5)	1.07 (0.93 - 1.23)	11010 (15.1)	764 (14.6)	1.00 (0.89 - 1.13)
Severe Obesity	2487 (10.0)	550 (11.6)	1.57 (1.36 - 1.82)	6819 (9.3)	516 (9.8)	1.17 (1.02 - 1.34)
Unknown	747 (3.0)	75 (1.6)	1.14 (0.84 - 1.53)	3372 (4.6)	84 (1.6)	0.79 (0.61 - 1.02)
Comorbidities (%)						
Acquired hypothyroidism	1143 (4.6)	356 (7.5)	1.10 (0.95 - 1.28)	2917 (4.0)	379 (7.2)	0.94 (0.82 - 1.08)
Acute myocardial infarction	150 (0.6)	81 (1.7)	1.15 (0.83 - 1.61)	394 (0.5)	112 (2.1)	1.05 (0.80 - 1.39)
Alzheimers disease and related disorders or senile dementia	485 (2.0)	452 (9.6)	1.96 (1.67 - 2.30)	1293 (1.8)	592 (11.3)	2.00 (1.75 - 2.27)
Anemia	1612 (6.5)	740 (15.6)	1.15 (1.02 - 1.30)	4359 (6.0)	946 (18.0)	1.20 (1.08 - 1.33)
Anxiety disorders	5354 (21.6)	817 (17.3)	0.90 (0.81 - 1.01)	18500 (25.3)	1005 (19.2)	0.90 (0.82 - 0.98)
Asthma	746 (3.0)	156 (3.3)	1.09 (0.89 - 1.35)	2381 (3.3)	144 (2.7)	0.92 (0.76 - 1.12)
Atrial fibrillation	1305 (5.3)	641 (13.5)	1.16 (1.02 - 1.32)	2779 (3.8)	737 (14.1)	1.22 (1.09 - 1.37)
Bipolar disorder	1280 (5.2)	269 (5.7)	1.26 (1.06 - 1.50)	4255 (5.8)	348 (6.6)	1.25 (1.09 - 1.44)
Chronic kidney disease	2329 (9.4)	1128 (23.8)	1.44 (1.30 - 1.61)	6357 (8.7)	1415 (27.0)	1.66 (1.51 - 1.82)
Chronic obstructive pulmonary disease and bronchiectasis	1741 (7.0)	927 (19.6)	1.64 (1.47 - 1.83)	4145 (5.7)	1154 (22.0)	1.75 (1.59 - 1.92)
Colorectal cancer	136 (0.5)	49 (1.0)	0.98 (0.66 - 1.45)	329 (0.4)	60 (1.1)	0.95 (0.68 - 1.34)
Depression	4634 (18.7)	862 (18.2)	1.15 (1.03 - 1.28)	15478 (21.2)	1056 (20.1)	1.08 (0.98 - 1.19)
Diabetes	5971 (24.1)	1891 (40.0)	1.31 (1.20 - 1.43)	15250 (20.9)	1976 (37.7)	1.18 (1.09 - 1.28)
Epilepsy	195 (0.8)	92 (1.9)	1.81 (1.33 - 2.45)	639 (0.9)	105 (2.0)	1.38 (1.07 - 1.77)
Heart failure	1002 (4.0)	705 (14.9)	1.54 (1.35 - 1.77)	2477 (3.4)	902 (17.2)	1.84 (1.65 - 2.07)
HIV/AIDS	212 (0.9)	41 (0.9)	1.43 (0.97 - 2.12)	561 (0.8)	39 (0.7)	1.23 (0.84 - 1.80)
Hyperlipidemia	6994 (28.2)	1683 (35.6)	0.83 (0.76 - 0.91)	19890 (27.2)	1929 (36.8)	0.79 (0.73 - 0.85)
Hypertension	8678 (35.0)	2492 (52.7)	1.07 (0.98 - 1.17)	23518 (32.2)	2847 (54.3)	1.10 (1.01 - 1.19)
Ischemic heart disease	2507 (10.1)	987 (20.9)	0.99 (0.89 - 1.11)	5843 (8.0)	1124 (21.4)	0.97 (0.87 - 1.07)
Leukemias and lymphomas	272 (1.1)	122 (2.6)	1.23 (0.93 - 1.64)	675 (0.9)	178 (3.4)	1.81 (1.45 - 2.25)
Liver disease: cirrhosis and other liver conditions except viral hepatitis	916 (3.7)	257 (5.4)	1.24 (1.04 - 1.47)	2538 (3.5)	315 (6.0)	1.31 (1.13 - 1.53)
Lung cancer	149 (0.6)	101 (2.1)	1.69 (1.25 - 2.29)	389 (0.5)	133 (2.5)	1.60 (1.25 - 2.04)
Mobility impairments	186 (0.8)	133 (2.8)	2.06 (1.56 - 2.73)	499 (0.7)	149 (2.8)	2.08 (1.66 - 2.61)
Multiple sclerosis and transverse myelitis	78 (0.3)	33 (0.7)	3.19 (1.96 - 5.20)	272 (0.4)	57 (1.1)	3.36 (2.34 - 4.83)
Peripheral vascular disease	638 (2.6)	347 (7.3)	1.13 (0.96 - 1.34)	1492 (2.0)	435 (8.3)	1.25 (1.09 - 1.44)
Pressure and chronic ulcers	240 (1.0)	188 (4.0)	1.38 (1.07 - 1.77)	568 (0.8)	230 (4.4)	1.59 (1.30 - 1.93)
Prostate cancer	874 (3.5)	253 (5.3)	0.85 (0.72 – 1.00)	2186 (3.0)	326 (6.2)	0.95 (0.82 - 1.09)
Schizophrenia and other psychotic disorders	514 (2.1)	163 (3.4)	1.64 (1.32 - 2.03)	1736 (2.4)	253 (4.8)	1.79 (1.52 - 2.11)

© 2022 Vo AD et al. *JAMA Network Open.*

Tobacco use	1930 (7.8)	469 (9.9)	1.22 (1.07 - 1.40)	6023 (8.2)	695 (13.3)	1.49 (1.34 - 1.66)
Viral hepatitis	279 (1.1)	95 (2.0)	1.07 (0.80 - 1.42)	858 (1.2)	122 (2.3)	0.89 (0.70 - 1.13)
Death from breakthrough infection	0 (0.0)	736 (15.6)		0 (0.0)	728 (13.9)	

OR = odds ratio. CI = 95% confidence interval.

© 2022 Vo AD et al. *JAMA Network Open.*

eTable 19. Subcohort of Patients Who Received the Pfizer Vaccine, Data and Analysis

	Overall	Non-severe	Severe	Multivariable OR (CI)	P (multi)
N	54164	48831	5333		
Sex (%)					
Male	47148 (87.0)	42048 (86.1)	5100 (95.6)	1.0 (referent	
Female	7016 (13.0)	6783 (13.9)	233 (4.4)	0.81 (0.69 - 0.94)	0.007 [a]
Age ranges (%)					
Less than 40	6643 (12.3)	6597 (13.5)	46 (0.9)	0.41 (0.28 - 0.61)	1.3E-05 [a]
40-45	3122 (5.8)	3082 (6.3)	40 (0.8)	0.68 (0.45 - 1.04)	0.07
45-50	3183 (5.9)	3123 (6.4)	60 (1.1)	1.0 (referent)	
50-55	4745 (8.8)	4615 (9.5)	130 (2.4)	1.38 (1.00 - 1.89)	0.05
55-60	5281 (9.8)	5040 (10.3)	241 (4.5)	2.02 (1.50 - 2.71)	3.2E-06 [a]
60-65	6181 (11.4)	5758 (11.8)	423 (7.9)	2.80 (2.11 - 3.72)	<1.0E-08 [a]
65-70	6110 (11.3)	5414 (11.1)	696 (13.1)	4.15 (3.14 - 5.48)	<1.0E-08 [a]
70-75	9020 (16.7)	7665 (15.7)	1355 (25.4)	5.63 (4.28 - 7.41)	<1.0E-08 [a]
75-80	5677 (10.5)	4629 (9.5)	1048 (19.7)	7.14 (5.40 - 9.44)	<1.0E-08 [a]
80 or greater	4202 (7.8)	2908 (6.0)	1294 (24.3)	13.1 (9.85 - 17.3)	<1.0E-08 [a]
Race (%)					
American Indian or Alaska Native	436 (0.8)	396 (0.8)	40 (0.8)	1.06 (0.71 - 1.56)	0.78
Asian	859 (1.6)	824 (1.7)	35 (0.7)	0.88 (0.59 - 1.32)	0.54
Black or African American	15308 (28.3)	14009 (28.7)	1299 (24.4)	0.96 (0.89 - 1.05)	0.39
Native Hawaiian or other Pacific Islander	580 (1.1)	536 (1.1)	44 (0.8)	0.93 (0.65 - 1.34)	0.71
White	32713 (60.4)	29112 (59.6)	3601 (67.5)	1.0 (referent)	
Unknown	4268 (7.9)	3954 (8.1)	314 (5.9)	0.95 (0.82 - 1.11)	0.54
Ethnicity Hispanic/Latino (%)					
Hispanic or Latino	5442 (10.0)	5030 (10.3)	412 (7.7)	1.13 (1.00 - 1.29)	0.05
Not Hispanic or Latino	45641 (84.3)	40919 (83.8)	4722 (88.5)	1.0 (referent)	
Unknown	3081 (5.7)	2882 (5.9)	199 (3.7)	1.01 (0.83 - 1.22)	0.94
US Region (%)					
Continental	8636 (15.9)	7663 (15.7)	973 (18.2)	1.26 (1.13 - 1.40)	2.0E-05 [a]
Midwest	10398 (19.2)	9298 (19.0)	1100 (20.6)	1.01 (0.91 - 1.12)	0.83
North Atlantic	11767 (21.7)	10654 (21.8)	1113 (20.9)	1.05 (0.95 - 1.16)	0.37
Pacific	10037 (18.5)	9178 (18.8)	859 (16.1)	0.99 (0.89 - 1.11)	0.92
Southeast	11891 (22.0)	10618 (21.7)	1273 (23.9)	1.0 (referent)	
Unknown	1435 (2.6)	1420 (2.9)	15 (0.3)	0.29 (0.16 - 0.53)	6.7E-05 [a]
Period of dominant variant (%)					
Pre-delta period	1560 (2.9)	1204 (2.5)	356 (6.7)	1.0 (referent)	

© 2022 Vo AD et al. *JAMA Network Open.*

Delta period	14235 (26.3)	11825 (24.2)	2410 (45.2)	0.97 (0.80 - 1.18)	0.78
Omicron period	38369 (70.8)	35802 (73.3)	2567 (48.1)	0.45 (0.37 - 0.56)	<1.0E-08 [a]
History of infection before vaccinated (%)	2122 (3.9)	1915 (3.9)	207 (3.9)	0.73 (0.61 - 0.88)	8.5E-04 [a]
Months since full vax at breakthrough (%)					
4 or less	6089 (11.2)	5375 (11.0)	714 (13.4)	1.0 (referent)	
4-5	3473 (6.4)	3016 (6.2)	457 (8.6)	1.33 (1.12 - 1.57)	0.001 [a]
5-6	3934 (7.3)	3346 (6.9)	588 (11.0)	1.09 (0.93 - 1.29)	0.29
6-7	4244 (7.8)	3657 (7.5)	587 (11.0)	1.15 (0.97 - 1.36)	0.10
7-8	5464 (10.1)	4951 (10.1)	513 (9.6)	1.29 (1.09 - 1.53)	0.003 [a]
8-9	9310 (17.2)	8736 (17.9)	574 (10.8)	1.35 (1.14 - 1.60)	4.3E-04 [a]
9-10	9193 (17.0)	8511 (17.4)	682 (12.8)	1.41 (1.18 - 1.68)	1.2E-04 [a]
10-11	8167 (15.1)	7395 (15.1)	772 (14.5)	1.50 (1.25 - 1.80)	1.3E-05 [a]
11-12	3710 (6.8)	3328 (6.8)	382 (7.2)	1.75 (1.42 - 2.15)	1.1E-07 [a]
12 or more	580 (1.1)	516 (1.1)	64 (1.2)	1.81 (1.27 - 2.58)	0.001 [a]
Months since boosted at breakthrough (%)					
Not boosted	38038 (70.2)	33869 (69.4)	4169 (78.2)	1.0 (referent)	
1 or less	2596 (4.8)	2422 (5.0)	174 (3.3)	0.55 (0.46 - 0.66)	<1.0E-08 [a]
1-2	3113 (5.7)	2942 (6.0)	171 (3.2)	0.45 (0.38 - 0.55)	<1.0E-08 [a]
2-3	5230 (9.7)	4907 (10.0)	323 (6.1)	0.43 (0.37 - 0.50)	<1.0E-08 [a]
3-4	3410 (6.3)	3097 (6.3)	313 (5.9)	0.54 (0.46 - 0.63)	<1.0E-08 [a]
4-5	1078 (2.0)	957 (2.0)	121 (2.3)	0.55 (0.43 - 0.70)	9.01E-07 [a]
5-6	187 (0.3)	160 (0.3)	27 (0.5)	0.70 (0.43 - 1.14)	0.15
6 or more	512 (0.9)	477 (1.0)	35 (0.7)	0.72 (0.49 - 1.07)	0.11
Immune-suppressive medications before breakthrough (%)					
Before breakthrough Chemotherapy	543 (1.0)	368 (0.8)	175 (3.3)	2.66 (2.04 - 3.47)	<1.0E-08 [a]
Before breakthrough Cytokine-blocking	803 (1.5)	696 (1.4)	107 (2.0)	1.62 (1.15 - 2.29)	0.006 [a]
Before breakthrough Glucocorticoids	3518 (6.5)	2613 (5.4)	905 (17.0)	2.54 (2.29 - 2.82)	<1.0E-08 [a]
Before breakthrough Leukocyte-inhibitory	958 (1.8)	718 (1.5)	240 (4.5)	2.42 (1.91 - 3.07)	<1.0E-08 [a]
Before breakthrough Lymphocyte-depleting	309 (0.6)	218 (0.4)	91 (1.7)	1.85 (1.24 - 2.76)	0.002 [a]
Immune-suppressive medications before initial vaccination (%)					
Before vaccine Chemotherapy	233 (0.4)	167 (0.3)	66 (1.2)	0.76 (0.49 - 1.17)	0.21
Before vaccine Cytokine-blocking	627 (1.2)	560 (1.1)	67 (1.3)	0.65 (0.43 – 1.00)	0.05
Before vaccine Glucocorticoids	1353 (2.5)	1045 (2.1)	308 (5.8)	1.18 (0.99 - 1.40)	0.07
Before vaccine Leukocyte-inhibitory	679 (1.3)	545 (1.1)	134 (2.5)	0.80 (0.58 - 1.09)	0.15
Before vaccine Lymphocyte-depleting	107 (0.2)	78 (0.2)	29 (0.5)	0.90 (0.45 - 1.78)	0.75
Persistent positive test (%)	229 (0.4)	181 (0.4)	48 (0.9)	1.31 (0.92 - 1.86)	0.14
BMI Class (%)					

© 2022 Vo AD et al. *JAMA Network Open.*

Underweight	353 (0.7)	255 (0.5)	98 (1.8)	1.65 (1.23 - 2.22)	9.4E-04 [a]
Normal	7108 (13.1)	6065 (12.4)	1043 (19.6)	1.0 (referent)	
Overweight	16077 (29.7)	14622 (29.9)	1455 (27.3)	0.74 (0.67 - 0.82)	1.8E-08 [a]
Obesity I	15048 (27.8)	13728 (28.1)	1320 (24.8)	0.84 (0.76 - 0.94)	0.002 [a]
Obesity II	8248 (15.2)	7485 (15.3)	763 (14.3)	0.98 (0.86 - 1.10)	0.69
Severe Obesity	5073 (9.4)	4504 (9.2)	569 (10.7)	1.37 (1.20 - 1.57)	6.0E-06 [a]
Unknown	2257 (4.2)	2172 (4.4)	85 (1.6)	0.88 (0.67 - 1.15)	0.34
Comorbidities (%)					
Acquired hypothyroidism	2201 (4.1)	1842 (3.8)	359 (6.7)	0.95 (0.82 - 1.10)	0.47
Acute myocardial infarction	388 (0.7)	277 (0.6)	111 (2.1)	1.07 (0.80 - 1.42)	0.66
Alzheimers disease and related disorders or senile dementia	1493 (2.8)	925 (1.9)	568 (10.7)	1.87 (1.62 - 2.14)	<1.0E-08 [a]
Anemia	3987 (7.4)	3048 (6.2)	939 (17.6)	1.16 (1.04 - 1.29)	0.009 [a]
Anxiety disorders	12628 (23.3)	11673 (23.9)	955 (17.9)	0.97 (0.88 - 1.06)	0.48
Asthma	1713 (3.2)	1549 (3.2)	164 (3.1)	1.04 (0.85 - 1.26)	0.72
Atrial fibrillation	2770 (5.1)	2001 (4.1)	769 (14.4)	1.29 (1.15 - 1.44)	1.9E-05 [a]
Bipolar disorder	2972 (5.5)	2676 (5.5)	296 (5.6)	1.16 (0.99 - 1.36)	0.07
Chronic kidney disease	5692 (10.5)	4275 (8.8)	1417 (26.6)	1.64 (1.48 - 1.80)	<1.0E-08 [a]
Chronic obstructive pulmonary disease and bronchiectasis	3693 (6.8)	2676 (5.5)	1017 (19.1)	1.63 (1.47 - 1.80)	<1.0E-08 [a]
Colorectal cancer	319 (0.6)	255 (0.5)	64 (1.2)	1.02 (0.73 - 1.43)	0.90
Depression	10761 (19.9)	9803 (20.1)	958 (18.0)	1.00 (0.90 - 1.10)	0.95
Diabetes	12310 (22.7)	10243 (21.0)	2067 (38.8)	1.25 (1.15 - 1.35)	9.3E-08 [a]
Epilepsy	529 (1.0)	414 (0.8)	115 (2.2)	1.55 (1.20 - 2.01)	8.1E-04 [a]
Heart failure	2684 (5.0)	1807 (3.7)	877 (16.4)	1.69 (1.50 - 1.90)	<1.0E-08 [a]
HIV/AIDS	492 (0.9)	439 (0.9)	53 (1.0)	1.32 (0.93 - 1.86)	0.12
Hyperlipidemia	14228 (26.3)	12324 (25.2)	1904 (35.7)	0.85 (0.79 - 0.93)	2.9E-04 [a]
Hypertension	18346 (33.9)	15488 (31.7)	2858 (53.6)	1.08 (1.00 - 1.18)	0.06
Ischemic heart disease	5126 (9.5)	4012 (8.2)	1114 (20.9)	0.96 (0.86 - 1.06)	0.43
Leukemias and lymphomas	631 (1.2)	455 (0.9)	176 (3.3)	1.71 (1.35 - 2.17)	7.8E-06 [a]
Liver disease: cirrhosis and other liver conditions except viral hepatitis	2094 (3.9)	1768 (3.6)	326 (6.1)	1.24 (1.07 - 1.45)	0.005 [a]
Lung cancer	410 (0.8)	281 (0.6)	129 (2.4)	1.60 (1.23 - 2.06)	3.8E-04 [a]
Mobility impairments	565 (1.0)	375 (0.8)	190 (3.6)	2.29 (1.83 - 2.86)	<1.0E-08 [a]
Multiple sclerosis and transverse myelitis	234 (0.4)	192 (0.4)	42 (0.8)	2.44 (1.60 - 3.72)	3.5E-05 [a]
Peripheral vascular disease	1521 (2.8)	1100 (2.3)	421 (7.9)	1.13 (0.97 - 1.31)	0.11
Pressure and chronic ulcers	676 (1.2)	436 (0.9)	240 (4.5)	1.46 (1.19 - 1.80)	3.3E-04 [a]
Prostate cancer	2027 (3.7)	1689 (3.5)	338 (6.3)	0.88 (0.76 - 1.02)	0.08
Schizophrenia and other psychotic disorders	1343 (2.5)	1130 (2.3)	213 (4.0)	1.60 (1.33 - 1.92)	4.7E-07 [a]

© 2022 Vo AD et al. *JAMA Network Open.*

Tobacco use	4256 (7.9)	3709 (7.6)	547 (10.3)	1.34 (1.19 - 1.52)	1.6E-06 [a]
Viral hepatitis	754 (1.4)	623 (1.3)	131 (2.5)	0.96 (0.75 - 1.22)	0.72
Death from breakthrough infection	772 (1.4)	0 (0.0)	772 (14.5)		

[a] Meets criteria for statistical significance after adjustment for multiple comparisons, P<0.03 in this analysis. OR = odds ratio. CI = 95% confidence interval.

© 2022 Vo AD et al. *JAMA Network Open.*

eTable 20. Subcohort of Patients Who Received the Moderna Vaccine, Data and Analysis

	Overall	Non-severe	Severe	Multivariable OR (CI)	P (multi)
N	45530	41289	4241		
Sex (%)					
Male	40717 (89.4)	36586 (88.6)	4131 (97.4)	1.0 (referent	
Female	4813 (10.6)	4703 (11.4)	110 (2.6)	0.59 (0.47 - 0.72)	6.5E-07 [a]
Age ranges (%)					
Less than 40	4626 (10.2)	4591 (11.1)	35 (0.8)	0.74 (0.44 - 1.23)	0.25
40-45	2257 (5.0)	2227 (5.4)	30 (0.7)	1.22 (0.71 - 2.07)	0.47
45-50	2268 (5.0)	2241 (5.4)	27 (0.6)	1.0 (referent)	
50-55	3602 (7.9)	3521 (8.5)	81 (1.9)	1.72 (1.09 - 2.70)	0.02 [a]
55-60	4218 (9.3)	4064 (9.8)	154 (3.6)	2.58 (1.68 - 3.94)	1.4E-05 [a]
60-65	5238 (11.5)	4919 (11.9)	319 (7.5)	3.59 (2.38 - 5.41)	<1.0E-08 [a]
65-70	5209 (11.4)	4734 (11.5)	475 (11.2)	4.80 (3.20 - 7.22)	<1.0E-08 [a]
70-75	8139 (17.9)	7111 (17.2)	1028 (24.2)	6.68 (4.47 - 9.99)	<1.0E-08 [a]
75-80	5336 (11.7)	4505 (10.9)	831 (19.6)	8.50 (5.66 - 12.8)	<1.0E-08 [a]
80 or greater	4637 (10.2)	3376 (8.2)	1261 (29.7)	15.0 (10.0 - 22.6)	<1.0E-08 [a]
Race (%)					
American Indian or Alaska Native	363 (0.8)	328 (0.8)	35 (0.8)	1.44 (0.96 - 2.16)	0.08
Asian	577 (1.3)	561 (1.4)	16 (0.4)	0.69 (0.39 - 1.22)	0.20
Black or African American	9178 (20.2)	8559 (20.7)	619 (14.6)	0.91 (0.81 - 1.01)	0.08
Native Hawaiian or other Pacific Islander	460 (1.0)	437 (1.1)	23 (0.5)	0.58 (0.34 - 0.99)	0.05
White	31634 (69.5)	28296 (68.5)	3338 (78.7)	1.0 (referent)	
Unknown	3318 (7.3)	3108 (7.5)	210 (5.0)	0.88 (0.73 - 1.05)	0.15
Ethnicity Hispanic/Latino (%)					
Hispanic or Latino	4742 (10.4)	4466 (10.8)	276 (6.5)	0.91 (0.78 - 1.06)	0.24
Not Hispanic or Latino	38505 (84.6)	34687 (84.0)	3818 (90.0)	1.0 (referent)	
Unknown	2283 (5.0)	2136 (5.2)	147 (3.5)	1.11 (0.89 - 1.38)	0.35
US Region (%)					
Continental	7586 (16.7)	6862 (16.6)	724 (17.1)	1.06 (0.94 - 1.20)	0.32
Midwest	9220 (20.3)	8204 (19.9)	1016 (24.0)	0.96 (0.86 - 1.08)	0.50
North Atlantic	9887 (21.7)	8974 (21.7)	913 (21.5)	0.85 (0.76 - 0.96)	0.007 [a]
Pacific	8057 (17.7)	7393 (17.9)	664 (15.7)	0.93 (0.82 - 1.06)	0.27
Southeast	10068 (22.1)	9149 (22.2)	919 (21.7)	1.0 (referent)	
Unknown	712 (1.6)	707 (1.7)	5 (0.1)	0.31 (0.13 - 0.77)	0.01 [a]
Period of dominant variant (%)					
Pre-delta period	1117 (2.5)	872 (2.1)	245 (5.8)	1.0 (referent)	

© 2022 Vo AD et al. *JAMA Network Open.*

Delta period	11809 (25.9)	9968 (24.1)	1841 (43.4)	0.70 (0.56 - 0.89)	0.003 [a]
Omicron period	32604 (71.6)	30449 (73.7)	2155 (50.8)	0.40 (0.31 - 0.51)	<1.0E-08 [a]
History of infection before vaccinated (%)	1747 (3.8)	1576 (3.8)	171 (4.0)	0.88 (0.72 - 1.07)	0.20
Months since full vax at breakthrough (%)					
4 or less	4006 (8.8)	3537 (8.6)	469 (11.1)	1.0 (referent)	
4-5	2487 (5.5)	2203 (5.3)	284 (6.7)	1.07 (0.87 - 1.33)	0.52
5-6	3047 (6.7)	2633 (6.4)	414 (9.8)	1.12 (0.92 - 1.38)	0.27
6-7	3492 (7.7)	3054 (7.4)	438 (10.3)	1.24 (1.01 - 1.52)	0.04
7-8	4932 (10.8)	4484 (10.9)	448 (10.6)	1.35 (1.10 - 1.64)	0.003 [a]
8-9	8236 (18.1)	7672 (18.6)	564 (13.3)	1.27 (1.04 - 1.54)	0.02 [a]
9-10	9136 (20.1)	8498 (20.6)	638 (15.0)	1.22 (0.99 - 1.49)	0.06
10-11	7132 (15.7)	6459 (15.6)	673 (15.9)	1.56 (1.26 - 1.93)	4.1E-05 [a]
11-12	2772 (6.1)	2492 (6.0)	280 (6.6)	1.72 (1.34 - 2.19)	1.5E-05 [a]
12 or more	290 (0.6)	257 (0.6)	33 (0.8)	2.16 (1.38 - 3.40)	7.9E-04 [a]
Months since boosted at breakthrough (%)					
Not boosted	33195 (72.9)	29763 (72.1)	3432 (80.9)	1.0 (referent)	
1 or less	2657 (5.8)	2506 (6.1)	151 (3.6)	0.60 (0.49 - 0.72)	1.7E-07 [a]
1-2	4145 (9.1)	3956 (9.6)	189 (4.5)	0.41 (0.35 - 0.49)	<1.0E-08 [a]
2-3	3084 (6.8)	2871 (7.0)	213 (5.0)	0.47 (0.40 - 0.57)	<1.0E-08 [a]
3-4	1186 (2.6)	1067 (2.6)	119 (2.8)	0.57 (0.45 - 0.72)	3.7E-06
4-5	664 (1.5)	579 (1.4)	85 (2.0)	0.70 (0.52 - 0.93)	0.01 [a]
5-6	180 (0.4)	157 (0.4)	23 (0.5)	0.76 (0.46 - 1.26)	0.29
6 or more	419 (0.9)	390 (0.9)	29 (0.7)	0.77 (0.49 - 1.21)	0.26
Immune-suppressive medications before breakthrough (%)					
Before breakthrough Chemotherapy	398 (0.9)	282 (0.7)	116 (2.7)	2.39 (1.77 - 3.23)	1.3E-08 [a]
Before breakthrough Cytokine-blocking	682 (1.5)	608 (1.5)	74 (1.7)	1.30 (0.88 - 1.93)	0.19
Before breakthrough Glucocorticoids	3338 (7.3)	2573 (6.2)	765 (18.0)	2.13 (1.90 - 2.37)	<1.0E-08 [a]
Before breakthrough Leukocyte-inhibitory	836 (1.8)	619 (1.5)	217 (5.1)	2.57 (1.97 - 3.37)	<1.0E-08 [a]
Before breakthrough Lymphocyte-depleting	246 (0.5)	164 (0.4)	82 (1.9)	1.60 (0.99 - 2.59)	0.05
Immune-suppressive medications before initial vaccination (%)					
Before vaccine Chemotherapy	173 (0.4)	134 (0.3)	39 (0.9)	0.74 (0.45 - 1.23)	0.25
Before vaccine Cytokine-blocking	485 (1.1)	443 (1.1)	42 (1.0)	0.77 (0.47 - 1.25)	0.29
Before vaccine Glucocorticoids	1154 (2.5)	877 (2.1)	277 (6.5)	1.51 (1.26 - 1.82)	8.5E-06 [a]
Before vaccine Leukocyte-inhibitory	589 (1.3)	472 (1.1)	117 (2.8)	0.81 (0.58 - 1.13)	0.22
Before vaccine Lymphocyte-depleting	97 (0.2)	58 (0.1)	39 (0.9)	2.31 (1.14 - 4.68)	0.02 [a]
Persistent positive test (%)	181 (0.4)	133 (0.3)	48 (1.1)	1.95 (1.34 - 2.85)	5.1E-04 [a]
BMI Class (%)					

© 2022 Vo AD et al. *JAMA Network Open.*

Underweight	261 (0.6)	192 (0.5)	69 (1.6)	1.41 (0.99 - 2.02)	0.06
Normal	5872 (12.9)	4989 (12.1)	883 (20.8)	1.0 (referent)	
Overweight	13659 (30.0)	12443 (30.1)	1216 (28.7)	0.72 (0.64 - 0.80)	<1.0E-08 [a]
Obesity I	13032 (28.6)	12021 (29.1)	1011 (23.8)	0.74 (0.65 - 0.83)	3.7E-07 [a]
Obesity II	6842 (15.0)	6246 (15.1)	596 (14.1)	0.93 (0.81 - 1.07)	0.32
Severe Obesity	4349 (9.6)	3934 (9.5)	415 (9.8)	1.12 (0.96 - 1.30)	0.16
Unknown	1515 (3.3)	1464 (3.5)	51 (1.2)	0.79 (0.57 - 1.09)	0.15
Comorbidities (%)					
Acquired hypothyroidism	2301 (5.1)	1943 (4.7)	358 (8.4)	1.10 (0.95 - 1.27)	0.22
Acute myocardial infarction	304 (0.7)	231 (0.6)	73 (1.7)	1.14 (0.81 - 1.60)	0.44
Alzheimers disease and related disorders or senile dementia	1309 (2.9)	848 (2.1)	461 (10.9)	1.99 (1.72 - 2.31)	<1.0E-08 [a]
Anemia	3402 (7.5)	2650 (6.4)	752 (17.7)	1.25 (1.11 - 1.40)	2.7E-04 [a]
Anxiety disorders	10546 (23.2)	9821 (23.8)	725 (17.1)	0.83 (0.74 - 0.93)	0.001 [a]
Asthma	1414 (3.1)	1299 (3.1)	115 (2.7)	0.87 (0.69 - 1.10)	0.24
Atrial fibrillation	2587 (5.7)	1975 (4.8)	612 (14.4)	1.13 (1.00 - 1.28)	0.06
Bipolar disorder	2508 (5.5)	2255 (5.5)	253 (6.0)	1.35 (1.14 - 1.60)	5.6E-04 [a]
Chronic kidney disease	5041 (11.1)	3993 (9.7)	1048 (24.7)	1.36 (1.22 - 1.52)	2.0E-08 [a]
Chronic obstructive pulmonary disease and bronchiectasis	3827 (8.4)	2840 (6.9)	987 (23.3)	1.82 (1.64 - 2.02)	<1.0E-08 [a]
Colorectal cancer	240 (0.5)	194 (0.5)	46 (1.1)	1.06 (0.72 - 1.55)	0.77
Depression	9087 (20.0)	8286 (20.1)	801 (18.9)	1.17 (1.05 - 1.31)	0.005 [a]
Diabetes	11381 (25.0)	9708 (23.5)	1673 (39.4)	1.26 (1.16 - 1.38)	2.4E-07 [a]
Epilepsy	435 (1.0)	355 (0.9)	80 (1.9)	1.57 (1.16 - 2.12)	0.003 [a]
Heart failure	2313 (5.1)	1594 (3.9)	719 (17.0)	1.78 (1.57 - 2.03)	<1.0E-08 [a]
HIV/AIDS	305 (0.7)	282 (0.7)	23 (0.5)	1.29 (0.79 - 2.11)	0.30
Hyperlipidemia	13758 (30.2)	12241 (29.6)	1517 (35.8)	0.76 (0.69 - 0.83)	<1.0E-08 [a]
Hypertension	16693 (36.7)	14451 (35.0)	2242 (52.9)	1.05 (0.96 - 1.15)	0.32
Ischemic heart disease	4930 (10.8)	3992 (9.7)	938 (22.1)	0.98 (0.88 - 1.09)	0.70
Leukemias and lymphomas	620 (1.4)	472 (1.1)	148 (3.5)	1.58 (1.23 - 2.02)	3.1E-04 [a]
Liver disease: cirrhosis and other liver conditions except viral hepatitis	1589 (3.5)	1357 (3.3)	232 (5.5)	1.46 (1.22 - 1.75)	3.7E-05 [a]
Lung cancer	373 (0.8)	266 (0.6)	107 (2.5)	1.40 (1.06 - 1.86)	0.02 [a]
Mobility impairments	380 (0.8)	288 (0.7)	92 (2.2)	1.73 (1.30 - 2.31)	1.9E-04 [a]
Multiple sclerosis and transverse myelitis	187 (0.4)	144 (0.3)	43 (1.0)	3.99 (2.62 - 6.06)	<1.0E-08 [a]
Peripheral vascular disease	1327 (2.9)	975 (2.4)	352 (8.3)	1.29 (1.10 - 1.52)	0.002 [a]
Pressure and chronic ulcers	542 (1.2)	358 (0.9)	184 (4.3)	1.57 (1.24 - 1.98)	1.7E-04 [a]
Prostate cancer	1551 (3.4)	1304 (3.2)	247 (5.8)	0.98 (0.83 - 1.16)	0.81
Schizophrenia and other psychotic disorders	1107 (2.4)	933 (2.3)	174 (4.1)	1.87 (1.53 - 2.28)	<1.0E-08 [a]

© 2022 Vo AD et al. *JAMA Network Open.*

Tobacco use	3657 (8.0)	3180 (7.7)	477 (11.2)	1.48 (1.30 - 1.68)	<1.0E-08 [a]
Viral hepatitis	504 (1.1)	427 (1.0)	77 (1.8)	1.04 (0.77 - 1.41)	0.80
Death from breakthrough infection	646 (1.4)	0 (0.0)	646 (15.2)		

[a] Meets criteria for statistical significance after adjustment for multiple comparisons, P<0.03 in this analysis. OR = odds ratio. CI = 95% confidence interval.

© 2022 Vo AD et al. *JAMA Network Open.*

eTable 21. Subcohort of Patients With Rural Residence, Data and Analysis

	Overall	Non-severe	Severe	Multivariable OR (CI)	P (multi)
N	24572	21801	2771		
Sex (%)					
Male	22335 (90.9)	19649 (90.1)	2686 (96.9)	1.0 (referent)	
Female	2237 (9.1)	2152 (9.9)	85 (3.1)	0.93 (0.73 - 1.19)	0.58
Age ranges (%)					
Less than 40	1915 (7.8)	1896 (8.7)	19 (0.7)	0.58 (0.31 - 1.10)	0.10
40-45	1031 (4.2)	1016 (4.7)	15 (0.5)	0.84 (0.43 - 1.65)	0.62
45-50	1216 (4.9)	1195 (5.5)	21 (0.8)	1.0 (referent)	
50-55	1914 (7.8)	1866 (8.6)	48 (1.7)	1.28 (0.75 - 2.17)	0.36
55-60	2285 (9.3)	2183 (10.0)	102 (3.7)	2.06 (1.27 - 3.35)	0.003 [a]
60-65	2626 (10.7)	2429 (11.1)	197 (7.1)	3.20 (2.01 - 5.09)	9.2E-07 [a]
65-70	2691 (11.0)	2398 (11.0)	293 (10.6)	4.36 (2.75 - 6.90)	<1.0E-08 [a]
70-75	5199 (21.2)	4432 (20.3)	767 (27.7)	6.12 (3.90 - 9.59)	<1.0E-08 [a]
75-80	3246 (13.2)	2650 (12.2)	596 (21.5)	8.20 (5.20 - 12.9)	<1.0E-08 [a]
80 or greater	2449 (10.0)	1736 (8.0)	713 (25.7)	14.0 (8.82 - 22.1)	<1.0E-08 [a]
Race (%)					
American Indian or Alaska Native	247 (1.0)	205 (0.9)	42 (1.5)	1.85 (1.23 - 2.77)	0.003 [a]
Asian	95 (0.4)	94 (0.4)	1 (0.0)	0.24 (0.03 - 1.78)	0.16
Black or African American	2834 (11.5)	2629 (12.1)	205 (7.4)	0.91 (0.76 - 1.08)	0.27
Native Hawaiian or other Pacific Islander	147 (0.6)	133 (0.6)	14 (0.5)	0.59 (0.28 - 1.26)	0.17
White	19817 (80.6)	17433 (80.0)	2384 (86.0)	1.0 (referent)	
Unknown	1432 (5.8)	1307 (6.0)	125 (4.5)	0.71 (0.56 - 0.90)	0.006 [a]
Ethnicity Hispanic/Latino (%)					
Hispanic or Latino	1049 (4.3)	957 (4.4)	92 (3.3)	1.33 (1.02 - 1.72)	0.03
Not Hispanic or Latino	22413 (91.2)	19840 (91.0)	2573 (92.9)	1.0 (referent)	
Unknown	1110 (4.5)	1004 (4.6)	106 (3.8)	1.56 (1.20 - 2.04)	9.5E-04 [a]
US Region (%)					
Continental	4368 (17.8)	3771 (17.3)	597 (21.5)		
Midwest	6679 (27.2)	5976 (27.4)	703 (25.4)		
North Atlantic	5705 (23.2)	5118 (23.5)	587 (21.2)		
Pacific	2768 (11.3)	2498 (11.5)	270 (9.7)		
Southeast	5052 (20.6)	4438 (20.4)	614 (22.2)		
Unknown	247 (1.0)	205 (0.9)	42 (1.5)		
Period of dominant variant (%)					
Pre-delta period	738 (3.0)	565 (2.6)	173 (6.2)	1.0 (referent)	

© 2022 Vo AD et al. *JAMA Network Open.*

Delta period	8300 (33.8)	6942 (31.8)	1358 (49.0)	0.73 (0.56 - 0.95)	0.02 [a]
Omicron period	15534 (63.2)	14294 (65.6)	1240 (44.7)	0.40 (0.30 - 0.53)	<1.0E-08 [a]
Vaccine type (%)					
Janssen	3076 (12.5)	2755 (12.6)	321 (11.6)	1.19 (1.01 - 1.4)	0.03
Moderna	12470 (50.7)	11094 (50.9)	1376 (49.7)	0.77 (0.70 - 0.85)	3.6E-07 [a]
Pfizer	9026 (36.7)	7952 (36.5)	1074 (38.8)	1.0 (referent)	
History of infection before vaccinated (%)	832 (3.4)	744 (3.4)	88 (3.2)	0.56 (0.42 - 0.75)	7.2E-05 [a]
Months since full vax at breakthrough (%)					
4 or less	2810 (11.4)	2410 (11.1)	400 (14.4)	1.0 (referent)	
4-5	1712 (7.0)	1507 (6.9)	205 (7.4)	0.91 (0.72 - 1.15)	0.44
5-6	2094 (8.5)	1771 (8.1)	323 (11.7)	1.06 (0.86 - 1.32)	0.58
6-7	2274 (9.3)	1959 (9.0)	315 (11.4)	0.98 (0.79 - 1.22)	0.86
7-8	2506 (10.2)	2216 (10.2)	290 (10.5)	0.99 (0.80 - 1.24)	0.96
8-9	3757 (15.3)	3438 (15.8)	319 (11.5)	1.05 (0.84 - 1.32)	0.64
9-10	4242 (17.3)	3891 (17.8)	351 (12.7)	1.07 (0.84 - 1.35)	0.59
10-11	3392 (13.8)	3023 (13.9)	369 (13.3)	1.25 (0.97 - 1.61)	0.08
11-12	1564 (6.4)	1389 (6.4)	175 (6.3)	1.26 (0.94 - 1.69)	0.12
12 or more	221 (0.9)	197 (0.9)	24 (0.9)	1.43 (0.84 - 2.46)	0.19
Months since boosted at breakthrough (%)					
Not boosted	18995 (77.3)	16687 (76.5)	2308 (83.3)	1.0 (referent)	
1 or less	1057 (4.3)	972 (4.5)	85 (3.1)	0.70 (0.54 - 0.91)	0.008
1-2	1384 (5.6)	1295 (5.9)	89 (3.2)	0.57 (0.44 - 0.73)	1.2E-05 [a]
2-3	1654 (6.7)	1526 (7.0)	128 (4.6)	0.52 (0.41 - 0.65)	2.6E-08 [a]
3-4	899 (3.7)	803 (3.7)	96 (3.5)	0.68 (0.52 - 0.89)	0.004 [a]
4-5	347 (1.4)	311 (1.4)	36 (1.3)	0.47 (0.30 - 0.71)	3.9E-04 [a]
5-6	91 (0.4)	77 (0.4)	14 (0.5)	0.87 (0.45 - 1.68)	0.68
6 or more	145 (0.6)	130 (0.6)	15 (0.5)	0.86 (0.47 - 1.59)	0.63
Immune-suppressive medications before breakthrough (%)					
Before breakthrough Chemotherapy	239 (1.0)	163 (0.7)	76 (2.7)	2.40 (1.62 - 3.56)	1.4E-05 [a]
Before breakthrough Cytokine-blocking	399 (1.6)	340 (1.6)	59 (2.1)	2.07 (1.30 - 3.31)	0.002 [a]
Before breakthrough Glucocorticoids	2009 (8.2)	1510 (6.9)	499 (18.0)	2.05 (1.79 - 2.35)	<1.0E-08 [a]
Before breakthrough Leukocyte-inhibitory	506 (2.1)	371 (1.7)	135 (4.9)	2.71 (1.94 - 3.78)	<1.0E-08 [a]
Before breakthrough Lymphocyte-depleting	155 (0.6)	109 (0.5)	46 (1.7)	1.74 (0.96 - 3.13)	0.07
Immune-suppressive medications before initial vaccination (%)					
Before vaccine Chemotherapy	106 (0.4)	79 (0.4)	27 (1.0)	0.70 (0.37 - 1.33)	0.28
Before vaccine Cytokine-blocking	298 (1.2)	264 (1.2)	34 (1.2)	0.54 (0.30 - 0.96)	0.04
Before vaccine Glucocorticoids	719 (2.9)	562 (2.6)	157 (5.7)	1.13 (0.89 - 1.43)	0.30

© 2022 Vo AD et al. *JAMA Network Open.*

Before vaccine Leukocyte-inhibitory	354 (1.4)	278 (1.3)	76 (2.7)	0.69 (0.46 - 1.05)	0.08
Before vaccine Lymphocyte-depleting	59 (0.2)	43 (0.2)	16 (0.6)	1.11 (0.45 - 2.75)	0.82
Persistent positive test (%)	110 (0.4)	84 (0.4)	26 (0.9)	1.65 (1.02 - 2.68)	0.04
BMI Class (%)					
Underweight	127 (0.5)	91 (0.4)	36 (1.3)	1.43 (0.88 - 2.33)	0.15
Normal	2872 (11.7)	2383 (10.9)	489 (17.6)	1.0 (referent)	
Overweight	7074 (28.8)	6334 (29.1)	740 (26.7)	0.69 (0.60 - 0.80)	1.5E-06 [a]
Obesity I	7173 (29.2)	6451 (29.6)	722 (26.1)	0.80 (0.69 - 0.93)	0.004 [a]
Obesity II	4058 (16.5)	3623 (16.6)	435 (15.7)	0.98 (0.83 - 1.17)	0.85
Severe Obesity	2632 (10.7)	2312 (10.6)	320 (11.5)	1.25 (1.03 - 1.51)	0.02
Unknown	636 (2.6)	607 (2.8)	29 (1.0)	0.79 (0.50 - 1.24)	0.30
Comorbidities (%)					
Acquired hypothyroidism	1397 (5.7)	1157 (5.3)	240 (8.7)	0.99 (0.82 - 1.18)	0.89
Acute myocardial infarction	179 (0.7)	130 (0.6)	49 (1.8)	1.07 (0.70 - 1.61)	0.76
Alzheimers disease and related disorders or senile dementia	693 (2.8)	450 (2.1)	243 (8.8)	1.75 (1.43 - 2.15)	8.2E-08 [a]
Anemia	1830 (7.4)	1381 (6.3)	449 (16.2)	1.21 (1.04 - 1.41)	0.01 [a]
Anxiety disorders	5515 (22.4)	4998 (22.9)	517 (18.7)	1.02 (0.89 - 1.17)	0.78
Asthma	746 (3.0)	669 (3.1)	77 (2.8)	0.90 (0.68 - 1.18)	0.44
Atrial fibrillation	1645 (6.7)	1270 (5.8)	375 (13.5)	0.93 (0.79 - 1.09)	0.35
Bipolar disorder	1245 (5.1)	1114 (5.1)	131 (4.7)	1.13 (0.89 - 1.43)	0.32
Chronic kidney disease	2647 (10.8)	2004 (9.2)	643 (23.2)	1.45 (1.26 - 1.66)	9.7E-08 [a]
Chronic obstructive pulmonary disease and bronchiectasis	2530 (10.3)	1882 (8.6)	648 (23.4)	1.75 (1.54 - 1.99)	<1.0E-08 [a]
Colorectal cancer	141 (0.6)	121 (0.6)	20 (0.7)	0.63 (0.35 - 1.12)	0.11
Depression	4669 (19.0)	4189 (19.2)	480 (17.3)	0.97 (0.84 - 1.13)	0.70
Diabetes	6583 (26.8)	5442 (25.0)	1141 (41.2)	1.32 (1.18 - 1.47)	7.4E-07 [a]
Epilepsy	220 (0.9)	183 (0.8)	37 (1.3)	1.28 (0.82 – 2.00)	0.27
Heart failure	1393 (5.7)	944 (4.3)	449 (16.2)	1.69 (1.43 - 1.99)	<1.0E-08 [a]
HIV/AIDS	82 (0.3)	73 (0.3)	9 (0.3)	1.71 (0.78 - 3.79)	0.18
Hyperlipidemia	7882 (32.1)	6821 (31.3)	1061 (38.3)	0.82 (0.73 - 0.92)	7.0E-04 [a]
Hypertension	9459 (38.5)	7955 (36.5)	1504 (54.3)	1.09 (0.97 - 1.23)	0.13
Ischemic heart disease	3165 (12.9)	2493 (11.4)	672 (24.3)	1.02 (0.89 - 1.16)	0.79
Leukemias and lymphomas	394 (1.6)	300 (1.4)	94 (3.4)	1.37 (1.00 - 1.89)	0.05
Liver disease: cirrhosis and other liver conditions except viral hepatitis	930 (3.8)	809 (3.7)	121 (4.4)	1.07 (0.84 - 1.35)	0.60
Lung cancer	211 (0.9)	144 (0.7)	67 (2.4)	1.37 (0.95 - 1.99)	0.10
Mobility impairments	233 (0.9)	164 (0.8)	69 (2.5)	2.10 (1.49 - 2.98)	2.7E-05 [a]
Multiple sclerosis and transverse myelitis	110 (0.4)	91 (0.4)	19 (0.7)	2.15 (1.16 - 3.99)	0.01 [a]

© 2022 Vo AD et al. *JAMA Network Open.*

Peripheral vascular disease	792 (3.2)	563 (2.6)	229 (8.3)	1.31 (1.07 - 1.60)	0.009 [a]
Pressure and chronic ulcers	333 (1.4)	207 (0.9)	126 (4.5)	1.64 (1.23 - 2.20)	8.6E-04 [a]
Prostate cancer	855 (3.5)	709 (3.3)	146 (5.3)	0.95 (0.77 - 1.18)	0.64
Schizophrenia and other psychotic disorders	452 (1.8)	381 (1.7)	71 (2.6)	1.45 (1.06 - 1.98)	0.02 [a]
Tobacco use	2112 (8.6)	1810 (8.3)	302 (10.9)	1.44 (1.22 - 1.69)	1.5E-05 [a]
Viral hepatitis	193 (0.8)	161 (0.7)	32 (1.2)	1.24 (0.78 - 1.98)	0.36
Death from breakthrough infection	458 (1.9)	0 (0.0)	458 (16.5)		

[a] Meets criteria for statistical significance after adjustment for multiple comparisons, P<0.025 for this analysis. OR = odds ratio. CI = 95% confidence interval.

© 2022 Vo AD et al. *JAMA Network Open.*

eTable 22. Subcohort of Patients With Urban Residence, Data and Analysis

	Overall	Non-severe	Severe	Multivariable OR (CI)	P (multi)
N	82350	74652	7698		
Sex (%)					
Male	72204 (87.7)	64804 (86.8)	7400 (96.1)	1.0 (referent)	
Female	10146 (12.3)	9848 (13.2)	298 (3.9)	0.65 (0.57 - 0.75)	<1.0E-08 [a]
Age ranges (%)					
Less than 40	10388 (12.6)	10301 (13.8)	87 (1.1)	0.55 (0.41 - 0.75)	1.8E-04 [a]
40-45	4783 (5.8)	4716 (6.3)	67 (0.9)	0.83 (0.60 - 1.16)	0.28
45-50	4670 (5.7)	4587 (6.1)	83 (1.1)	1.0 (referent)	
50-55	7289 (8.9)	7063 (9.5)	226 (2.9)	1.66 (1.28 - 2.15)	1.6E-04 [a]
55-60	8161 (9.9)	7795 (10.4)	366 (4.8)	2.19 (1.70 - 2.81)	<1.0E-08 [a]
60-65	9969 (12.1)	9291 (12.4)	678 (8.8)	3.03 (2.38 - 3.85)	<1.0E-08 [a]
65-70	9407 (11.4)	8375 (11.2)	1032 (13.4)	4.51 (3.56 - 5.72)	<1.0E-08 [a]
70-75	12877 (15.6)	11044 (14.8)	1833 (23.8)	6.07 (4.80 - 7.67)	<1.0E-08 [a]
75-80	8179 (9.9)	6779 (9.1)	1400 (18.2)	7.59 (5.98 - 9.63)	<1.0E-08 [a]
80 or greater	6627 (8.0)	4701 (6.3)	1926 (25.0)	13.6 (10.7 - 17.3)	<1.0E-08 [a]
Race (%)					
American Indian or Alaska Native	618 (0.8)	573 (0.8)	45 (0.6)	1.09 (0.77 - 1.54)	0.62
Asian	1362 (1.7)	1307 (1.8)	55 (0.7)	0.90 (0.66 - 1.23)	0.52
Black or African American	23112 (28.1)	21180 (28.4)	1932 (25.1)	0.97 (0.90 - 1.04)	0.34
Native Hawaiian or other Pacific Islander	920 (1.1)	859 (1.2)	61 (0.8)	0.85 (0.63 - 1.15)	0.30
White	49633 (60.3)	44476 (59.6)	5157 (67.0)	1.0 (referent)	
Unknown	6705 (8.1)	6257 (8.4)	448 (5.8)	0.97 (0.85 - 1.10)	0.62
Ethnicity Hispanic/Latino (%)					
Hispanic or Latino	9526 (11.6)	8907 (11.9)	619 (8.0)	1.00 (0.91 - 1.11)	0.95
Not Hispanic or Latino	68231 (82.9)	61437 (82.3)	6794 (88.3)	1.0 (referent)	
Unknown	4593 (5.6)	4308 (5.8)	285 (3.7)	0.95 (0.82 - 1.12)	0.57
US Region (%)					
Continental	13603 (16.5)	12318 (16.5)	1285 (16.7)		
Midwest	15036 (18.3)	13430 (18.0)	1606 (20.9)		
North Atlantic	17739 (21.5)	16145 (21.6)	1594 (20.7)		
Pacific	17213 (20.9)	15782 (21.1)	1431 (18.6)		
Southeast	18759 (22.8)	16977 (22.7)	1782 (23.1)		
Unknown	618 (0.8)	573 (0.8)	45 (0.6)		
Period of dominant variant (%)					
Pre-delta period	2074 (2.5)	1624 (2.2)	450 (5.8)	1.0 (referent)	

© 2022 Vo AD et al. *JAMA Network Open.*

Delta period	20494 (24.9)	17168 (23.0)	3326 (43.2)	1.04 (0.89 - 1.22)	0.61
Omicron period	59782 (72.6)	55860 (74.8)	3922 (50.9)	0.53 (0.44 - 0.63)	<1.0E-08 [a]
Vaccine type (%)					
Janssen	7674 (9.3)	6969 (9.3)	705 (9.2)	1.34 (1.21 - 1.49)	1.3E-08 [a]
Moderna	31681 (38.5)	28884 (38.7)	2797 (36.3)	0.80 (0.75 - 0.84)	<1.0E-08 [a]
Pfizer	42995 (52.2)	38799 (52.0)	4196 (54.5)	1.0 (referent)	
History of infection before vaccinated (%)	3442 (4.2)	3121 (4.2)	321 (4.2)	0.80 (0.69 - 0.93)	0.003 [a]
Months since full vax at breakthrough (%)					
4 or less	9193 (11.2)	8099 (10.8)	1094 (14.2)	1.0 (referent)	
4-5	5277 (6.4)	4634 (6.2)	643 (8.4)	1.11 (0.97 - 1.28)	0.12
5-6	5801 (7.0)	5029 (6.7)	772 (10.0)	0.94 (0.83 - 1.08)	0.40
6-7	6357 (7.7)	5547 (7.4)	810 (10.5)	1.08 (0.94 - 1.23)	0.27
7-8	8825 (10.7)	8055 (10.8)	770 (10.0)	1.16 (1.02 - 1.32)	0.03
8-9	14677 (17.8)	13770 (18.4)	907 (11.8)	1.13 (0.99 - 1.29)	0.07
9-10	15141 (18.4)	14086 (18.9)	1055 (13.7)	1.14 (0.99 - 1.30)	0.07
10-11	11743 (14.3)	10656 (14.3)	1087 (14.1)	1.33 (1.15 - 1.54)	1.6E-04 [a]
11-12	4704 (5.7)	4218 (5.7)	486 (6.3)	1.62 (1.36 - 1.92)	3.6E-08 [a]
12 or more	632 (0.8)	558 (0.7)	74 (1.0)	1.72 (1.26 - 2.36)	7.3E-04 [a]
Months since boosted at breakthrough (%)					
Not boosted	59278 (72.0)	53135 (71.2)	6143 (79.8)	1.0 (referent)	
1 or less	4493 (5.5)	4229 (5.7)	264 (3.4)	0.53 (0.46 - 0.62)	<1.0E-08 [a]
1-2	6110 (7.4)	5815 (7.8)	295 (3.8)	0.41 (0.36 - 0.47)	<1.0E-08 [a]
2-3	6674 (8.1)	6256 (8.4)	418 (5.4)	0.44 (0.39 - 0.50)	<1.0E-08 [a]
3-4	3629 (4.4)	3294 (4.4)	335 (4.4)	0.51 (0.44 - 0.59)	<1.0E-08 [a]
4-5	1363 (1.7)	1195 (1.6)	168 (2.2)	0.63 (0.52 - 0.77)	9.2E-06 [a]
5-6	247 (0.3)	211 (0.3)	36 (0.5)	0.70 (0.46 - 1.06)	0.10
6 or more	556 (0.7)	517 (0.7)	39 (0.5)	0.67 (0.46 - 0.97)	0.03
Immune-suppressive medications before breakthrough (%)					
Before breakthrough Chemotherapy	758 (0.9)	525 (0.7)	233 (3.0)	2.55 (2.04 - 3.18)	<1.0E-08 [a]
Before breakthrough Cytokine-blocking	1166 (1.4)	1026 (1.4)	140 (1.8)	1.43 (1.06 - 1.92)	0.02 [a]
Before breakthrough Glucocorticoids	5379 (6.5)	4076 (5.5)	1303 (16.9)	2.44 (2.24 - 2.66)	<1.0E-08 [a]
Before breakthrough Leukocyte-inhibitory	1396 (1.7)	1046 (1.4)	350 (4.5)	2.41 (1.96 - 2.96)	<1.0E-08 [a]
Before breakthrough Lymphocyte-depleting	421 (0.5)	291 (0.4)	130 (1.7)	1.97 (1.40 - 2.79)	1.1E-04 [a]
Immune-suppressive medications before initial vaccination (%)					
Before vaccine Chemotherapy	316 (0.4)	230 (0.3)	86 (1.1)	0.86 (0.59 - 1.24)	0.41
Before vaccine Cytokine-blocking	889 (1.1)	809 (1.1)	80 (1.0)	0.67 (0.47 - 0.97)	0.03
Before vaccine Glucocorticoids	1998 (2.4)	1530 (2.0)	468 (6.1)	1.36 (1.18 - 1.57)	1.6E-05 [a]

© 2022 Vo AD et al. *JAMA Network Open.*

Before vaccine Leukocyte-inhibitory	1001 (1.2)	808 (1.1)	193 (2.5)	0.84 (0.65 - 1.10)	0.20
Before vaccine Lymphocyte-depleting	158 (0.2)	107 (0.1)	51 (0.7)	0.98 (0.56 - 1.72)	0.95
Persistent positive test (%)	340 (0.4)	265 (0.4)	75 (1.0)	1.46 (1.09 - 1.96)	0.01 [a]
BMI Class (%)					
Underweight	536 (0.7)	394 (0.5)	142 (1.8)	1.50 (1.17 - 1.93)	0.001 [a]
Normal	11099 (13.5)	9519 (12.8)	1580 (20.5)	1.0 (referent)	
Overweight	24588 (29.9)	22455 (30.1)	2133 (27.7)	0.76 (0.70 - 0.82)	<1.0E-08 [a]
Obesity I	22889 (27.8)	21043 (28.2)	1846 (24.0)	0.82 (0.75 - 0.90)	1.6E-05 [a]
Obesity II	12288 (14.9)	11213 (15.0)	1075 (14.0)	1.00 (0.91 - 1.11)	0.95
Severe Obesity	7734 (9.4)	6936 (9.3)	798 (10.4)	1.33 (1.18 - 1.49)	9.5E-07 [a]
Unknown	3216 (3.9)	3092 (4.1)	124 (1.6)	0.90 (0.72 - 1.11)	0.32
Comorbidities (%)					
Acquired hypothyroidism	3443 (4.2)	2904 (3.9)	539 (7.0)	1.02 (0.91 - 1.15)	0.70
Acute myocardial infarction	596 (0.7)	432 (0.6)	164 (2.1)	1.16 (0.92 - 1.47)	0.20
Alzheimers disease and related disorders or senile dementia	2291 (2.8)	1422 (1.9)	869 (11.3)	1.97 (1.76 - 2.20)	<1.0E-08 [a]
Anemia	6155 (7.5)	4755 (6.4)	1400 (18.2)	1.18 (1.08 - 1.29)	2.3E-04 [a]
Anxiety disorders	19879 (24.1)	18481 (24.8)	1398 (18.2)	0.87 (0.80 - 0.94)	3.9E-04 [a]
Asthma	2651 (3.2)	2419 (3.2)	232 (3.0)	0.97 (0.82 - 1.14)	0.70
Atrial fibrillation	4082 (5.0)	2974 (4.0)	1108 (14.4)	1.30 (1.18 - 1.43)	6.9E-08 [a]
Bipolar disorder	4884 (5.9)	4378 (5.9)	506 (6.6)	1.28 (1.14 - 1.45)	5.7E-05 [a]
Chronic kidney disease	8990 (10.9)	6902 (9.2)	2088 (27.1)	1.58 (1.46 - 1.71)	<1.0E-08 [a]
Chronic obstructive pulmonary disease and bronchiectasis	5716 (6.9)	4148 (5.6)	1568 (20.4)	1.73 (1.60 - 1.88)	<1.0E-08 [a]
Colorectal cancer	455 (0.6)	358 (0.5)	97 (1.3)	1.06 (0.81 - 1.40)	0.67
Depression	17170 (20.9)	15649 (21.0)	1521 (19.8)	1.14 (1.05 - 1.24)	0.002 [a]
Diabetes	19031 (23.1)	16066 (21.5)	2965 (38.5)	1.21 (1.13 - 1.29)	<1.0E-08 [a]
Epilepsy	838 (1.0)	665 (0.9)	173 (2.2)	1.59 (1.30 - 1.96)	9.8E-06 [a]
Heart failure	3996 (4.9)	2703 (3.6)	1293 (16.8)	1.69 (1.53 - 1.86)	<1.0E-08 [a]
HIV/AIDS	778 (0.9)	705 (0.9)	73 (0.9)	1.25 (0.94 - 1.66)	0.12
Hyperlipidemia	22868 (27.8)	20084 (26.9)	2784 (36.2)	0.82 (0.77 - 0.88)	2.5E-08 [a]
Hypertension	28651 (34.8)	24508 (32.8)	4143 (53.8)	1.08 (1.00 - 1.15)	0.04
Ischemic heart disease	7701 (9.4)	6103 (8.2)	1598 (20.8)	0.95 (0.88 - 1.04)	0.28
Leukemias and lymphomas	916 (1.1)	675 (0.9)	241 (3.1)	1.64 (1.35 – 2.00)	1.0E-06 [a]
Liver disease: cirrhosis and other liver conditions except viral hepatitis	3152 (3.8)	2663 (3.6)	489 (6.4)	1.35 (1.19 - 1.53)	2.6E-06 [a]
Lung cancer	603 (0.7)	422 (0.6)	181 (2.4)	1.53 (1.24 - 1.90)	9.7E-05 [a]
Mobility impairments	784 (1.0)	552 (0.7)	232 (3.0)	1.95 (1.61 - 2.36)	<1.0E-08 [a]
Multiple sclerosis and transverse myelitis	338 (0.4)	265 (0.4)	73 (0.9)	3.41 (2.47 - 4.71)	<1.0E-08 [a]

© 2022 Vo AD et al. *JAMA Network Open.*

Peripheral vascular disease	2275 (2.8)	1654 (2.2)	621 (8.1)	1.19 (1.05 - 1.34)	0.006 [a]
Pressure and chronic ulcers	1001 (1.2)	655 (0.9)	346 (4.5)	1.49 (1.26 - 1.77)	4.2E-06 [a]
Prostate cancer	2913 (3.5)	2431 (3.3)	482 (6.3)	0.90 (0.80 - 1.02)	0.10
Schizophrenia and other psychotic disorders	2234 (2.7)	1877 (2.5)	357 (4.6)	1.74 (1.51 – 2.00)	<1.0E-08 [a]
Tobacco use	7017 (8.5)	6123 (8.2)	894 (11.6)	1.34 (1.22 - 1.47)	<1.0E-08 [a]
Viral hepatitis	1197 (1.5)	998 (1.3)	199 (2.6)	0.90 (0.74 - 1.10)	0.30
Death from breakthrough infection	1079 (1.3)	0 (0.0)	1079 (14.0)		

[a] Meets criteria for statistical significance after adjustment for multiple comparisons, P<0.03 in this analysis. OR = odds ratio. CI = 95% confidence interval.

© 2022 Vo AD et al. *JAMA Network Open.*
